# DNMT1 function in cortical PV interneurons modulates cortical network activity, mood disorder‑related behavior, and perineuronal nets

**DOI:** 10.1038/s42003-026-11079-x

**Published:** 2026-10-08

**Authors:** Jenice Linde, Can Bora Yildiz, Katharina Vöhringer, Gerion Nabbefeld, Severin Graff, Daniel Pensold, Julia Reichard, Marie Hermanns, Laura Koch, Magdalena Sophie Müller, Ayushi Tanwar, Christoph Weber-Hamacher, Ivan G. Costa Filho, Thomas Stiehl, Marc Spehr, Björn Kampa, Anja Urbach, Simon Musall, Geraldine Zimmer-Bensch

**Affiliations:** 1 Department for Neuroepigenetics, Institute for Biology II, RWTH Aachen University, Aachen, Germany; 2 Research Training Group RTG2416 “MultiSenses – MultiScales”, RWTH Aachen University, Aachen, Germany; 3 Department for Molecular and Systemic Neurophysiology, Institute for Biology II, RWTH Aachen University, Aachen, Germany; 4 Institute of Biological Information Processing (IBI-3) Bioelectronics, Forschungszentrum Jülich, Jülich, Germany; 5 Department of Chemosensation, Institute for Biology II, RWTH Aachen University, Aachen, Germany; 6 Institute for Computational Genomics, RWTH Aachen University Hospital, Aachen, Germany; 7 Institute for Computational Biomedicine and Disease Modeling, RWTH Aachen University Hospital, Aachen, Germany; 8 Department of Neurology, Jena University Hospital, Jena, Germany

**Keywords:** Molecular neuroscience, Neural circuits

## Abstract

Parvalbuminergic (PV) interneurons are central to cortical network stability and psychiatric vulnerability. Here, we identify DNA methyltransferase 1 (DNMT1) as a key epigenetic regulator linking PV interneuron function to glial and extracellular matrix remodeling. Conditional PV-specific *Dnmt1* deletion, combined with single-nucleus RNA-seq (snRNA-seq), snATAC-seq, in vivo electrophysiology, histology, and behavioral analyses, revealed that DNMT1 loss increases PV spiking but reduces inhibitory efficacy, driving network desynchronization and depression- and anxiety-like behavior in mice. These physiological changes were accompanied by non-cell-autonomous transcriptional and chromatin alterations, affecting perineuronal net (PNN) organization and neuron-glia communication. Cell-cell communication analyses predicted reorganized perisomatic signaling, and compartmental modeling of oligodendrocyte lineage dynamics further revealed impaired formation of PNN-supporting perineuronal oligodendrocytes, consistent with a tissue-level reduction in PNN integrity. Together, our findings demonstrate that DNMT1 maintains inhibitory circuit stability through PV interneuron function, secondarily shaping glial states and extracellular scaffolds to support cortical network synchronization and affective behavior.

## Introduction

GABAergic interneurons are critical regulators of cortical circuit dynamics, ensuring precise control over pyramidal neuron activity and maintaining the excitation/inhibition (E/I) balance essential for healthy brain function. Disruption of inhibitory signaling contributes to maladaptive network states and has been implicated in a range of neuropsychiatric disorders, including schizophrenia and major depressive disorder (MDD)^1–3^. However, the etiology of such disorders is inherently multifactorial, due to interactions between genetic predispositions, environmental stressors, and epigenetic mechanisms that converge to shape disease vulnerability^3^. The exact nature of GABAergic dysfunction in MDD therefore remains debated, with contradictory findings further complicating the establishment of a unified mechanistic framework^4,5^.

Epigenetic modifications, such as DNA methylation mediated by DNA methyltransferases (DNMTs), form a crucial interface between external stimuli and neuronal function. Aberrant DNA methylation has been documented across multiple neuronal subtypes in psychiatric and neurological conditions, including schizophrenia, epilepsy, and MDD^6,7^. Among DNMTs, DNMT1 has emerged as a key regulator of inhibitory circuit function^8,9^ and altered DNMT1 expression in cortical interneurons has been linked to schizophrenia and disrupted GABAergic signaling^3^.

Within the diverse population of inhibitory neurons, parvalbumin-positive (PV) interneurons are especially important to regulate network activity. As fast-spiking interneurons, they synchronize network oscillations and gate excitatory inputs, thereby maintaining cortical E/I balance^10^. Alterations in PV interneuron number or function have been causally linked to neuropsychiatric diseases^11,12^, and their vulnerability to chronic stress makes them prime candidates for involvement in stress-related mood disorder pathology^13^. Our previous work also demonstrated that DNMT1 exerts transcriptional control in postmitotic cortical interneurons^9^ and regulates synaptic transmission in PV interneurons by modulating endocytosis-dependent GABA reuptake at presynaptic terminals, thereby fine-tuning inhibitory output^8^.

Beyond their intrinsic properties, PV interneurons are profoundly shaped by interactions with surrounding glial cells and the extracellular matrix. These include astrocytes and oligodendrocytes, as well as extracellular matrix components, such as perineuronal nets (PNNs). PNNs tightly ensheath PV interneurons, stabilize synaptic contacts, and limit plasticity, thereby influencing both inhibitory efficacy and network stability^14,15^. Disruption of glial support or PNN integrity has therefore been increasingly recognized as an important contributor to PV interneuron dysfunction^16^ and psychiatric disease vulnerability^17^.

In this study, we investigated the cell-autonomous and non-cell-autonomous consequences of DNMT1 function in PV interneurons to reveal how epigenetic dysregulation contributes to cortical circuit instability and disease vulnerability. To this end, we used conditional PV-specific *Dnmt1* knockout mice and combined single-cell transcriptomics, electrophysiology, histological analyses, and behavioral assays. *Dnmt1* deletion reduced the inhibitory impact of PV interneurons on network activity in vivo and altered oligodendroglial transcriptional programs converging on PNN regulation and neuron-glia communication. This dual effect identifies DNMT1 as a central epigenetic coordinator of inhibitory circuit function, linking intrinsic PV interneuron activity with extrinsic glial and extracellular matrix support. Together, our findings reveal how epigenetic perturbations in inhibitory neurons propagate across cellular domains, thereby destabilizing cortical networks and promoting behavioral phenotypes reminiscent of mood disorder-related symptoms.

## Results

### *Dnmt1* deletion in PV interneurons alters cortical network activity across different cortical areas

**Fig. 1:**
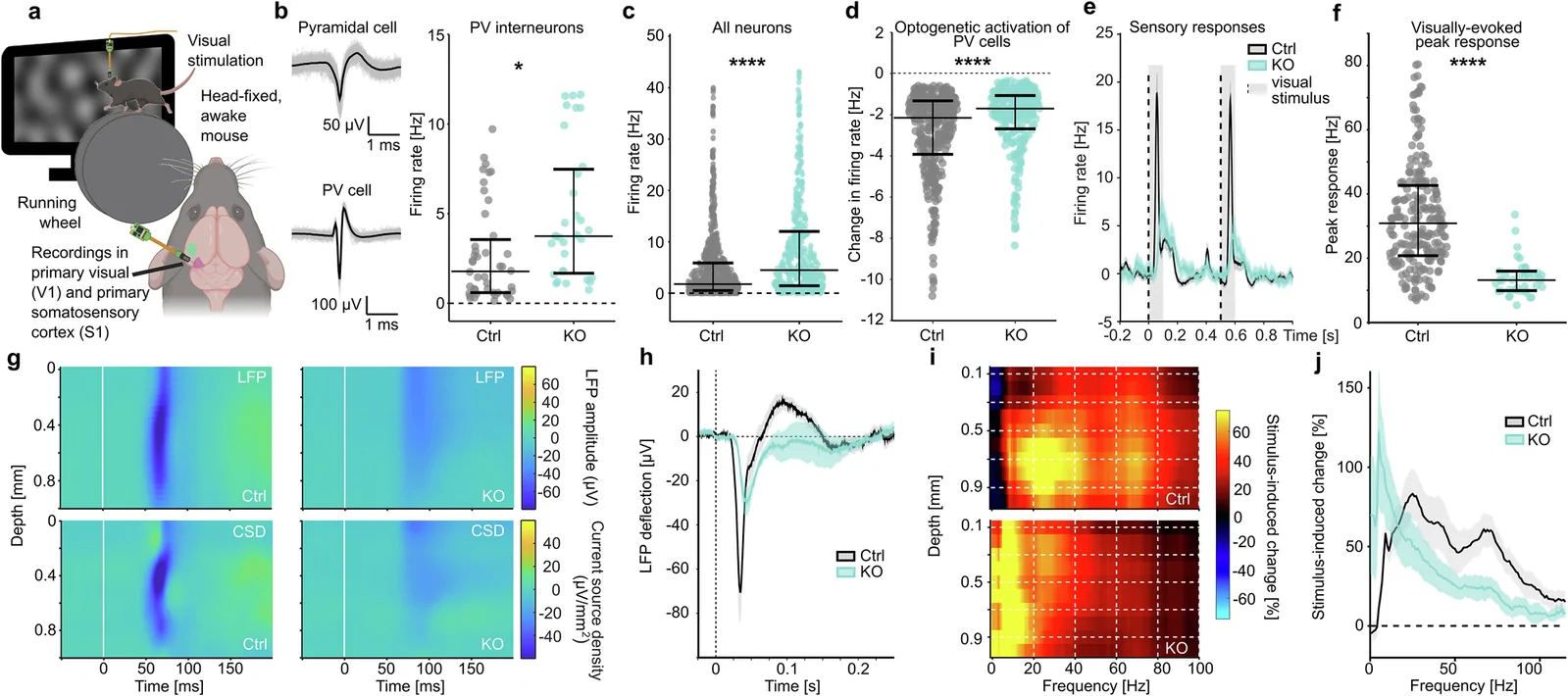
*Dnmt1*-knockout in PV interneurons alters spontaneous and induced neuronal activity. We performed electrophysiological recordings in the primary visual cortex (V1) of awake *Pvalb-Cre/tdTomato* control (Ctrl) and *Pvalb-Cre/tdTomato/Dnmt1 loxP*^2^ (KO) mice using Neuropixels probes (*n* = 8 recordings from 2 mice per genotype). **a** Schematic illustration of the experimental setup and recording sites. **b** Fast-spiking PV interneurons were identified via optotagging (see example spike waveforms, left; individual traces are shown in light gray, group average in black). Their spontaneous firing rates were significantly increased in KO mice (*n*_Ctrl_ = 54 and *n*_KO_ = 37 neurons; linear mixed-effects (LME) model: *p*_*(Genotype)*_ = 0.0174). **c** Spontaneous firing rates of V1 neurons were significantly higher in KO mice compared to control mice (*n*_Ctrl_ = 838 and *n*_KO_ = 448 neurons; LME model: *p*_*(Genotype)*_ = 6.69 × 10^−14^). **d** Relative change in the firing rate of V1 neurons inhibited by optogenetic activation of PV interneurons. PV-induced inhibition was significantly weaker in KO mice (*n*_Ctrl_ = 332 and *n*_KO_ = 238 neurons; LME model: *p*_*(Genotype)*_ = 5.66 × 10^−8^). **e** Average firing rates of V1 neurons in response to visual stimulation in control and knockout mice. Visual stimuli were 0.1-s-long, full-field presentations of low-frequency noise patterns, presented at time point 0 and after 0.5 seconds. Thick lines represent group means and shading shows the standard error of the mean (SEM) across neurons. **f** Peak response firing rate of V1 neurons after visual stimulation. KO mice had significantly weaker sensory responses compared to control mice (*n*_Ctrl_ = 293 and *n*_KO_ = 74 neurons; LME model: *p*_*(Genotype)*_ = 1.44 × 10^−12^). **g** Local field potentials (LFP, upper panels) and current source densities (CSD, lower panels) of control and *Dnmt1* KO mice across the whole depth of the cortex. White lines indicate visual stimulus onset. **h** LFPs in V1 in response to visual stimulation at time point 0, averaged across cortical layers. Thick lines indicate group means, and shading shows SEM across recordings. **(i, j)** KO mice displayed strongly diminished gamma oscillations (30–90 Hz) in comparison to control mice. **i** Relative change in spectral power compared to baseline activity. Visually evoked oscillations in control mice matched the gamma band (30–90 Hz), while in KO mice oscillations of much lower frequencies (<20 Hz) occurred. **j** Change in spectral power within the cortex is displayed as mean ± SEM. The direct comparison between control and *Dnmt1* KO mice revealed the shift towards lower-frequency oscillations in KO mice. **b**–**f** Display individual data points with median (center line) and interquartile range (upper and lower lines). Ctrl = control, CSD = current source density, KO = knockout, LFP = local field potential, PV = parvalbumin, V1 = primary visual cortex; * *p* < 0.05, ** *p* < 0.01, *** *p* < 0.001, **** *p* < 0.0001.

Building on a PV-specific conditional *Dnmt1* knockout mouse line^8,18^, we already demonstrated that DNMT1 regulates GABAergic transmission in PV interneurons through regulation of endocytosis-associated gene expression^8^. Given that cortical interneuron dysfunction and dysregulated *Dnmt1* expression have both been implicated in neuropsychiatric diseases^19^, we next examined the functional consequences of *Dnmt1* deletion in PV interneurons at the network level. To capture cortical network activity in vivo, we performed high-density Neuropixels recordings in awake mice, allowing us to simultaneously monitor the activity of large neural populations across cortical layers during both spontaneous activity and visual stimulation. *Pvalb-Cre/tdTomato/Dnmt1 loxP*^2^ knockout (KO) and *Pvalb-Cre/tdTomato* control mice were head-fixed on a running wheel and free to run at will (Fig. 1a), with two screens placed laterally to deliver visual stimuli.

To selectively identify and modulate PV interneuron activity, we used viral injections to induce Cre-dependent expression of the blue light-sensitive opsin Channelrhodopsin-2 (ChR2) in V1 PV interneurons. This allowed us to isolate PV spiking activity within the recorded neural population based on their short-latency responses to 10-ms-long blue light pulses (Fig. 1b). Spontaneous firing rates of PV interneurons in *Pvalb-Cre/Dnmt1* KO mice were elevated relative to controls, suggesting that DNMT1 is involved in restraining PV interneuron excitability and confirming our previous in vitro results^8^.

When considering all recorded cortical neurons, including excitatory cells, spontaneous firing rates were also increased in KO mice (Fig. 1c). Moreover, optogenetic activation of PV interneurons strongly inhibited cortical activity in controls but was markedly less effective in KO mice (Fig. 1d). This suggests that the inhibitory impact of PV interneurons on cortical networks was reduced despite their hyperactivity, consistent with homeostatic network adaptation.

Visual stimulation further revealed altered cortical responses in KO mice. Compared to controls, stimulus-evoked activity in V1 was weaker, temporally delayed, and less precise, with reduced stimulus specificity across cortical layers (Fig. 1a, e–h, Supplementary Fig. 1a–c). In line with earlier findings^9,20^, visual responses in control mice showed a well-defined laminar organization, with the earliest and strongest responses in layer 4 (0.3–0.5 mm) and subsequent activation of the superficial and infragranular layers, as evident in the local field potentials (LFPs; Fig. 1g, upper panels) and current source densities (CSD; Fig. 1g, lower panels). In contrast, excitatory responses across all cortical layers were reduced in KO mice, and the inhibitory rebound component typically associated with feedback inhibition was absent (Fig. 1h). These alterations underscore an impaired contribution of PV interneurons to cortical information processing in KO mice. Consistent with the weakened sensory responses in KO mice (Fig. 1e), we also found fewer stimulus-responsive neurons with a lower response specificity (Supplementary Fig. 1c).

To assess whether these impairments were specific to the visual cortex or generalize to other sensory modalities, we also recorded cortical activity in the primary somatosensory cortex (S1) during whisker stimulation. In controls, tactile input evoked robust and temporally precise responses, peaking in layer 5, followed by a characteristic inhibitory rebound reflecting feedback inhibition (Supplementary Fig. 1d, e). In contrast, *Dnmt1* KO mice showed markedly weaker and less temporally precise responses that were prolonged and lacked the inhibitory rebound that is typically mediated by PV interneurons (Supplementary Fig. 1d, e). Optogenetic activation of PV interneurons during tactile stimulation further confirmed their reduced efficacy: while PV activation strongly suppressed peak responses in controls, it had little effect in KO mice (Supplementary Fig. 1f). These findings therefore match our observations in V1 and indicate that DNMT1 loss in PV interneurons impairs their inhibitory contribution across primary cortical areas.

Next, we extended our recordings to examine higher-order cortical dynamics, focusing on the anterolateral motor cortex (ALM), a prefrontal region engaged in decision-making and sensory evidence accumulation. Because ALM is strongly recruited during goal-directed behaviors, we sought to determine whether DNMT1 loss in PV interneurons also affects higher-order cortical function and behavioral task performance. To exclude general learning deficits, we first examined spatial learning and memory by conducting a Morris water maze test. KO and control mice located the hidden platform with comparable accuracy and efficiency, indicating intact spatial learning capability (Supplementary Fig. 2a–d). We then trained mice on a visual evidence accumulation task, in which visual stimuli were concomitantly presented on both sides of the animals which had to correctly identify the side with the higher number of visual cues by licking a corresponding waterspout (Supplementary Fig. 2e). Beyond basic visual perception, this task requires temporal integration of sensory evidence and short-term memory, which are linked to parietal and frontal cortical activity^21^. KO mice acquired the task as efficiently as controls, with no differences in learning curves or absolute task performance (Supplementary Fig. 2f, g).

Electrophysiological recordings nevertheless revealed clear alterations in network activity. Similar to V1, ALM neurons in KO mice showed attenuated neural responses to visual stimulation (Supplementary Fig. 2h), and optogenetic activation of PV interneurons again confirmed their reduced inhibitory efficacy (Supplementary Fig. 2i). Importantly, optogenetic activation of PV interneurons impaired decision-making in both genotypes, demonstrating that ALM function remained essential for task performance (Supplementary Fig. 2j).

Together, these findings demonstrate that DNMT1 loss in PV interneurons reduces their inhibitory efficacy across cortical regions, spanning from primary sensory areas to prefrontal circuits critical for decision-making. However, despite these robust physiological alterations, basic perceptual, learning, and memory capacities were preserved, suggesting that compensatory network mechanisms can buffer the functional consequences of impaired PV interneuron control.

### DNMT1 deficiency in PV interneurons impairs gamma synchrony and induces depression-like behavior

PV interneurons are critically involved in the generation of cortical gamma oscillations^22,23^. Strikingly, alongside the altered firing patterns in V1 (Fig. 1g, h) *Dnmt1* KO mice also displayed severely reduced visually evoked gamma oscillations (Fig. 1i, j). Because gamma synchrony depends on the precise timing of PV interneuron output, our findings suggest that DNMT1 is also required to maintain the rhythmic inhibitory drive underlying fast cortical coordination. Importantly, impaired gamma oscillations are a well-documented hallmark of neuropsychiatric disorders, including schizophrenia, bipolar disorder, and mood disorders^24^. In patients with depression, reduced gamma power has been associated with cognitive inflexibility, impaired attention, and diminished affective regulation, while animal models consistently link PV dysfunction and gamma desynchronization to depression-like behaviors^23,25,26^.

**Fig. 2:**
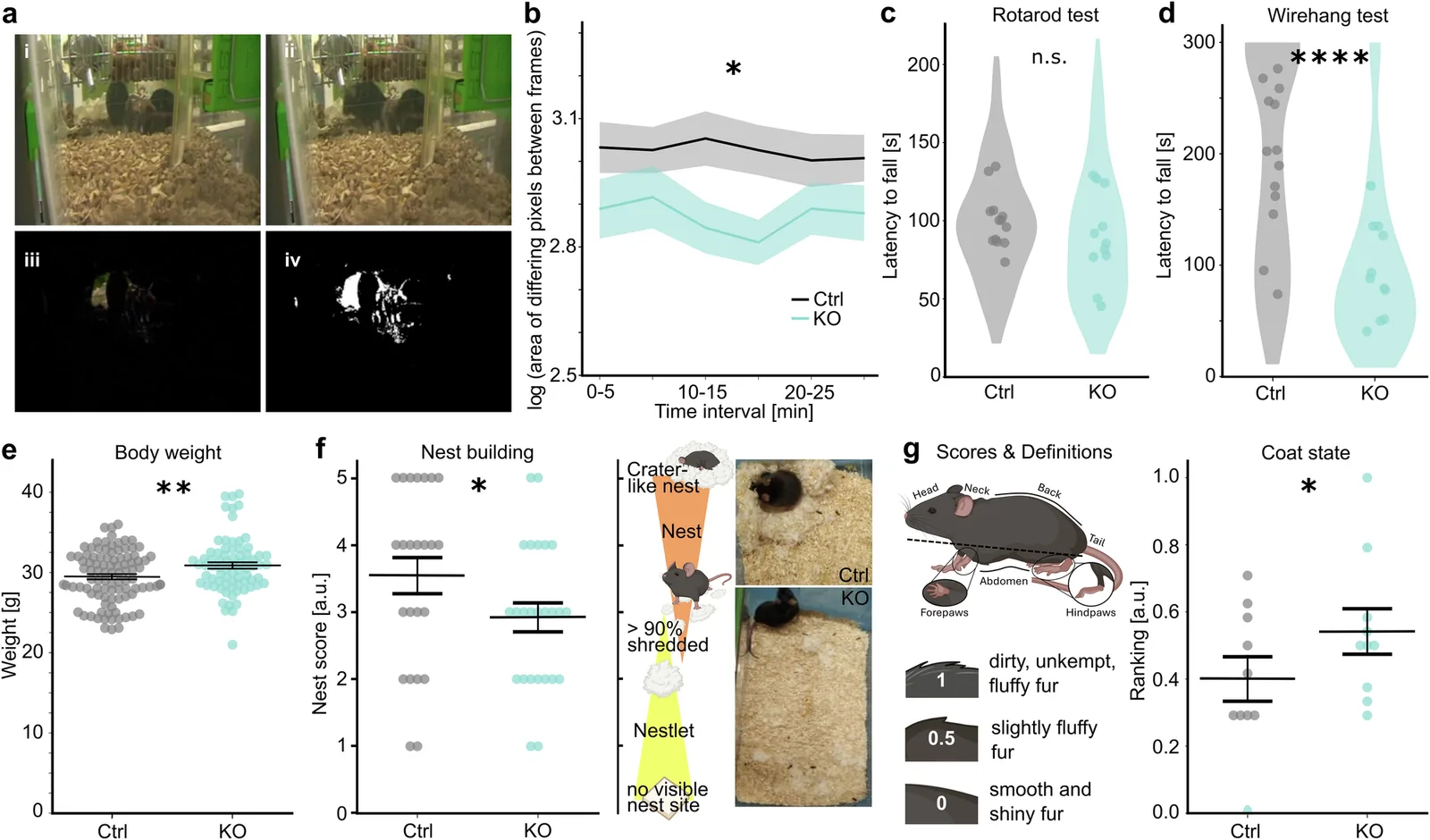
*Pvalb-Cre/tdTomato/Dnmt1 loxP*^2^ (KO) mice display apathetic behavior. **a**, **b** Spontaneous activity within the home-cage was consistently diminished in KO mice compared to *Pvalb-Cre/tdTomato* (control/Ctrl) mice (*n* = 6 mice per genotype). **a** Mice were monitored in their home cages to evaluate their spontaneous, undisturbed behavior. By comparing every fifth frame from the resulting videos (i, ii), the change in pixel values between frames (iii) was binarized (iv) to calculate the change in position of the contained animals (images iii–iv were created using ImageJ). **b** Comparison of multiple 30-min time intervals revealed consistently lower activity levels of KO mice (mean ± SEM; LME model: *p*_*(Genotype)*_ = 0.034). **c** Average latency to fall from a rotating rod (‘Rotarod’) with increasing velocity showed no apparent motor deficits in knockout mice (*n*_(Control)_ = 13 mice, *n*_(Knockout)_ = 11 mice; LME model: *p*_*(Genotype)*_ = 0.156). **d** A wire hang test showed a significantly reduced latency to fall in KO mice, which can indicate a difference in motor strength but is highly dependent on the animals’ body weight (*n*_(Control)_ = 13 mice, *n*_(Knockout)_ = 11 mice; LME model: *p*_*(Genotype)*_ < 0.0001). **e** Body weights were elevated for KO mice compared to controls (*n*_(Control)_ = 43 mice, *n*_(Knockout)_ = 33 mice; One-way ANOVA: *p* = 0.0068). **f** Nest-building capacities were significantly decreased in KO (*n* = 25 mice) versus control mice (*n* = 24 mice; Generalized estimating equation model: *p*_*(Genotype)*_ = 0.006). Nest quality was assessed using an ordinal scale from 1–5 as visualized by the schematic in the middle. As the representative images (right side) show, knockout mice were shredding the provided material but failed to assemble visible nest sites. **g** Assessment of the animals’ coat state as indicated in the illustration on the bottom right showed significantly higher rankings of the knockout mice, indicating worsened fur care (*n* = 12 mice per genotype; Mann-Whitney *U* test: *p* = 0.026). In (**c**, **d**) the violins display the distribution of data obtained from three trials per animal, while individual data points represent the mean value per mouse. **e**–**g** Display individual data points with median (centre line) and interquartile range (upper and lower lines). Ctrl = control, KO =  knockout, LME = linear mixed-effects; * *p* < 0.05, ** *p* < 0.01, *** *p* < 0.001, **** *p* < 0.0001.

Notably, bulk RNA sequencing of FACS-enriched PV cells generated in a previous study^8^ revealed that differentially expressed genes (DEGs) in *Dnmt1* KO mice are significantly enriched in transcriptional signatures from diverse mutant mouse models, including lines with and without depression- and anxiety-like phenotypes (Supplementary Tab. 1, 2)^27–34^, indicating shared molecular stress- and plasticity-related programs rather than a disorder-specific signature. Given this broader convergence on stress- and plasticity-related pathways, we next asked whether DNMT1 loss in PV interneurons might nonetheless be sufficient to trigger mood disorder-related behaviors. Spontaneous home-cage activity provides a sensitive measure of innate motivation and has been widely used to detect depression- and apathy-like phenotypes in rodents^35^. Monitoring home-cage behavior revealed consistently reduced movements in KO mice (Fig. 2a, b), a change not explained by visual deficits (Supplementary Fig. 2) or motor deficits. KO mice performed normally in rotarod (Fig. 2c and Supplementary Fig. 3a) and ladder rung tests^18^, but showed a shorter latency to fall in the wire hang test (Fig. 2d and Supplementary Fig. 3b), which coincided with significantly increased body weight (Fig. 2e). Increased body weight is often comorbid with depressive symptoms^36^, and the weight gain of KO mice could therefore reflect reduced physical activity and energy expenditure. In support of this, food intake was unchanged (Supplementary Fig. 3c–e), and we found no indications for altered appetite regulation (Supplementary Fig. 3f, 4, 5). Body weight regulation is controlled by circulating metabolic hormones acting on hypothalamic circuits^37,38^. Key circulating factors involved in appetite regulation, energy homeostasis, and metabolic signaling include ghrelin, leptin, insulin, PYY, GLP-1, glucagon, and TNFα^39,40^. We did not detect significant changes in any of these hormones in KO mice except for elevated leptin levels (Supplementary Fig. 3f); this increase in leptin levels likely represents a secondary effect of the higher body weight rather than altered endocrine regulation, as leptin is secreted by adipose tissue and promotes satiety^41,42^.

Among the hormonally targeted hypothalamic circuits, the arcuate nucleus is a key integrative center for energy homeostasis^43^. Two major and functionally opposing neuronal populations in this region are agouti-related peptide (AgRP)-expressing neurons, which promote feeding (orexigenic), and proopiomelanocortin (POMC)-expressing neurons, which suppress food intake (anorexigenic). These populations integrate peripheral hormonal signals to coordinate appetite, energy expenditure, and body weight. Immunostaining of the arcuate nucleus confirmed that PV-positive cells are largely distinct from the major neuronal populations regulating feeding, showing no overlap with POMC neurons and only partial overlap with AgRP neurons (Supplementary Fig. 4, 5)^43^. Therefore, while we cannot entirely exclude hypothalamic defects in KO mice, it seems unlikely that the observed behavioral phenotype is driven by changes in feeding circuitry. Together, the increased weight and diminished motor activity in KO mice are consistent with lower energy expenditure and diminished innate motivation, compatible with an apathy-like state^44^.

Additional behavioral assays further supported an apathetic and anhedonic phenotype of *Dnmt1* KO mice. Nest-building, an innate behavior essential for heat conservation and shelter, is a sensitive readout of apathetic states in rodents^45^. Using a 1–5 ordinal scale, we found significantly lower nest scores in *Dnmt1* KO mice (Fig. 2f), typically shredding but failing to assemble the nesting material within 16 hours. Assessment of coat state, a marker of self-care and motivation^46,47^, also revealed systematic neglect in *Dnmt1* KO mice, with significantly higher cumulative scores compared to controls indicating a deterioration of their overall grooming and coat care (Fig. 2g).

**Fig. 3:**
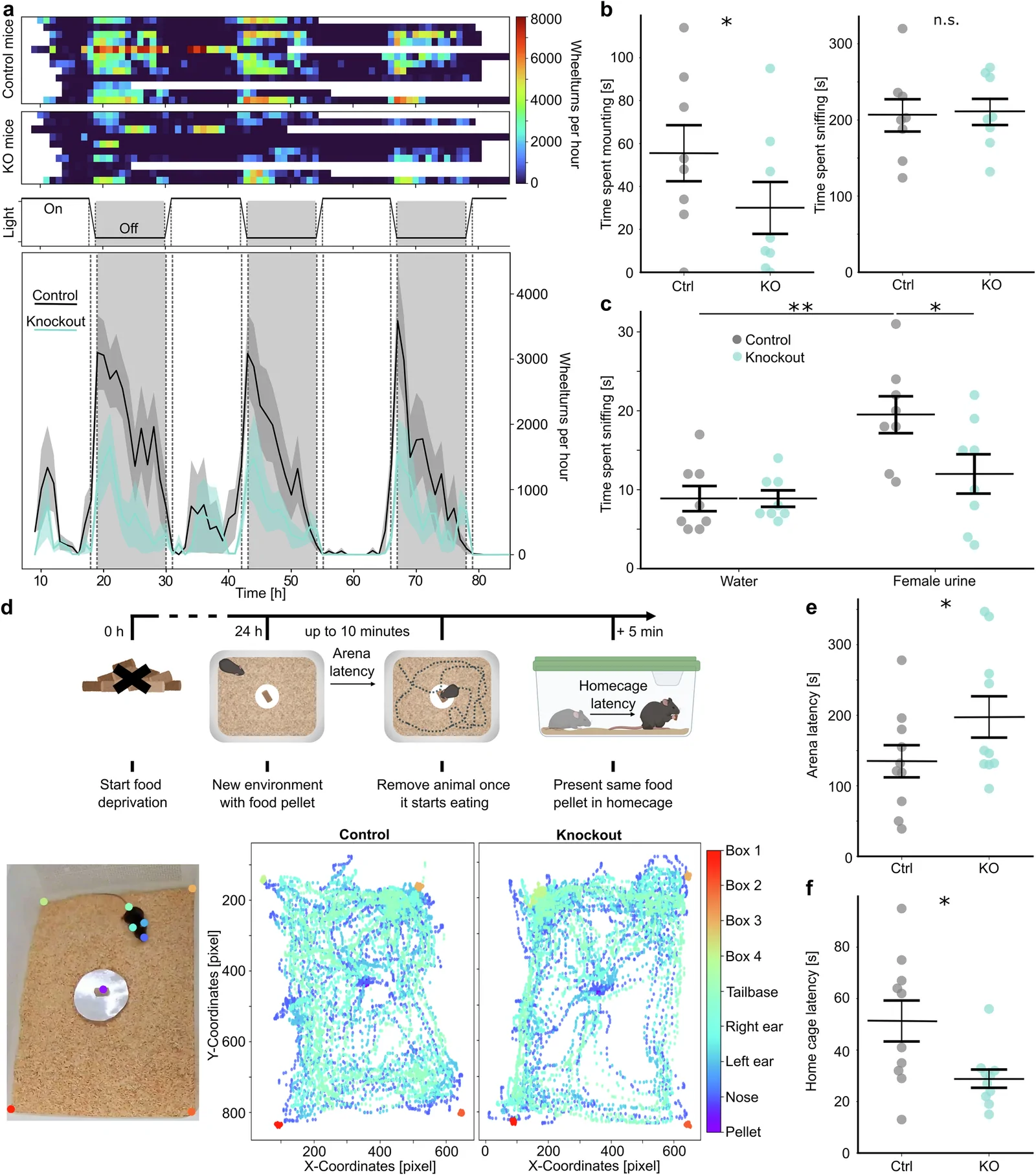
*Pvalb-Cre/tdTomato/Dnmt1 loxP*^2^ (KO) mice display traits of an anhedonic, anxious phenotype. **a** Voluntary running on a wheel provided within the home cage was strongly reduced in KO (*n* = 9) versus *Pvalb-Cre/tdTomato* (control/Ctrl) mice (*n* = 11). Running wheels were provided in the home cages of single-housed mice for up to three nights. Almost all mice displayed fluctuating utilization of the running wheel (upper heat maps) based on the daytime (middle graph), but in general, KO mice were running less frequently than controls. The overall reduced activity of knockout mice is discernible in the mean running speed averaged per genotype (lower graph: thick lines show group means, shading represents SEMs; LME model: *p*_*(Genotype)*_ = 0.001, *p*_*(Genotype x light phase)*_ < 0.001; post-hoc testing of genotype/light phase-interaction: *p*_*(Genotype x light)*_ = 0.37, *p*_*(Genotype x dark)*_ = 0.0093). **b** Evaluating the interaction of male KO and control mice (*n* = 8 mice per genotype) with female C57BL/6J mice in a 10-min time frame revealed that KO males spent significantly less time trying to mount the females (left graph, two-sided Student’s *t*-test: *p* = 0.019), while they spent similar times sniffing their conspecifics (right graph, two-sided Student’s *t*-test: *p* = 0.44). **c** Presentation of pooled urine from female C57BL/6J mice significantly increased time spent sniffing in control but not in KO mice (*n* = 8 mice per genotype; linear mixed-effects model: *p*_*(Genotype x stimulant type)*_ = 0.048, Tukey post-hoc test: *p*_*(Ctrl: water vs urine)*_ = 0.0017, *p*_*(KO: water vs. urine)*_ = 0.25, *p*_*(Water: Ctrl vs KO)*_ = 1.0, *p*_*(Urine: Ctrl vs KO)*_ = 0.0413). **d**–**f** Conditional *Dnmt1* deletion in PV cells induces anxiety in a novelty-suppressed feeding (NSF) test (*n* = 10 per genotype). **d** Upper row: Scheme and schedule of the NSF test. Mice were food deprived for 24 h before being transferred into an arena with new bedding and a centrally attached food pellet of known weight. Once the animals started chewing the presented food, their latency was recorded, and they were immediately removed to their home cage. Subsequently, the pellet from the arena was presented in the home cage, and the animals’ latency to approach and consume it was recorded. Videos taken during the NSF test were labeled using DeepLabCut to evaluate the animals’ behavior until they approached the food pellet (lower graphs). **e** KO mice displayed a significantly increased latency to approach the presented food pellet in the arena compared to controls, suggesting increased levels of anxiety (two-sided Student’s *t*-test: *p* = 0.0184). **f** In their home cages, KO mice approached the same food pellet significantly faster than control mice (two-sided Student’s *t*-test: *p* = 0.0193). **b**, **c**, **e**, **f** Display individual data points with median (centre line) and interquartile range (upper and lower lines). Ctrl = control, KO = knockout; * *p* < 0.05, ** *p* < 0.01, *** *p* < 0.001, **** *p* < 0.0001.

Consistent with lower self-motivated activity, voluntary wheel running was strongly reduced (Fig. 3a), particularly during the dark phase, suggesting diminished motivation for rewarding behaviors^48,49^. KO males also displayed reduced sexual motivation, mounting females less frequently (Fig. 3b), and showing no attraction to female urine (Fig. 3c), although general social interaction remained intact (Fig. 3b). Taken together, *Dnmt1*-deficient mice show a cluster of behavioral traits, including apathy, anhedonia, and reduced spontaneous activity, that collectively are consistent with a dysphoric-like state with features reminiscent of depressive symptoms.

### *Dnmt1* deletion in PV interneurons leads to increased anxiety in mice

To assess whether *Dnmt1* KO mice also display altered anxiety levels, we performed the novelty-suppressed feeding (NSF) test, which measures the conflict between food-seeking motivation and anxiety in a novel environment (Fig. 3d). After 24 hours of food deprivation, both genotypes showed similar weight loss (Supplementary Fig. 6a), implying a similarly strong motivation to obtain food.

Nevertheless, *Dnmt1* KO mice took significantly longer to approach and consume a food pellet centrally placed in an open arena (Fig. 3e) and foraged closer to the walls of the arena, reflected by an increased mean distance to the pellet (Fig. 3d). To confirm that this effect reflected anxiety rather than altered motivation, mice were transferred to their home cages immediately after they started biting the pellet. With the food deprivation still serving as an active motivator in this familiar environment, KO mice approached the pellet faster than controls (Fig. 3f) and consumed equal amounts within 5 minutes (Supplementary Fig. 6b). These results demonstrate that DNMT1 loss in PV interneurons increases novelty-induced anxiety, further complementing the depression-like phenotype of KO mice.

### DNMT1 loss in PV interneurons triggers cortex-wide transcriptional and network-level disruption

The combined electrophysiological and behavioral results show that DNMT1 loss in PV interneurons disrupts inhibitory control, weakens gamma synchrony, and gives rise to a spectrum of depression- and anxiety-related behaviors. Notably, DNMT1 loss not only increased firing of PV interneurons but also elevated spontaneous activity across the broader neuronal population, consistent with an adaptive network response to altered inhibition. These findings highlight the cell-autonomous role of DNMT1 in PV interneurons but also the need to understand how such local dysfunctions reverberate through cortical circuits. To capture these potential non-cell-autonomous effects at the transcriptomic scale and across all major cortical cell types, we performed single-nucleus RNA sequencing on cortical tissue from adult *Dnmt1* KO and control mice. This approach enabled us to assess at a systems-level how DNMT1-dependent dysfunction in PV interneurons destabilizes cortical networks.

**Fig. 4:**
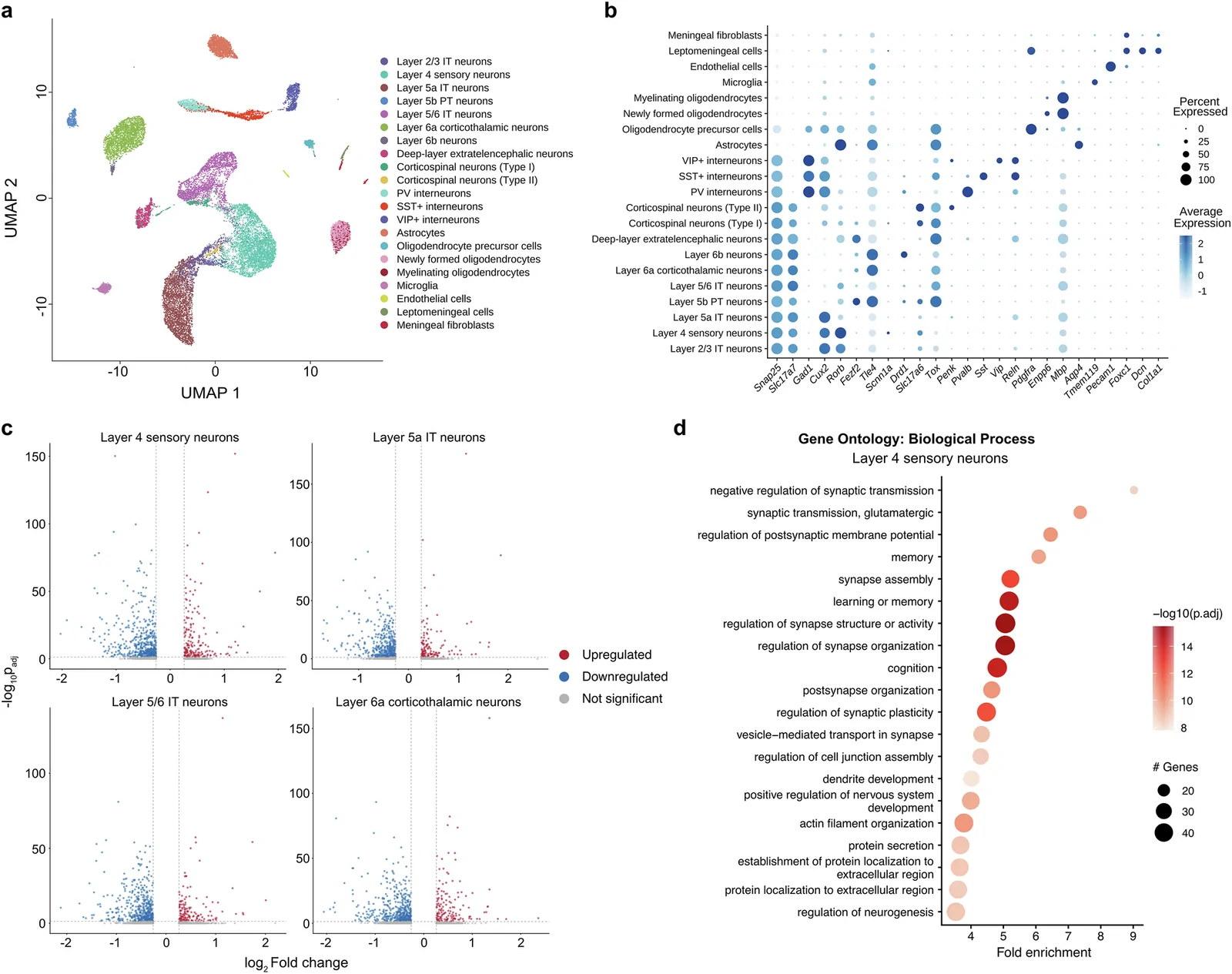
Single-nucleus RNA sequencing reveals transcriptomic shifts in excitatory neurons in *Pvalb‑Cre/tdTomato/Dnmt1 loxP*^2^ (KO) mice. **a** Uniform manifold approximation and projection (UMAP) embedding reveals the expected cellular composition of the neocortex. **b** Dot plot of marker genes across the identified clusters. **c** Volcano plots illustrate the extent of upregulated and downregulated genes in excitatory neurons from layers 4–6 of KO mice (|log_2_FC| ≥ 0.26, adjusted *p* value < 0.05). **d** Gene ontology analysis of differentially expressed genes (DEGs) in the layer 4 sensory neurons reveals an enrichment for biological processes related to synaptic transmission, organization, and activity.

Unsupervised clustering of single nuclei isolated from the cortical tissue of adult *Dnmt1* KO and control mice revealed the expected diversity of neuronal and glial cell types, including excitatory neurons, PV and non-PV interneurons, oligodendrocytes, astrocytes, and microglia (Fig. 4a, b and Supplementary Fig. 7a). Interestingly, although *Dnmt1* deletion was restricted to PV interneurons, two-sample proportion tests and neighborhood-based differential abundance analysis using Milo^50^ identified significant changes in a limited set of cortical populations (Supplementary Data File 1 and Supplementary Fig. 7b–d). Layer 4 (L4) sensory neurons were modestly but significantly enriched in the KO cortex, while layer 5/6 (L5/6) intratelencephalic (IT) neurons and astrocytes were depleted. In line with the modest reduction in astrocyte representation detected by snRNA-seq, immunostaining for GFAP revealed significantly lower fluorescence intensity in the somatosensory cortex of *Dnmt1* KO mice than in the controls (Supplementary Fig. 7e, f), supporting an astrocyte-associated alteration in the KO cortex. Notably, two populations of corticospinal-like neurons, defined by the expression of *Tox* and *Slc17a6* and the absence of *Tle4* and *Cux2* (Fig. 4b), were markedly more in the KO cortex, with Milo confirming enrichment across 92–100% of transcriptional neighborhoods assigned to the corticospinal-like populations (Supplementary Fig. 7c). A reduction in layer 5 neurons is a well-described technical artifact of snRNA-seq in cortical tissue, attributed to slower fixation, RNA degradation in deeper cortical layers, mechanical fragility of large pyramidal nuclei with low nucleus-to-cytoplasm ratios, and inefficient recovery of long, chromatin-associated transcripts characteristic of layer 5 pyramidal cells^51–54^. Consequently, apparent shifts in excitatory subclass proportions in split-pool snRNA-seq datasets should be interpreted with caution. The pronounced enrichment of corticospinal-like neuron populations, however, is less likely due to this technical factor, as both proportion tests and Milo consistently show increased abundance in the KO cortex. We therefore interpret this as a biologically plausible, candidate non-cell-autonomous shift in transcriptional states. These proportional shifts are, nonetheless, identified within the snRNA-seq dataset and should therefore be viewed as hypothesis-generating, as no orthogonal validation (e.g., FISH) has been performed.

We further detected broad transcriptional alterations across multiple cell types between samples from control and KO mice, with the most extensive changes in excitatory neurons of layers 4–6 (Fig. 4c). Notably, the differentially expressed genes of excitatory neurons in layer 4, the principal thalamorecipient cortical layer heavily innervated by PV interneurons^55^, were enriched for gene ontology (GO) terms related to synaptic transmission, structure, and organization, consistent with altered input-layer gating due to disrupted inhibitory control (Fig. 4d). Since DNMT1 loss was selective to PV interneurons, these alterations across excitatory cell types likely reflect secondary, non-cell-autonomous effects.

Beyond cluster-specific changes, two genes were downregulated across the broadest range of cell types: *Fkbp5*, a negative regulator of glucocorticoid receptor sensitivity linked to stress-related psychiatric disorders^56,57^, and *Zbtb16* (PLZF), a glucocorticoid-inducible zinc-finger transcription factor recently implicated in brain stress responses^58,59^, were suppressed in 12 and 14 of 21 cell clusters, respectively (Supplementary Data File 2). Their broad suppression across excitatory neurons, interneurons, astrocytes, and oligodendroglia indicates a cortex-wide disruption of stress-adaptive transcriptional programs, which aligns with the depression-like behavioral phenotype in *Dnmt1* KO mice. Excitatory neurons further showed consistent downregulation of activity-dependent immediate early genes including *Arc*, *Fos*, and *Nr4a1–3*^60^, consistent with reduced synchronous, stimulus-driven activity despite elevated tonic firing rates. Importantly, non-neuronal and non-glial cell populations, i.e., endothelial cells, leptomeningeal cells, and meningeal fibroblasts, showed no significant DEGs, confirming that the transcriptional response is cell-type specific.

### Chromatin-level regulatory network analysis links transcriptional rewiring to eRegulon activity

**Fig. 5:**
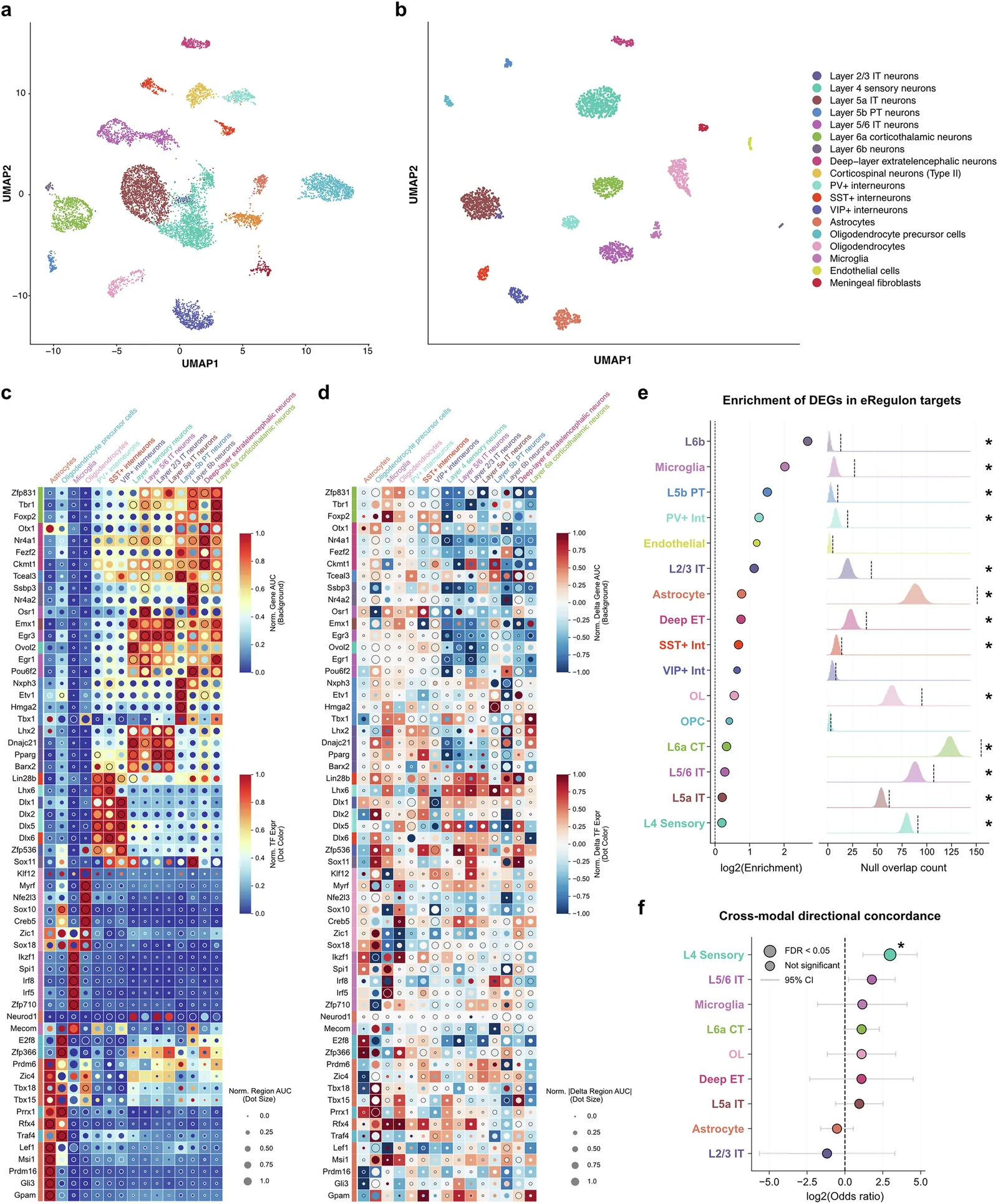
eRegulon-based gene regulatory network analysis of transcription factor target-gene activity and chromatin accessibility across cortical cell types in *Pvalb-Cre/tdTomato/Dnmt1* loxP2 (KO) mice. **a** UMAP embedding of all profiled nuclei by snATAC-seq, colored by cell type identity assigned through RNA-based anchoring/label transfer. **b** UMAP embedding of metacells based on eRegulon activity (gene-based AUC scores computed by SCENIC+), confirming that regulon activity scores preserve cell-type-resolved structure at the metacell level. **c** Heatmap-dotplot summarizing eRegulon activity across cell types. Background tile color denotes min-max normalized gene-based eRegulon AUC (regulon target-gene activity) per cell type; dot color denotes min-max normalized TF expression; dot size denotes min-max normalized region-based eRegulon AUC (chromatin accessibility at regulon target regions). Rows (TF-eRegulon pairs) and columns (cell types) are ordered by hierarchical clustering (Ward linkage, Euclidean distance). **d** Heatmap-dotplot highlighting genotype-associated shifts in regulon activity, TF expression, and chromatin accessibility per cell type (KO-minus-Ctrl). **e** Enrichment of DEGs (KO vs Ctrl) among eRegulon target genes, per cell type, tested via one-sided permutation (10,000 permutations) with Benjamini-Hochberg FDR correction. Left: log2(Enrichment). Right: null overlap distributions per cell type. **f** Cross-modal directional concordance between ATAC and RNA changes (KO vs Ctrl) among eRegulon target genes, restricted to genes with a stable ATAC call and significant DEG call (minimum 15 genes per test). Points represent the log_2_ odds ratio and error bars represent 95% confidence intervals from Fisher’s exact test (two-sided); significance assessed via one-sided permutation test (10,000 permutations) with Benjamini-Hochberg FDR correction. * FDR < 0.05.

Given the breadth of the transcriptomic changes observed by snRNA-seq, we next asked whether these shifts were underpinned by alterations in chromatin accessibility and transcription factor (TF)-driven regulatory programs. To resolve this chromatin-level architecture, we generated single-nucleus ATAC-seq profiles from matched cortical tissue in an independent cohort of *Dnmt1* KO and control mice, and assigned cell type identity to all profiled nuclei via RNA-based anchoring and label transfer (Fig. 5a). We then performed eRegulon-based gene regulatory network (GRN) inference using SCENIC + , which jointly models TF expression, chromatin accessibility at TF binding motifs, and target gene expression into unified regulons. UMAP embedding of metacells based on eRegulon activity (gene-based AUC) confirmed that regulon activity scores preserve cell-type-resolved structure at the metacell level, validating the robustness of this approach (Fig. 5b).

Hierarchical clustering of TF-eRegulon pairs and cell types recovered coherent, cell-type-specific regulatory modules consistent with known lineage-determining transcription factors (Fig. 5c). We then computed genotype-associated shifts in this same tripartite signature (KO-minus-Ctrl) per cell type (Fig. 5d). Our analysis revealed pronounced, cell-type-specific regulon rewiring, including instances of coordinated shifts across all three modalities as well as modality-discordant shifts, where chromatin accessibility and TF expression changed in the opposite direction of target-gene output. Consistent with the hierarchy observed in our snRNA-seq DEGs, regulon activity changes were strongest in excitatory neurons across layers 4–6, microglia, and oligodendrocytes, whereas OPCs showed comparatively modest shifts (Fig. 5d). Notably, several regulons associated with excitatory and stress-responsive transcriptional programs, including *Etv1*, *Egr* family members, and *Nr4a* factors^61–63^, exhibited coordinated changes in target-gene AUC and region accessibility in deep-layer and corticothalamic neurons, mirroring the layer-specific DEG patterns and stress-gene suppression described above.

To test whether this regulon-level rewiring was functionally coupled to the transcriptomic phenotype, we assessed whether genotype-associated DEGs (KO vs Ctrl) were enriched among eRegulon target genes per cell type (Fig. 5e). The large majority of cell types showed significant enrichment, indicating that the DEGs identified in snRNA-seq are not randomly distributed across the genome but are preferentially concentrated within the gene regulatory networks inferred from chromatin accessibility, supporting a mechanistic link between the eRegulon rewiring and the transcriptomic phenotype.

Enrichment alone, however, does not establish the direction of the relationship between chromatin state and transcription. We therefore tested cross-modal directional concordance, i.e., whether genes with increased regulon-target chromatin accessibility in the KO (KO-open) also showed increased RNA expression (RNA-up), and conversely, whether KO-closed regions corresponded to RNA-down genes. This analysis was restricted to eRegulon target genes with both a stable ATAC call and a significant DEG call, with a minimum of 15 genes per test. L4 sensory neurons showed the strongest, and the only FDR-significant, concordance in the dataset, for both the KO-open/RNA-up direction and the KO-closed/RNA-down direction (Fig. 5f). No other cell type reached significance after FDR correction, indicating no directional coupling between chromatin and transcriptional change in this population. This selective, chromatin-anchored concordance in L4 sensory neurons is particularly notable given that this layer is densely innervated by PV interneurons^55^ and was previously identified as the population with a strong DEG enrichment for synaptic organization pathways (Fig. 4d). Together, these results suggest that L4 sensory neurons uniquely undergo a genuinely chromatin-driven transcriptional response to PV interneuron *Dnmt1* loss, rather than a downstream transcriptional consequence uncoupled from local chromatin remodeling.

These ATAC-based eRegulon analyses revealed a selective, chromatin-anchored transcriptional reprogramming in L4 sensory neurons, particularly affecting synapse-related gene programs, suggesting that DNMT1-dependent PV dysfunction reshapes not only local gene regulatory networks but also the connectivity with neighboring cell types. Building on this, we next asked how these cell-intrinsic regulatory changes translate into altered intercellular communication between PV interneurons and their surrounding neuronal and glial partners.

### Altered PV–oligodendrocyte communication suggests a reorganized perisomatic glial niche

To systematically map how the increased GABAergic transmission in *Dnmt1* KO mice (Fig. 1b) propagates through the cortical network, we applied CellChat, a cell-cell communication analysis tool that infers pairwise communication probability across populations from ligand-receptor mRNA co-expression. This allowed us to predict how communication networks linking PV interneurons and surrounding neuronal and glial populations are altered by PV-specific depletion of *Dnmt1*.

**Fig. 6:**
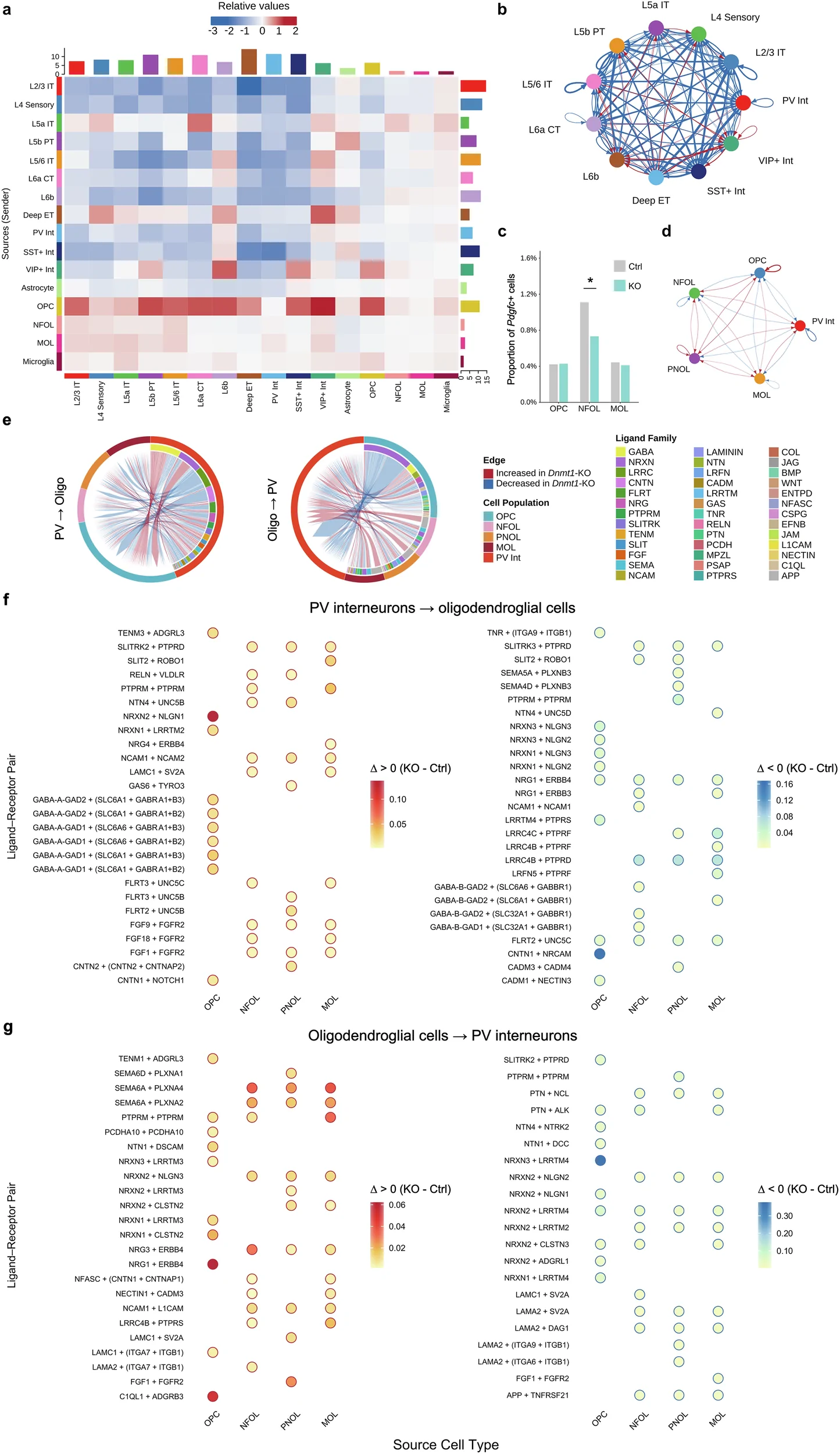
DNMT1 deficiency in PV interneurons reshapes intercellular communication and alters ligand–receptor interactions crucial for perineuronal nets (PNNs) in *Pvalb‑Cre/tdTomato/Dnmt1 loxP*^2^ (KO) mice. **a** CellChat-based network mapping demonstrates altered interaction weights among neuronal and glial cell clusters in the KO cortex. The senders, i.e., the clusters from which the signaling event is initiated, are listed on the y-axis, while the clusters receiving the signal are found in the x-axis. **b** The circular network diagram depicts altered connectivity patterns between the neuronal populations in KO mice. Increased and decreased communication probabilities in KO mice are depicted in red and blue, respectively. **c** The *Pdgfc*-expressing population within newly formed oligodendrocytes, representing the perineuronal oligodendrocytes, is significantly reduced in the KO cortex (two-sample proportion test, *p*_*adj*_ = 0.015). **d** The circular network diagram illustrates differential interactions between oligodendroglial cells and PV interneurons in KO mice. Increased and decreased communication probabilities in KO mice are depicted in red and blue, respectively. **e** Chord diagrams depict the altered interactions grouped by ligand/receptor family, separated based on directionality. The bubble plots depict the top 10 most severely altered significant L-R pairs per oligodendroglial cell type, ranked by absolute difference in communication probability, for (**f**) outgoing signaling from PV interneurons to oligodendroglial cells and (**g**) incoming signaling from oligodendroglial cells to PV interneurons (permutation test, *p* < 0.05). The top-ranking pairs are predominantly implicated in the stabilization of PNNs, the perisomatic niche, and cell–cell interactions. Increased and decreased communication probabilities are depicted on a yellow–red and a yellow–blue gradient, respectively. CT: Corticothalamic. ET: Extratelencephalic. IT: Intratelencephalic. OPC: Oligodendrocyte progenitor cells. MOL: Myelinating oligodendrocytes. NFOL: Newly formed oligodendrocytes. PNOL: Perineuronal oligodendrocytes. PT: Pyramidal tract.

Analysis of the CellChat-based interaction weight matrix revealed a widespread decrease in predicted pairwise communication probabilities between neuronal populations in the *Dnmt1* KO cortex: 82% of all pairwise interactions showed decreased weights, with the strongest net reductions in total outgoing communication probabilities observed for layer 2/3 (L2/3) IT, L4 sensory, somatostatinergic (SST + ), and L5/6 IT neurons (Fig. 6a). This broad pattern of reduced communication probability is consistent with the electrophysiological finding of temporally imprecise stimulus-evoked responses and reduced network synchrony, raising the possibility that DNMT1 loss in PV interneurons is associated with disrupted cortical communication beyond the inhibitory compartment.

Within this network-wide context, predicted communication probability between PV interneurons and other neuronal subtypes showed a particularly pronounced redistribution. Outgoing probability scores from PV cells were decreased toward all excitatory targets, with the largest reductions directed at deep-layer populations, while the decrease toward L4 sensory neurons was comparatively modest (Fig. 6a, b), suggesting a disproportionate loss of inhibitory control over subcortical output circuits relative to sensory input-layer gating. Conversely, incoming signaling scores to PV cells were broadly reduced across all layers, most prominently from SST+ interneurons, L2/3 IT neurons, and L5/6 IT neurons. The predicted reduction in SST + -to-PV signaling is noteworthy, as SST-PV connectivity normally contributes to the coordination of inhibitory interneuron ensembles and the stabilization of gamma oscillations^64^. Together, these predicted decreases in both excitatory and inhibitory input probability converging onto PV cells, combined with reduced PV output toward deep-layer circuits (Fig. 6a, b), suggest a potential decoupling of PV interneurons from their canonical network partners, which may contribute to the reduced inhibitory efficacy despite PV hyperactivity.

**Fig. 7:**
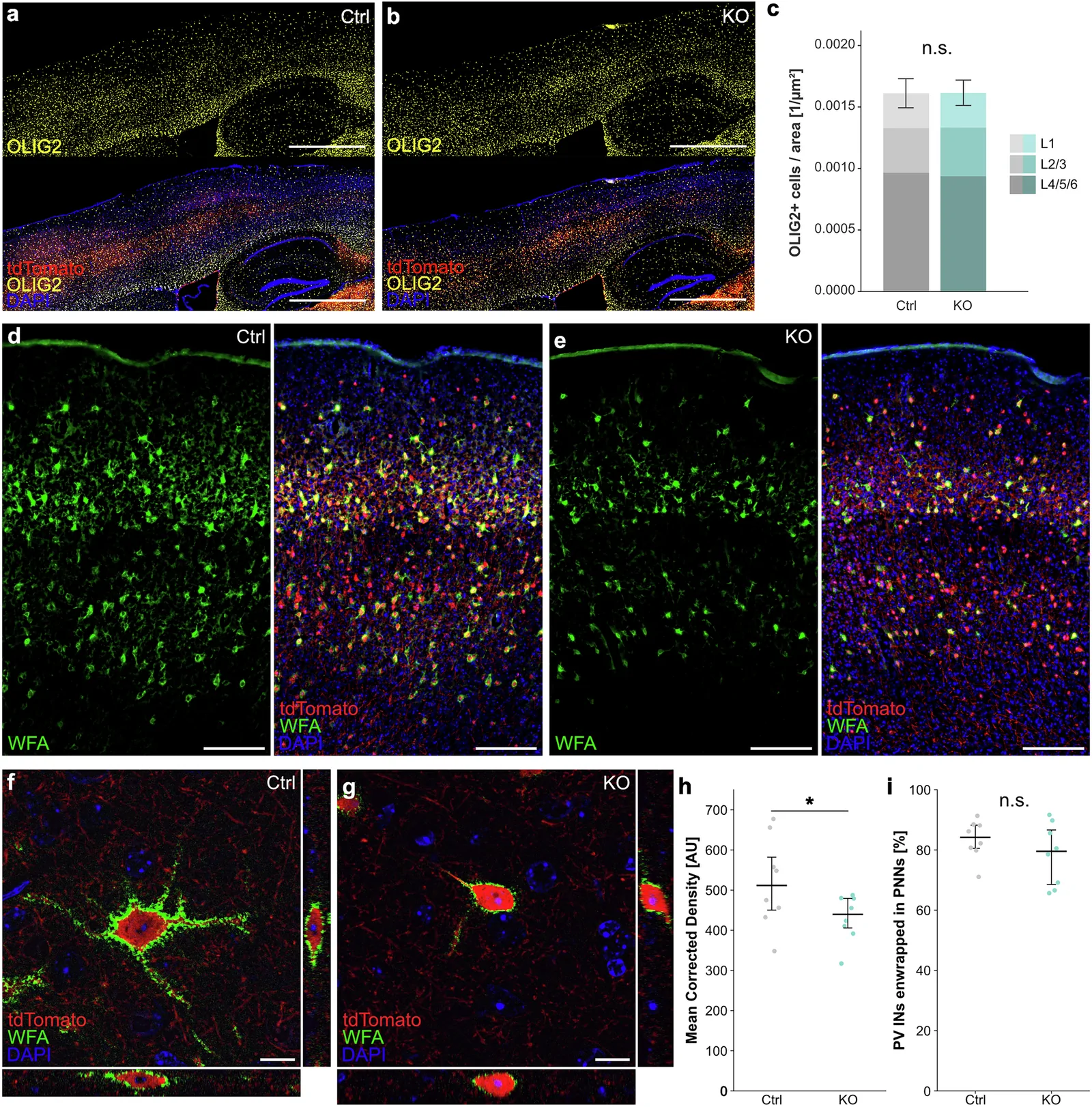
Loss of DNMT1 in PV interneurons disrupts perineuronal nets (PNNs) in the neocortex of *Pvalb‑Cre/tdTomato/Dnmt1 loxP*^2^ (KO) mice without impacting the total number of oligodendroglial cells. Representative microphotographs of the neocortex in parasagittal brain sections of *Pvalb-Cre/tdTomato* (Ctrl; **a**) and *Pvalb‑Cre/tdTomato/Dnmt1 loxP*^2^ (KO, **b**) mice stained for oligodendroglial cells with an anti-OLIG2 antibody. Scale bars: 1000 µm. **c** Quantification of OLIG2+ cell density revealed that the number of OLIG2-positive cells is unchanged in *Dnmt1* KO mice (bar heights show the group means with upper and lower lines indicating standard deviation and different shadings representing mean values for separate cortical layers; *n* = 15 slices per genotype from *N* = 5 brains/genotype; Linear mixed model: *p* = 0.867). Wisteria floribunda agglutinin (WFA) labeling of perineuronal nets (PNNs) in the primary somatosensory cortex (S1), shown in coronal sections from control (**d**) and *Dnmt1* KO (**e**) mice. Scale bars: 200 µm. **f**, **g** Orthogonal XZ and YZ projections show the three-dimensional localization of a PV interneuron soma and surrounding PNNs stained with WFA. Scale bars: 10 µm. **h** Mean corrected WFA signal density is reduced in the neocortex of KO mice (*n* = 8 slices per genotype from *N* = 3 brains/genotype; Paired Student’s *t*-test: *p* = 0.046). **i** Proportion of PV interneurons enwrapped by PNNs in S1 of KO mice is unchanged (*n* = 8 slices per genotype from *N* = 3 brains/genotype; Paired Student’s *t*-test: *p* = 0.277). **h**, **i** Display individual data points with median (mid-line) and interquartile range (upper and lower lines). * *p* < 0.05.

The pattern of reduced predicted input probability onto PV interneurons suggests a potential compromise in the integrity of perineuronal nets (PNNs), a specialized extracellular matrix structure that is critical for stabilizing synaptic contacts onto PV cells and supports their high-frequency firing^14,15^. As PNNs are increasingly recognized to depend on continuous support from the oligodendrocyte lineage^65–67^, we next examined predicted communication probability between oligodendroglial populations and PV interneurons. Although proportion tests and UMAP distribution showed no changes in the overall abundance of oligodendrocyte lineage cells (Supplementary Fig. 7c–e and Supplementary Fig. 8a–c), confirmed by OLIG2 immunostaining of the oligodendrocyte lineage (Fig. 7a–c), we identified a striking reorganization of oligodendrocyte precursor cell (OPC)-derived signaling in the *Dnmt1* KO cortex. In contrast to the near-global decrease in predicted neuron-to-neuron communication, OPCs showed broadly increased outgoing interaction weights toward virtually all cortical cell types, including excitatory neurons, SST+ interneurons, and VIP+ interneurons, while predicted signaling toward PV interneurons remained essentially unchanged (Fig. 6a). This selective preservation of near-baseline OPC-PV communication probability, against a background of globally elevated OPC output, is consistent with a potential uncoupling of the PV-OPC axis rather than a general suppression of glial signaling.

Furthermore, we observed a selective reduction in the numbers of *Pdgfc*‑positive nuclei within the cluster of *Enpp6*-enriched newly formed oligodendrocytes (NFOLs), marking the so-called perineuronal oligodendrocytes^68,69^ (PNOLs) that synthesize, organize, and anchor extracellular matrix (ECM) constituents (Figs. 4b, 6c and Supplementary Fig. 8d). Although PNOLs retain transcripts encoding major myelin proteins and consequently exhibit substantial transcriptional overlap with myelinating oligodendrocytes (MOLs) (Supplementary Fig. 8e–h), their characteristic *Pdgfc*-expression and close apposition to neuronal somata have been proposed to distinguish them as a specialized, non-myelinating oligodendrocyte population^68^. Transcriptomic profiling of this *Pdgfc*-positive subcluster revealed a purely suppressive response in *Dnmt1* KO mice (Supplementary Fig. 8i), with downregulated genes being implicated in stress‑resilience, ECM‑remodeling, and structural programs (including *Sgk1*, *Fkbp5*, *Htra1*, *Zbtb16*, and *Bcl2l1*).

To better understand whether this reduction in *Pdgfc*^+^ PNOLs reflects a selective lineage bias or a broader redirection of NFOLs away from this state, we constructed a simple compartmental model of oligodendrocyte lineage progression (see Methods, Supplementary Fig. 9a). This model considers four sequential populations which are linked by defined transition and clearance rates: OPCs, *Pdgfc*-negative NFOLs (*Pdgfc*^−^ NFOL), *Pdgfc*-positive NFOLs (*Pdgfc*^+^ NFOL)—i.e., the PNOLs, and MOLs. This allowed us to relate observed cell-type ratios to underlying lineage dynamics under steady-state assumptions.

Quantifying these ratios in our snRNA-seq dataset, we found that the *Pdgfc*^−^ NFOL/OPC ratio was significantly increased in KO mice, the *Pdgfc*^+^ NFOL/Pdgfc^−^ NFOL ratio was significantly reduced, while the MOL/*Pdgfc*^−^ NFOL ratio remained unchanged between genotypes (Supplementary Fig. 9b). Within the model, this specific combination of changes is best explained by a selective reduction in the transition rate from *Pdgfc*^−^ NFOL to *Pdgfc*^+^ NFOL, while all other rates remain constant. Alternative explanations would require simultaneous changes in at least two independent parameters, making this single reduced transition rate the most parsimonious account of the data. This supports a model in which *Dnmt1* loss in PV interneurons selectively impairs the transition of NFOLs into the *Pdgfc*^+^ PNOL state from an otherwise preserved pool of NFOLs, rather than causing a global redirection of oligodendrocyte fate.

Taken together, these findings indicate that the observed transcriptional suppression in *Pdgfc* + PNOLs arises from a selective, model-supported bias against the *Pdgfc* + ECM-supporting state, rather than a global disturbance of oligodendrocyte lineage dynamics.

As PV interneuron–oligodendrocyte and reciprocal oligodendrocyte-PV communication probabilities were reweighted in *Dnmt1* KO mice (Fig. 6d), we next examined which specific ligand-receptor pairs contributed most strongly to these predicted probability changes. Outgoing signaling probability from PV interneurons to oligodendroglial cells and incoming signaling probability from oligodendroglial populations to PV interneurons are shown in Figs. 6f and 6g, respectively.

In the PV-to-glia direction, NRG1-ERBB4 signaling probability from PV interneurons to oligodendroglial cells was reduced across all oligodendrocyte subtypes examined, a pattern consistent with, but not proof of, diminished neuregulin-mediated trophic output from PV cells toward the oligodendrocyte lineage^70^. Predicted NRXN2-NLGN1 signaling onto OPCs was increased in *Dnmt1* KO samples, while CNTN1-NRCAM interactions were reduced (Fig. 6f), which may reflect a rebalancing of contact-mediated coupling between PV cells and OPCs^71^. Notably, predicted GABAergic signaling probability from PV interneurons onto OPCs via GABA-A receptors was higher in KO animals across multiple receptor subunit combinations, while GABA-B-mediated signaling probability from PV cells onto the mature oligodendrocyte subtypes was reduced (Fig. 6f).

In the reciprocal glia-to-PV direction, OPC-derived NRG1-ERBB4 signaling probability onto PV interneurons was higher, potentially reflecting a compensatory upregulation of glia-derived trophic support. In addition to this predicted trophic remodeling, SEMA-PLXN signaling probability was enhanced, particularly from differentiated oligodendrocyte populations. By contrast, predicted PTPRM-PTPRM signaling from PNOLs to PV cells, as well as NRXN-NLGN signaling from OPCs, was reduced (Fig. 6g). The concurrent probability reduction across signals involved in adhesion, contact stabilization, perisomatic ECM anchoring, and PNN cohesion^72–74^, is consistent with a reorganization of the structural interface between PV cells and their oligodendroglial partners, which may compromise perisomatic synaptic integration. Whether these predicted signaling changes are reflected at the protein or synaptic level remains to be explored in future work.

### Histological validation confirms perineuronal net deficits

Compartmental modeling and CellChat analysis, based on mRNA expression of ligand-receptor pairs, converged on a selective reorganization of the PV-associated oligodendroglial niche. We therefore asked whether these predicted changes are reflected at the tissue level in glial markers and perineuronal net integrity. Consistent with our snRNA-seq two-sample proportion tests and Milo neighborhood analyses, which revealed no significant changes in oligodendrocyte lineage abundance (Supplementary Fig. 7c–e), the overall number of OLIG2-positive oligodendroglial cells was unchanged in *Dnmt1* KO mice (Fig. 7a–c). Similarly, MBP immunostaining revealed no differences in cortical myelination between control and *Dnmt1* KO mice (Supplementary Fig. 10), consistent with the model-supported view that the myelinating trajectory is preserved. In contrast, GFAP immunostaining revealed reduced signal in the somatosensory cortex of *Dnmt1* KO mice, consistent with the astrocyte-associated alteration detected by snRNA-seq (Supplementary Fig. 7c–f). Against this background of preserved oligodendroglial numbers and myelination pattern despite altered glial niche composition, histological analysis revealed a reduction of Wisteria floribunda agglutinin (WFA)-labeled PNNs across the whole neocortex of *Dnmt1* KO mice (Fig. 7d–h and Supplementary Videos 1 and 2). This provides tissue-level support for the idea that DNMT1 loss in PV interneurons destabilizes the perisomatic glial–ECM support system surrounding PV interneurons, without evidence of gross demyelination. To determine whether this deficit followed the laminar organization of cortical circuitry, we further quantified WFA labeling across layers of the primary somatosensory cortex (Supplementary Fig. 11a–d). WFA-labeled PNN levels were significantly reduced in the combined layer 2–4 compartment and in layer 5 of *Dnmt1* KO mice, whereas no significant differences were detected in layer 1 or layer 6. The fraction of PV cells enwrapped by PNNs, however, remained unchanged in somatosensory, somatomotor, and visual areas (Fig. 7i and Supplementary Fig. 11e–h). Together, these findings suggest that *Dnmt1* loss reduces overall PNN abundance or WFA-detectable net integrity without substantially changing the proportion of PV interneurons associated with PNNs. Given that PNNs stabilize PV firing properties and gamma synchrony^15,75^, these findings implicate glial and extracellular matrix abnormalities as amplifiers of PV interneuron deficits, contributing to both the physiological (reduced PV efficacy, weakened gamma) and behavioral (apathy/anhedonia/anxiety) phenotypes in *Dnmt1* KO mice.

Collectively, these findings provide a mechanistic bridge from DNMT1 loss in PV interneurons to disrupted inhibitory signaling, defective PV-glia communication, and impaired PNN integrity, ultimately destabilizing the cellular ecosystem required for cortical network balance and gamma synchrony.

## Discussion

Here we show that deleting *Dnmt1* specifically in cortical PV interneurons leads to multi-scale changes in inhibition at the cellular, network, and behavioral levels that are associated with depression- and anxiety-like phenotypes. In vivo Neuropixels recordings revealed increased spontaneous firing of PV cells and elevated baseline activity in surrounding cortical populations, accompanied by a reduced inhibitory impact of PV activation on local circuits. The dominant consequence of *Dnmt1* deletion is therefore not reduced PV interneuron activity but diminished synaptic efficacy and a corresponding loss of inhibitory control. This is consistent with our previous finding that *Dnmt1* loss enhances GABAergic vesicle replenishment and release at PV terminals^8^. We interpret the global rise in excitatory neuron firing and the blunted response to PV photostimulation as a homeostatic network adaptation to counteract persistently elevated GABAergic drive by down-weighting inhibitory influence, a phenomenon observed in vitro and in vivo when networks strive to stabilize E/I balance^76^. Moreover, because PV interneurons form GABAergic autapses that regulate their fast-spiking dynamics and contribute to gamma synchrony^77^, the observed increase in PV firing may also represent an adaptive response to altered self-inhibition. These network adjustments likely contribute to the smaller, delayed, and less precise visually evoked responses and the loss of a feedback-inhibitory rebound across layers.

Despite these physiological changes, basic perceptual and learning capacities remained intact, suggesting a circuit-level compensation potentially via subcortical pathways that sustain task performance even when cortical precision is reduced. Nevertheless, DNMT1 loss degraded PV-dependent network dynamics across sensory (V1, S1) and prefrontal (ALM) cortical areas; PV-mediated inhibition was weakened, and visually evoked gamma oscillations were markedly reduced, in line with the central role of PV cells in generating gamma rhythms^22,23^.

*Dnmt1* KO mice exhibited an apathy- and anhedonia-like phenotype, including reduced home-cage activity, poor nest construction, deteriorated coat condition, diminished wheel running and sexual motivation, and prolonged latency in novelty-suppressed feeding. Food intake and circulating ghrelin, insulin, and PYY levels remained unaltered, whereas body weight and leptin were elevated, indicating reduced energy expenditure rather than primary hypothalamic dysregulation. Correspondingly, PV cells in the arcuate nucleus showed no overlap with POMC and only partial overlap with AgRP populations. These findings point to cortical, rather than hypothalamic, mechanisms underlying the dysphoric-like phenotype, in line with evidence linking PV interneuron dysfunction and impaired gamma synchrony to mood disorders in both humans and rodent models^13,23,36,78,79^, as well as a recent large-scale genome-wide association study implicating GABAergic signaling as a key anxiety risk factor^80^.

A central point of this study is that a PV-restricted, cell-autonomous manipulation of *Dnmt1* expression propagates across the network, leading to widespread, non-cell-autonomous adaptations. Single-nucleus RNA-seq suggested genotype-associated differences in the relative abundance of astrocytes and specific excitatory neuron populations, most notably *Tox*+/*Slc17a6+* corticospinal-like neurons, as well as transcriptional alterations of genes involved in synaptic structure and transmission.

While reduced GFAP signal in the somatosensory cortex of *Dnmt1* KO mice was in line with diminished astrocyte abundance observed in snRNA-seq, differences in excitatory neuron abundance warrant further validation. The enrichment of corticospinal-like neurons could reflect activity- or circuit-dependent transcriptional state transitions rather than the emergence of a new excitatory subtype in the adult KO cortex. Tox is a calcineurin/NFAT-responsive transcription factor whose expression is dynamically regulated by neuronal activity^81^, and corticospinal neuron identities are molecularly specified early in development^82^, arguing against de novo generation of a novel corticospinal class. In light of recent work showing that strong, sustained activity manipulations can drive broad, chromatin-linked state shifts without stable cell-type expansion^83^, we interpret the *Tox* + /*Slc17a6*+ enrichment as a hypothesis-generating, activity-dependent state change rather than definitive subtype expansion.

Beyond compositional changes, transcriptional rewiring included a cortex-wide suppression of the stress-related genes *Fkbp5* and *Zbtb16* across neurons and glia, a signature reminiscent of chronic stress and depression models^59,84^. In excitatory neurons, activity-dependent immediate early genes (*Arc*, *Fos*, *Nr4a1–3*) were also reduced, which initially seems counterintuitive given the elevated tonic firing rates. Persistent or repeated network activity has, however, been shown to blunt subsequent activity-dependent transcriptional induction, as sustained depolarization desensitizes the signaling cascades that normally couple synaptic activity to immediate early gene transcription^85,86^. Consistent with this, recent multiomic work indicates that repeated stimulation drives adaptive, chromatin-remodeled states with altered responsiveness to further input^83^. The reduced immediate early gene expression observed here may therefore reflect desensitized activity–transcription coupling in the context of chronically elevated, temporally imprecise network drive, rather than diminished neuronal engagement.

Our chromatin-level analyses in an independent cohort supported this interpretation by linking the transcriptomic changes to underlying regulatory network activity. eRegulon-based GRN inference showed that DEGs were enriched among eRegulon target genes across most cell types, indicating that transcriptional shifts are preferentially associated with chromatin-defined regulons rather than being randomly distributed. L4 sensory neurons, which also showed strong synapse-related DEGs, were the only population with significant directional concordance between chromatin accessibility and transcription, a coupling notably absent in PV interneurons themselves. While suggestive of layer 4-specific cis-regulatory remodeling downstream of PV dysfunction, these associations should be regarded as hypothesis-generating given the constraints of the underlying dataset.

Consistent with these molecular changes, circuit-level analyses and cell-cell communication modeling point to a possible laminar redistribution of inhibitory signaling. Rather than a simple strengthening of input-layer inhibition, predicted PV outputs were broadly reduced across excitatory targets in *Dnmt1* KO mice, with the strongest losses directed at deep-layer and corticofugal populations, while the decrease toward L4 sensory neurons was comparatively modest. Conversely, incoming signaling probability to PV cells from excitatory and inhibitory neurons was markedly lower, most prominently from SST+ interneurons and L2/3 and L5/6 IT neurons. This pattern is consistent with the notion that PV interneurons can become hyperactive yet functionally decoupled when excitatory afferents weaken, resulting in ineffective inhibition despite elevated firing^78,87^. Collectively, these laminar patterns suggest a rebalancing of inhibitory output from PV cells, potentially weakening deep-layer output control and distributed recurrent stability even as sensory input-layer gating is relatively spared.

Similarly, predicted communication between PV interneurons and oligodendroglial cells was markedly reweighted in the cortex of KO mice, accompanied by a selective depletion of *Pdgfc*-expressing PNOLs and reduced expression of genes maintaining ECM integrity. Oligodendrocyte lineage cells are crucial signaling partners for interneurons, responding dynamically to inhibitory signals via GABA receptors^88^. In this context, the rewiring of inferred GABAergic signaling probability in KO mice points to an imbalanced support program across the oligodendrocyte lineage. GABA-A receptor-mediated signaling probability from PV cells onto OPCs was higher, echoing prior evidence that enhanced GABA-A receptor activity promotes OPC maturation^89^. In contrast, predicted GABA-B-mediated signaling onto mature oligodendrocytes, a pathway critical for their structural maintenance and survival^90^, was reduced. Together, these opposing changes suggest a stage-specific dysregulation of PV-oligodendroglial communication that may limit effective maintenance of the perineuronal oligodendrocyte niche.

This predicted deficit is further paralleled by an asymmetric remodeling of NRG1-ERBB4 signaling probability within the PV-oligodendrocyte axis, a pattern that may reflect a compensatory but insufficient upregulation of glia-derived trophic support. Given that both NRG1 and ERBB4 are co-expressed in PV interneurons and oligodendrocyte lineage cells, such bidirectional and opposing changes within the same signaling axis are consistent with previously described NRG1-ERBB4 pathway perturbations^91^, but remain inferential and will require dedicated experimental validation at the protein and synaptic level.

Beyond trophic signaling, the coordinated decrease in key ligand-receptor interaction probability scores between PV cells and oligodendroglial cells points to impaired perisomatic structural support and reduced PNN cohesion. Neurexins (NRXNs) and neuroligins (NLGNs) form transsynaptic complexes that coordinate synapse formation and maintain structural adhesion^92,93^, and perturbations in NRXN-NLGN signaling have been shown to destabilize synaptic alignment and the structural cohesion of perisomatic synapses^94,95^. In the axo-glial context, NRXN-NLGN signaling couples presynaptic neurexins to oligodendrocytic neuroligin-3 (NLGN3) to facilitate differentiation, myelin formation, and stability^96^. The predicted reduction of OPC-derived NRXN-NLGN communication probability is therefore consistent with weakened axo-glial adhesion at perisomatic contacts. PTPRM-mediated homophilic interactions stabilize cell-cell contacts involved in synapse formation^97^, while CNTN1–NRCAM interactions anchor cell-surface glycoproteins to the ECM scaffold at perisomatic contacts^98^. Together with the observed decrease in OPC-derived NRXN–NLGN signaling probability, the diminished CNTN1–NRCAM and PTPRM–PTPRM interaction scores from PNOLs support a coordinated weakening of perisomatic adhesion, though this remains to be tested directly. The concurrent increase in SEMA–PLXN signaling probability from differentiated oligodendrocytes likely reflects a reactive structural remodeling response, consistent with their reported functions in PNN architecture^99^. Collectively, these model-derived findings nominate altered neuron–glia communication as a candidate mechanism linking PV dysfunction to reduced PNN stability and impaired network coordination^14^.

In addition to this predicted reorganization of ligand-receptor signaling, the reduced abundance of *Pdgfc*^+^ PNOLs itself raises the question of whether *Dnmt1* loss in PV interneurons broadly destabilizes the oligodendrocyte lineage or instead narrows entry into this specific state. Our compartmental modeling of lineage transitions supports the latter: the fate bias is selective rather than global, sparing the myelinating trajectory and overall myelination capacity. This suggests that the ligand-receptor changes described above are not simply a consequence of fewer oligodendroglial cells overall, but instead reflect a genuine shift in signaling from a numerically reduced, yet still-communicating, PNOL population. This reinforces a targeted disruption of the PV-supporting niche rather than a general glial vulnerability, consistent with the selective loss of WFA-labeled PNNs described below.

Building on this niche-specific mechanism, elevated PV activity and altered spiking patterns may drive activity-dependent glial plasticity that reshapes PNN and neuro-glial positioning within this specific oligodendroglial subpopulation. Histological validation confirmed this systems-level implication, showing diminished net intensity of WFA-labeled PNNs in KO mice despite preserved total OLIG2+ cell numbers and myelination levels in the neocortex. This is consistent with the established role of PNNs in maintaining PV interneuron excitability, synaptic integration, and gamma synchrony^14^. Indeed, PNN degradation has been shown to weaken gamma oscillations and cognitive precision^14,100^, providing a mechanistic link between synaptic inhibition and network disruptions.

As PNNs stabilize PV excitability and support oscillatory coherence, their loss provides a functional bridge between reduced PV efficacy, weakened gamma power, and mood-related behavior. We propose a feed-forward model in which DNMT1 loss increases presynaptic GABA release, triggering homeostatic desensitization of inhibitory signaling and glial remodeling that culminates in ECM degradation and network desynchronization. The resulting disruption of PNNs may further destabilize PV function, amplifying network desynchronization and dysphoric behavior.

This cascade reconciles diverse GABAergic findings in mood disorders: alterations in PV efficacy, gamma coherence, and PNN integrity can occur without cell loss, reflecting broader neuron-glia-ECM dysregulation. By positioning DNMT1 as an upstream regulator of this axis, our results highlight an epigenetic mechanism by which inhibitory microcircuits coordinate with glial cells and extracellular scaffolds to sustain cortical timing.

## Methods

### Animals

Transgenic *Pvalb-Cre/tdTomato/Dnmt1 loxP*^2^ mice were used as knockout animals and *Pvalb-Cre/tdTomato*-mice as controls. Knockout and control strains were bred separately. Unless stated differently, all experiments were conducted using male mice aged 3–6 months. Both mouse lines were established on a C57BL/6-background by crossing in the *Pvalb-Cre* line (obtained from Christian Huebner, University Hospital Jena, Germany, and described in Hippenmeyer et al.^101^) and the *tdTomato* transgenic reporter mouse line (obtained from Christian Hübner, University Hospital Jena, Germany, and described in Madisen et al.^102^), leading to postnatal Cre expression largely restricted to fast-spiking interneurons. To obtain *Dnmt1*-knockout mice, *Dnmt1* floxP^2^ mice (B6;129Sv-Dnmt1^tm4Jae^/J, Jaenisch laboratory, Whitehead Institute; USA; Jackson-Grusby et al.^103^) were crossbred, in which LoxP-sites are flanking exons 4 and 5 of the *Dnmt1* gene. This results in a null *Dnmt1* allele upon Cre-mediated excision^103^. For both the control and knockout strains, Cre-positive females were mated with Cre-negative males to avoid unintended germline recombination^104^. Correct genotypes were validated with the following primers: *Dnmt1* forward 5′-GGGCCAGTTGTGTGACTTGG and reverse 3′-CCTGGGCCTGGATCTTGGGGA (results in a 334 bp wildtype and 368 bp mutant band); *tdTomato* wildtype forward 5′-AAGGGAGCTGCAGTGGAGTA, wildtype reverse 3′-CCGAAAATCTGTGGGAAGTC, mutant forward 5′-CTGTTCCTGTACGGCATGG and mutant reverse 3′-CTGTTCCTGTACGGCATGG (results in a 297 bp wildtype and 196 bp mutant band); *Pvalb*-*Cre* forward 5′-AAACGTTGATGCCGGTGAACGTGC and reverse 3′-TAACATTCTCCCACCGTCAGTACG (resulting in a 214 bp fragment). All animal procedures were performed in strict compliance with the EU directives 86/609/EWG and 2007/526/EG guidelines for animal experiments and were approved by the local governments (Thueringer Landesamt, Bad Langensalza, Germany and Landesamt für Natur: animal license: 02-008/12**)**, Umwelt und Verbraucherschutz Nordrhein-Westfalen, Recklinghausen, Germany: animal licenses 81-02.04.2020.A175, 81-02.04.2021.A021). Unless indicated otherwise, animals were housed under standard housing conditions: mice were kept in compatible groups, under 12 h light/dark conditions, with ad libitum access to food and water, and nestlets and tunnels as environmental enrichments. The weight of individual animals was determined prior to behavioral experiments or prior to sacrificing and brain preparation.

### Electrophysiological recordings

#### Surgeries

For all surgeries, isoflurane in oxygen was used as anesthesia (3% isoflurane for induction, 1–2% for maintenance), and subcutaneous injections of carprofen (4 mg/kg, Rimadyl, Zoetis GmbH) and buprenorphine (0.1 mg/kg, Buprenovet sine, Bayer Vital GMBH) for analgesia. Eye ointment (Bepanthen, Bayer Vital GmbH) was applied to their eyes to prevent damage from drying out. After medially incising the dorsal skin of the head, it was pushed outward and fixed in place utilizing tissue adhesive (Vetbond, 3 M). Dental cement (C&B Metabond, Parkell; Ortho-Jet, Lang Dental) was applied on top of the tissue adhesive to create a support structure for a custom-built circular headbar.

Viral injections (AAV1.shortCAG.dlox.hChR2(H134R).WPRE.hGHp, Zurich Vector Core, viral titer = ~8×1012 vg/ml) were carried out according to the experimental scheme. In naive mice (Fig. 1 and Supplementary Fig. 1), S1 and V1 were targeted unilaterally, whereas in trained animals (Supplementary Fig. 2e–j) bilateral injections in ALM were conducted. The positions of all injections were chosen by stereotactic coordinates (ALM: AP 2.5/ ML -1.5; V1: AP -4/ ML -2; S1: AP -2/ ML -3.5). Injections were conducted using a thin glass pipette with a micropump (Nanoject3, Drummond Scientific) after thinning out the skull with a dental drill. At each site, two quantities of 200 nl were injected at different cortical depths (300/600 µm), adding up to a total volume of 400 nl virus per target region. The 200 nl were injected in 20 steps using 10 nl portions at 14 s intervals. For electrophysiological recordings, circular craniotomies were performed using a biopsy puncher and then covered with a round coverslip of similar size using light-curable dental cement (DE Flowable composite, Nordenta) to fix it in place. The coverslips contained a small opening to later allow access for the Neuropixels probe. Between recordings, this opening was covered with silicon. A thin layer of cyanoacrylate (Zap-A-Gap CA+, Pacer technology) was used to cover the skull. After the surgery, buprenorphine (0.1 mg/kg body weight) was again injected subcutaneously. The animal’s home cage was kept on a heating pad following the surgery until the mouse recovered from anesthesia. To prevent inflammation of the surgical wounds, the animals received a subcutaneous injection of carprofen on post‑op days 1 and 2 while the animal’s drinking water was supplemented with antibiotics (enrofloxacin 0.025 mg/ml) and analgesics (buprenorphine 0.0125 mg/ml) for at least three days. Progression of wound healing and the animal’s general health were scored daily following the surgery.

To record from naive animals, two mice per genotype were utilized; all of them were approx. 6 months of age during surgery. For behavioral analysis and recordings in trained mice, 7 animals (3 control/4 knockout) were used; they were approx. 8 weeks old during surgery.

#### Neuropixels recordings

Electrophysiological recordings were carried out in head-fixed mice using Neuropixels 1.0 probes. Recordings were carried out on four consecutive days. Probes were painted with DiD cell labeling solution (Invitrogen V22887) before each recording to later identify recording sites in fixed brain slices.

The stimulation protocol was started 5–10 min after insertion, and we recorded high-pass-filtered data above 300 Hz at 30 kHz and low-pass-filtered signals between 0.5–100 Hz at 2.5 kHz from the bottom 384 channels of the Neuropixels probe ( ~ 3.8 mm active recording area). Signals were acquired with an external Neuropixels PXIe card (IMEC, Belgium) used in a PXIe chassis (PXIe-1071, National Instruments, USA). Triggers and control signals for different stimuli were separately recorded as analogue and digital signals using the SpikeGLX software (Janelia Farm Research Campus, USA; Bill Karsh, https://github.com/billkarsh/SpikeGLX).

The visual stimulus to test sensory responses in V1 and ALM consisted of a 0.1-s-long full-field low-pass filtered Gaussian noise pattern with a cutoff at 0.12 cycles per degree and a temporal frequency of 1 Hz^105^. In each trial, the stimulus was presented twice, with an inter-stimulus interval of 0.5 s. Visual stimulation to induce gamma oscillations consisted of 5-s-long full-field drifting square-wave gratings at horizontal orientation and a spatial frequency of 0.04 cycles per degree. All visual stimuli were presented at 17-cm distance from the right eye on a gamma-corrected LED backlit LCD monitor (Viewsonic VX3276-2K-MHD-2, 32”, 60 Hz refresh rate). The overall stimulation protocol consisted of 50 trials of each type, presented in randomized order with inter-trial intervals between 3–5 s.

For optogenetic stimulation, we used a fiber-coupled laser system (ReadyBeam Bio1, FISBA AG), connected to a 105 µm glass fiber (0.22 NA) that was positioned over the cortical recording site. Optogenetic stimulation consisted of a 3-s long pulse at 488 nm with a peak power of 10 mW from the fiber tip. To avoid transient changes in PV activity, the pulse intensity was first increased for 1 s, then remained at a peak intensity for 1 second, and lastly decreased back to zero for 1 second. The sequence of visual, optogenetic, and combined trials was fully randomized. For optotagging of PV interneurons, we presented 20 optogenetic stimulus sequences at 5 Hz for 1 s, with an individual pulse length of 10 ms and a peak power of 2 mW from the fiber tip. PV interneurons were then identified by short-latency responses (<2 ms) to individual laser pulses and an increase in firing rate during the 3-s-long optogenetic stimulation period.

To extract spiking activity, channels that were broken or outside of the brain were detected and removed from the recording using the Spike Interface analysis toolbox^106^. The remaining channels were then time-shifted and median-subtracted across channels and time. Corrected channels were then spike-sorted with the Kilosort 2.5 software and manually curated using phy (https://github.com/cortex-lab/phy). All sorted spiking and local field potential data were then analyzed using custom MATLAB code (2020b, MathWorks).

Peri-event time histograms (PETHs) for each cluster were computed with a bin size of 10 ms and baseline-subtracted by the mean activity within 1 second before the laser onset. Trial-averaged local field potentials (LFPs) in response to visual stimulation were similarly baseline-corrected. To compute current source densities (CSDs) from LFP signals, we used the inverse CSD method by Pettersen et al.^107^. We applied the spline iCSD method, assuming a smoothly varying CSD between electrode contacts, based on polynomial interpolation. We assumed a homogeneous, isotropic conductivity of *σ* = 0.3 S/m within and directly above cortex^107^. To reduce spatial noise, the estimated CSD was subsequently convolved with a Gaussian spatial filter with a standard deviation of 0.1 mm. For LFP and CSD analyses, the signals from neighboring contacts at the same depth were averaged together to improve signal quality.

#### Behavioral assays

##### Evidence accumulation task

After full recovery from surgeries, animals were water-deprived for one day before introducing them to the behavioral task. To obtain rewards, mice needed to accumulate visual evidence in the form of visual grating-like cues and indicate the side with the higher number of cues. Experiments were conducted in a custom-built setup with stimulus presentation, lick detection, and water reward controlled by a microcontroller (Teensy 3.2, PJRC) and Python code (Python 3.7). In general, the task consisted of three periods: 3 s sensory stimulation with randomized sequences of up to 6 cues on one or both sides, 0.5 s delay, and a 2-s response window. In between trials was a 3.5-s inter-trial interval, which was prolonged after missed trials or incorrect responses. Mice first learned to reliably detect visual stimuli by indicating the side where all 6 cues (spatial frequency of 0.018 cpd, temporal frequency of 2 Hz) were shown. After mice performed more than 75% of trials correctly in 3 out of the last 4 sessions, distractor stimuli were added on the non-rewarded side. The number of cues on both the target and distractor side varied, resulting in different levels of difficulty depending on the absolute difference in cues shown on either side. Training was conducted using 5 control and 7 knockout animals.

##### Morris water maze (MWM)

Visual navigation and learning capacities were investigated using an MWM. The setup consisted of a cylindrical water-filled pool (Ø 1 m) surrounded by an opaque curtain, four distinct landmarks on the pool wall, and a transparent platform (Ø 10 cm). The setup was divided into four virtual quadrants with the platform being consistently in the same quadrant. The start position of the mouse was pseudo-randomly alternated between trials. On the first day of testing, mice started once per quadrant for habituation. The platform slightly protruded from the water level to facilitate quicker locating. Upon finding the platform, the animal was required to remain on top of it for 5 s for the trial to be counted successful. If an animal did not find the platform or left it after less than 5 s and did not return onto it within the 90-s period of the trial, the mouse was manually guided to the platform before taking it back to its cage. Between trials, mice were kept under red light for at least 10 min. For the actual learning task, the platform was submerged (1–2 cm), and the maximum duration per trial was set to 60 s. Each mouse started three times per quadrant in a pseudo-random order, equaling 12 trials per day. This training scheme was conducted on five consecutive days. Mice were recorded from above, and behavioral data were processed using ANY-maze (Stoelting Co., Wood Dale, USA). We tested 8 control and 8 knockout mice.

##### Home cage activity

To assess the animals’ activity in their home cages, cameras were set up in front of cages of group-housed animals. Each cage was filmed on several separate days with a frame rate of 25 frames per second. From each of these recordings, eight uninterrupted 30 min periods were used for further analysis. For each time period, a pixel-wise comparison with ImageJ was utilized to calculate the difference between every fifth frame of a video. For this, we used the ImageJ Image Calculator and adjusted the threshold to exclude background noise. Subsequently, we measured the area of differing pixels. We filmed 3 cages per genotype, housing 2 mice each (6 control/6 knockout).

##### Rotarod

A rotarod was used to assess balance and motor performance of mice. Initially, the mice (13 control/11 knockout) were individually habituated for 4 min at a constant speed of 4 rpm. The subsequent test phase comprised three consecutive trials, each lasting a maximum of 5 min, with continuous acceleration from 4 to 40 rpm.

##### Wire hang test

To record neuromuscular strength, mice were placed on a metal wire cage lid, which was then slowly inverted and positioned over a type IV Makrolon cage. Each mouse was given three trials of max. 5 min, with inter trial intervals of approx. 3–4 min. In total, 24 mice (13 control/11 knockout) conducted this experiment.

##### Food intake

Food intake of group-housed mice was quantified by assessing the change in cumulative pellet weight after either 24 h or 72 h. Animals remained in their familiar cage settings, and pellet weight measurements were calculated relative to the number of animals per cage as the consumed amount per day. Tests were conducted on 4 control and 4 knockout cages (each cage containing 2 mice: 8 control/8 knockout). For each cage, two 24-h periods and eight 72-h periods were recorded.

To measure food and water intake in single-housed mice, mice were kept individually for at least two weeks before measuring pellet and water bottle weight of each cage on five consecutive days. For this, we used 4 control and 6 knockout mice.

##### Nest-building test

Nest-building tests were executed as described by Deacon^108^ and as visualized in Fig. 2f. In brief, mice were weighed and transferred to individual testing cages (IVC cages, Tecniplast) with ad libitum access to food and water, but without any additional environmental enrichment. One nestlet of known weight was placed in each cage. The test was initiated at 5 p.m., and nest-building skills were scored the next morning at 9 am. Nests were scored according to the following criteria: Score 1 = no noticeable interaction with the nestlet (more than 90% of the nestlet remained intact), Score 2 = nestlet was partially torn (50–90% of the nestlet remained intact), Score 3 = nestlet was largely torn, but no identifiable nest site was build (10–50% of the nestlet remained intact, but less than 90% of shredded material is gathered within a quarter of the cage area), Score 4 = nestlet was used to build a flat nest (less than 10% of the nestlet remained intact, less than 50% of the nest circumference is build higher than the resting mouse), Score 5 = nestlet was used to build a (near) perfect nest (less than 10% of the nestlet remained intact, more than 50% of the nest circumference is build higher than the resting mouse). Each mouse was tested twice, with tests being one week apart from each other. In the time between tests, mice remained single-housed without access to further enrichments besides the handed nestlet. Overall, 49 mice (24 control/25 knockout) at ages between 3–6 months were tested.

##### Coat state

The animals’ coat state was assessed in a blinded manner. The scoring system was adapted from Nollet et al.^47^ as visualized in Fig. 2g. Seven different regions of the animal’s body were scored individually with one of three distinct ranks. In case of a good coat state (smooth and shiny fur, no tousled or spiky patches) rank 0 was assigned. Strongly matted, oily, or even stained fur was assigned a rank of 1. Slight deviations from the optimum coat state were ranked with a 0.5. Among the investigated areas were both dorsal (head, neck, back, tail) and ventral areas (fore paws, abdomen, hind paws). Overall, 12 control and 12 knockout mice (2–7 months) were investigated.

##### Voluntary wheel running

Single-housed mice were kept in cages with access to running wheels (TSE Systems). Animals were housed for up to three days in these cages. While food and water were provided ad libitum, no further enrichment, despite the running wheels, was provided. Each animals’ wheel usage was continuously recorded and subsequently evaluated using a custom-written Python script. In total, 11 control and 9 knockout mice were utilized.

##### Interaction test

To test for social interest in female conspecifics, virgin C57BL/6J females were transferred to cages of virgin transgenic, single-housed males. These encounters were filmed for 10 min to evaluate the behavior of the transgenic males. Behaviors such as sniffing, grooming or mounting of the female intruder were evaluated second-wise. The same female encountered two males per day, one per genotype. Between these encounters were at least 6 h to ensure that the females did not transfer any noticeable odors from the first encounter during the second session of the day. This way, we were able to compare the reaction of males of different genotypes to the same female during the same time of her estrus cycle. A total of 16 animals (8 controls/8 knockouts) were utilized.

##### Female urine sniffing test

The reaction of transgenic males towards female olfactory cues was tested using female urine. For this, urine from 5–10 C57BL/6J-females from different cages was collected and mixed. To circumvent reactions of the tested males towards the presentation medium, pieces of parafilm were kept in the animals’ cages for one hour prior to testing them. Then, 40 µl tap water was applied to the parafilm as a neutral cue, and the time spent sniffing at the application site was assessed for 3 min. After a 45 min break, the same test was conducted using urine instead of water. A total of 16 animals (8 controls/8 knockouts) were utilized.

##### Novelty-suppressed feeding test

Experiments were conducted according to Samuels and Hen^109^ and as visualized in Fig. 3d. In brief, mice were weighed and transferred to individual cages (IVC cages, Tecniplast) with environmental enrichments and *ad libitum* access to water. After 24 h of food deprivation, the novelty-suppressed feeding test was conducted. For this, mice were individually placed in an arena (i.e., a mouse shipping container with bedding on the ground) with a food pellet attached to a platform in the center. Mice were filmed from above to later analyze their movement patterns using DeepLabCut^110^. Once a mouse started consuming the presented pellet, the animal was transferred to its home cage, and a food pellet of known weight was placed in the food tray. After the test, all animals were weighed again. In total, 20 mice (10 control/ 10 knockout) aged 3–10 months were tested.

##### Luminex blood analysis

To test for hormonal blood concentrations, blood samples were collected *post-mortem*. For each mouse, two samples of 50 µl blood serum were processed. Samples were purified and analyzed using the Luminex System (Millipore). Using antibody-coated beads, a multiplex assay was performed to assess changes in hormone levels being quantified using the MAGPIX detection system. The MILLIPLEX Analyst 5.1 software then calculated hormone levels of ghrelin, GLP-1, glucagon, insulin, leptin, PYY, and TNFα in pg/ml. A total of 6 control and 6 knockout mice, all aged 6 months, were used.

##### Brain tissue preparation

Mice were anesthetized using 5% (v/v) isoflurane before decapitation. For immunohistochemistry, mice were transcardially perfused first with phosphate-buffered saline (PBS; pH 7.4), then with 4% paraformaldehyde (PFA) in PBS (pH 7.4). Subsequently, brains were prepared and kept in 4% PFA for 24 h at 4 °C for post‑fixation. Cryoprotection was achieved by incubating the samples in 10% (v/v) and then in 30% (v/v) sucrose in PBS for 24 h each. Brains were subsequently frozen on dry ice and stored at −80 °C.

##### Single-nucleus RNA sequencing

Neocortical tissue of *Pvalb-Cre/tdTomato* and *Pvalb-Cre/tdTomato/Dnmt1 loxP*^2^ mice was dissected from 1-mm-thick coronal slices corresponding to the region between Bregma +1 and −2. For each genotype, tissue from the three mice was pooled prior to nuclei extraction to obtain sufficient material and minimize inter-individual variability. The samples were snap-frozen on dry ice and stored at −80 °C until nuclei extraction to preserve RNA integrity. The nuclei were extracted, fixed and stored until library preparation as previously described^9^. The snRNA-seq libraries were generated using the Evercode™ WT kit (Parse Biosciences, USA). The quality of the cDNA and the final libraries was verified using the High Sensitivity D5000 and D1000 ScreenTape kits (Agilent Technologies, USA). Libraries were equimolarly pooled and sequenced together with 5% PhiX spike-in library on an Illumina NovaSeq machine running in paired-end mode with the S2 Reagent Kit v1.5 (200 cycles; Illumina, USA).

The sequencing dataset, with a mean of 97,031 and 92,265 reads/cell and a mean of 5502 and 5385 genes/cell for the control and KO conditions, was processed and filtered using the default parameters in Trailmaker™ (Parse Biosciences, 2025). Nuclei were filtered based on a minimum cell size (range: 2610–3,470 UMIs), a maximum mitochondrial fraction threshold (absolute threshold method; range: 0.51–0.62%), and a gene-vs-UMI spline regression filter (*p* < 0.0002). Doublets were removed using a sample-specific probability threshold (range: 0.37–0.45). Cell classification was performed at FDR = 0.01. Data integration was performed using Seurat’s log-normalization on the top 2,000 variable genes, followed by dimensionality reduction via RPCA (27 principal components). UMAP embedding was computed using cosine distance (minimum distance = 0.3), and clusters were identified using the Leiden algorithm at resolution 0.8. After processing, a total of 9008 and 12,141 high-quality nuclei were retained for the control and KO samples, respectively (median nCount: 26,086 and 25,257; median nFeature: 5036 and 4884). The generated dataset was downloaded as a Seurat object and analyzed further using the Seurat^111^ package (5.4.0) in R (4.4.3). For visualization, a uniform manifold approximation and projection (UMAP) embedding was computed to represent cell clusters in a low-dimensional space. Cell clusters containing fewer than 0.3% of the total nuclei were removed to minimize potential artifacts arising from low-quality clusters. Cluster-specific marker genes were identified using the presto package implementation of the Wilcoxon rank-sum test, comparing each cluster against all other cells. The top 50 upregulated and top 50 downregulated marker genes per cluster (Supplementary Data File 3) were cross-referenced with established cell type-specific markers from published literature and publicly available reference databases^53,112–115^.

The annotated clusters were subsequently used to infer cell–cell communication probabilities using the CellChat^116^ database and the corresponding R package (CellChat version 2.2.0.9001).

##### Mathematical modeling

To explore mechanistic explanations for the reduction of *Pdgfc*-positive perineuronal oligodendrocytes in KO mice, we constructed a simple compartmental model of oligodendrocyte lineage progression. We considered four populations: oligodendrocyte precursor cells (OPC), *Pdgfc*-negative newly formed oligodendrocytes (*Pdgfc*^−^ NFOL), *Pdgfc*-positive newly formed oligodendrocytes (*Pdgfc*^+^ NFOL), and myelinating oligodendrocytes (MOL). OPCs give rise to *Pdgfc*^−^ NFOL, which can either differentiate into *Pdgfc*^+^ NFOL or MOL.

The dynamics were described by a system of ordinary differential equations with first order transition and clearance kinetics:

$$\frac{d}{{dt}}{c}_{{OPC}}=\alpha -\beta \cdot {c}_{{OPC}},\,\frac{d}{{dt}}{c}_{{{NFOL}}^{-}}=\beta {\cdot c}_{{OPC}}-\left(\gamma +\delta \right)\cdot {c}_{{{NFOL}}^{-}}\,$$

$$\frac{d}{{dt}}{c}_{{{NFOL}}^{+}}=\gamma {\cdot c}_{{{NFOL}}^{-}}-\upsilon {\cdot c}_{{{NFOL}}^{+}},\,\frac{d}{{dt}}{c}_{{MOL}}=\delta \cdot {c}_{{{NFOL}}^{-}}-\mu \cdot {c}_{{MOL}}$$

Here, α denotes the influx into the OPC compartment, β the transition rate from OPC to *Pdgfc*^−^ NFOL, γ and δ the transition rates from *Pdgfc*^−^ NFOL to *Pdgfc*^+^ NFOL and MOL, respectively, and ν and μ the clearance rates of *Pdgfc*^+^ NFOL and MOL due to cell turnover.

Assuming a steady state of flux, where newly formed cells replace cleared cells, the model yields simple expressions linking observable cell-type ratios to underlying transition rates. The ratios *Pdgfc*^−^ NFOL/OPC, *Pdgfc*^+^ NFOL/*Pdgfc*^−^ NFOL and MOL/Pdgfc^−^ NFOL, in particular, depend on β, γ, δ, ν and μ in defined ways. We compared these ratios between pooled Ctrl and KO samples using two-sided Wald tests on natural log-transformed ratios with Holm correction for multiple testing.

##### Single-nucleus ATAC sequencing

Neocortical tissue of *Pvalb-Cre/tdTomato* and *Pvalb-Cre/tdTomato/Dnmt1 loxP*^2^ mice was dissected, pooled, and the nuclei extracted as described above for snRNA-seq. The snRNA-seq libraries were generated from freshly isolated nuclei using the Chromium Next GEM Single Cell ATAC Reagent Kit v2 (10x Genomics, USA). The quality of the final libraries was verified using the D1000 ScreenTape kit (Agilent Technologies, USA). Libraries were equimolarly pooled and sequenced together with 1% PhiX spike-in library on an Illumina NovaSeq machine running in paired-end mode with the S1 Reagent Kit (100 cycles; Illumina, USA).

Fragment files and peak-barcode matrices were generated using Cell Ranger ATAC (10x Genomics), yielding a post-normalization median of 93,361 and 29,105 fragments/cell for the Ctrl and *Dnmt1* KO libraries, respectively (2,871 and 11,481 cells). To account for the resulting imbalance in cell numbers between genotypes, sequencing depth was normalized across libraries prior to comparison, and downstream differential accessibility, motif enrichment, and eRegulon analyses required a minimum cell threshold per group and used non-parametric statistical tests robust to unequal sample sizes.

Fragment files and peak-barcode matrices were loaded into Signac^117^ (1.17.1); chromatin assays were built with a minimum of 10 cells and 200 features per cell, and standard QC metrics were used to filter low-quality cells (nCount_peaks between 1,000–50,000, TSS enrichment > 1, nucleosome signal < 4). Normalization used TF-IDF followed by LSI (RunSVD), and clustering/UMAP embedding were computed on LSI dimensions 2–30 using the SLM algorithm at resolution 0.5, excluding the first LSI component due to depth correlation. Gene activity scores were used as an RNA proxy for label transfer, with anchors found via FindTransferAnchors (dims 1:30, k.anchor = 10, k.filter = 100, k.score = 30) and labels assigned per cluster by confidence-weighted majority vote. Peak-gene links were computed with LinkPeaks (500 kb window, score cutoff 0.05). RNA counts/metadata and ATAC metadata/fragments were exported from Seurat for input into SCENIC + ^118^ (1.0a2). In Python (3.11.4), pseudobulk ATAC tracks per cell type were used for MACS3^119^ (3.0.4) peak calling (shift 73, extsize 146, *q* = 0.05), merged into blacklist-filtered consensus peaks. A cisTopic object was built, and LDA models (15–40 topics) were fit, selecting 30 topics by likelihood, Arun, Cao-Juan, and Mimno metrics^120–123^. Motif enrichment (pycistarget/cisTarget, mm10 SCREEN v10) was run on binarized topics and differentially accessible regions. A SCENIC+ object (metacells of ~5 cells, TF-region search 1kb–150 kb) was built to infer eRegulons, filtered, and scored (AUC). Differential eRegulon activity and target enrichment (KO vs Ctrl, split by cell type) were assessed using the Wilcoxon rank-sum test, and Benjamini-Hochberg FDR correction was applied to prioritize sensitivity to capture the breadth of transcriptional targets relevant to network topology.

##### Immunohistochemistry

For immunohistochemistry of free-floating, 40-μm-thick, adult brain sections, slices were first washed three times in PBS/0.05% Triton X-100. GABA-staining required incubation in 1 N HCl for 10 min at 4 °C, then in 2 N HCl for 10 min at room temperature, and lastly in 2 N HCl for 20 min at 37 °C. Afterwards, all sections were placed in citrate buffer (0.05% Tween20, pH 6) for 15 min at 90–100 °C. After three washing steps with PBS/0.05% Triton X-100, sections were blocked for 2 h using blocking solution (4% BSA and 10% NGS in PBS/0.05% Triton X-100). Incubation with primary antibodies was conducted for 60 h at 4 °C (rabbit anti-GABA (#A2052, 1:750), rabbit anti-β-Endorphin (#Ab5028, 1:1000), mouse anti-RFP (#MA5-15257, 1:500)). Secondary antibodies were applied for 3 h at room temperature (Cy5-donkey anti-rabbit (#A10523, 1:1000), Cy3-goat anti-mouse (#115035062, 1:1000)). Sections were then stained with DAPI (100 ng/mL in PBS; Sigma-Aldrich) for 5 min. Afterwards, they were mounted onto object slides in pre-warmed 0.5% gelatine supplemented with 0.05% KCrS_2_O_8_ x H_2_O before being embedded in Mowiol (Carl Roth).

For the staining of perineuronal nets, 30-μm-thick adult brain cryosections were collected on glass slides. Sections were washed three times in PBS/0.2% Triton X-100. After blocking for 2 h using blocking solution (4% BSA and 10% NGS in PBS/0.2% Triton X-100), the slices were incubated with Wisteria floribunda lectin (WFA; #L32481, Thermo Fisher Scientific, 1:200) at 4 °C overnight. After three rounds of washing with PBS/0.2% Triton X-100, the slices were either stained with DAPI (100 ng/mL in PBS, Carl Roth) for 15 min, washed twice with PBS, and embedded in Mowiol (Sigma-Aldrich) or embedded directly in Fluoromount-G with DAPI (#E143399, Thermo Fisher Scientific).

To stain myelin sheaths and astrocytes, 30-μm-thick adult brain cryosections were collected on glass slides, washed three times, and blocked as described above. Sections were subsequently incubated overnight at room temperature with either rabbit anti-MBP antibody (#78896S, Cell Signaling Technology, 1:50) or rabbit anti-GFAP antibody (#12389, Cell Signaling Technology, 1:400). After three washes with PBS containing 0.2% Triton X-100, sections were incubated with Cy5-donkey anti-rabbit antibody (#A10523, Thermo Fisher Scientific, 1:1,000) for 2 h at room temperature. Following three 20-min washes with PBS/0.2% Triton X-100, sections were counterstained with DAPI (100 ng/mL in PBS; Carl Roth) for 20 min, washed twice with PBS for 5 min, and mounted in Fluoromount-G (#E143399, Thermo Fisher Scientific).

##### Microscopy and image analysis

The brain slices were imaged using a Leica DMi8 fluorescent microscope in combination with a THUNDER® imager unit (Leica, Germany) and the corresponding software LASX (Leica, Germany). Post‑processing was performed in the LASX software using the “Mosaic Merge” and “Thunder Lightning” tools. A maximum intensity projection of the merged tile scans was processed using Fiji^124^ (ImageJ, NIH, USA). Additionally, PV interneurons and surrounding PNNs were captured using a Leica SP8 Tau-STED3X confocal microscope (Leica, Germany), and the software LASX (Leica, Germany). The resulting z-stacks were processed with the Fiji software (ImageJ, NIH, USA), applying a 50-pixel sliding paraboloid background subtraction.

Images of MBP and GFAP stainings were acquired using a Keyence BZ-810 fluorescence microscope (Keyence, Japan). Image analysis was performed using Fiji/ImageJ (NIH, USA) for MBP and QuPath^125^ version 0.6.0 for GFAP. For the quantification of GFAP signal, defined regions of interest (ROIs) were established within the somatosensory cortex based on standard neuroanatomical landmarks. This analysis was strictly confined to the gray matter, explicitly excluding adjacent white matter structures such as the corpus callosum. For the evaluation of MBP signal across the cortex, an intensity profile was generated in Fiji by drawing three line selections per anatomical area. The resulting fluorescence intensities were then normalized to the relative cortical depth, spanning from layer 1 (0%) to layer 6 (100%), ensuring the underlying corpus callosum was excluded from the analysis.

To analyze fluorescence intensity measurements of WFA stainings, regions of interest (ROI) were manually defined, and the mean corrected density of each ROI was calculated by normalizing the corrected integrated density to the ROI area. For the analysis of PV interneurons enwrapped in PNNs, QuPath was used. Based on a groovy script for QuPath developed for this study, PV interneurons in the primary somatosensory cortex (S1), somatosensory areas (SS), somatomotor areas (MO), or visual areas (VIS) were identified, and the surrounding region was analyzed for the presence of WFA signal.

##### Gene set enrichment analysis of bulk RNA-seq data

Gene set enrichment analysis was performed using ShinyGO v0.80^126^ with the Co.Expression.Gene.Perturbations.from.GEO database. Briefly, upregulated DEGs from *Pvalb-Cre/tdTom/Dnmt1 loxP*^2^ mice were tested against the “.up” signatures and downregulated DEGs against the “.down” signatures. Enrichment significance was assessed using the hypergeometric test with FDR correction for multiple testing.

##### Statistics

Statistical tests were carried out using MATLAB (2022b), Python (2.7), and R (4.4.3). The data were tested for normal distribution using the Kolmogorov-Smirnov test. Normally distributed data were tested using a two-tailed Student’s *t*-test, one-way ANOVA, or two-way ANOVA. Non-normally distributed or ordinal data were tested using a Wilcoxon rank-sum test. Tukey’s test was conducted as a post-hoc test. The tests conducted for individual experiments are indicated in the text.

Statistical comparisons for repeated recordings or behavioral tests in the same animals with nested statistics were done with linear mixed-effects (LME) models, using the fitlme function. The model included genotype as a fixed effect and animal identity and recording number as random intercepts to account for the nested data structure arising from multiple recordings and neurons per animal. Statistical significance was then assessed via likelihood ratio tests comparing the full model to a reduced null model containing only the random intercepts. This approach ensures that genotype effects are estimated against the appropriate between-animal variance.

## Supplementary information

Transparent Peer Review file

Supplementary Information and Figures

Description of Additional Supplementary Files

Supplementary Data File 1

Supplementary Data File 2

Supplementary Data File 3

Supplementary Video 1

Supplementary Video 2

## Data Availability

All data supporting this study’s findings are publicly available through community-endorsed repositories. The snRNA-seq and snATAC-seq datasets are deposited in the Gene Expression Omnibus (GEO) repository under the accession numbers GSE312941 and GSE341363, respectively. Any additional supporting data, including behavioral measurements, electrophysiological recordings, and histological analyses, are available under: https://doi.org/10.6084/m9.figshare.33752629 or as supplementary data files.

## Code availability

Single-nucleus RNA-seq and single-nucleus ATAC-seq data were analyzed using publicly available R and Python packages, including Seurat and Signac for the pre-processing, integration and clustering of the data, CellChat for cell-cell communication inference, and SCENIC+ for the pseudo-multiome integration of both datasets. The codes for the single-nucleus analyses as well as the groovy script for the WFA analyses are deposited under: https://github.com/cxnby/PV_glia/. For electrophysiological analyses, scripts used previously were applied^9^ which can be found under: https://github.com/musall/dnmt1PV. The custom code for evidence accumulation tasks is deposited under the following: https://github.com/NabbefeldG/MS_task_gerion_V2_5.
