# Supplementary material for: DNMT1 function in cortical PV interneurons modulates cortical network activity, mood disorder‑related behavior, and perineuronal nets
Source: Commun Biol. 2026 Oct 8;9:1316. doi: 10.1038/s42003-026-11079-x (PMC13650159; doi:10.1038/s42003-026-11079-x)
Supplement: Supplementary file 1 [file 42003_2026_11079_MOESM1_ESM.pdf]

nature portfolio

Peer Review File

DNMT1 function in cortical PV interneurons modulates cortical network activity, mood disorder-related behavior, and perineuronal nets

Corresponding Author: Professor Geraldine Zimmer-Bensch

This file contains all reviewer reports in order by version, followed by all author rebuttals in order by version.

Version 0:

Reviewer comments:

Reviewer #1

(Remarks to the Author)
In Linde et al., the authors employ the PV-Dnmt1 deletion mouse model, a model that they have previously characterized in terms of PV synaptic function and aging, to study the functional consequences of Dnmt1 specific deletion in PV neurons at the network-level and in behavior. They further perform single-nucleus transcriptomic analysis to suggest a potential molecular mechanism underlying the electrophysiology and behavioral deficits they report, at the system-level.
The authors provide convincing and robust evidence for network desynchronization that results from the PV compromised function.
The authors perform a battery of behavioral tests, all well-justified, and they found that the PV-Dnmt1mice show anxiety and depression like behaviors. We believe that this analysis includes mice form both genders, since in the interaction test and female urine test, the gender of the mice used is obviously defined. Anxiety and depression have a very strong and well-documented gender component. We suggest that the authors should analyze their results, taking this parameter into consideration.
Concerning the snRNA-seq experiment, the authors should provide information about the number of samples and nuclei per sample that have been sequenced.
In the DEG analysis of pyramidal neurons of layers 4-6, log2FC cut-off is 0.26? This is rather low. Can the authors elaborate on this?
Although the focus of the analysis is on other cortical cell populations, can the authors identify PV sub-types in their transcriptomic analysis, and if so, do they have information whether specific PV-types are affected? Also, in the bulk RNAseq experiment they have performed for PV neurons in their previous publication, have they identified any DEGs related to ECM remodeling and PNN assembly/function?
The figure that corresponds to the PV-OPC interaction (line 302) is 4e and not 4d.
Overall, we believe that CellChat analysis does not provide robust evidence for PV-Glia coupling deficits that explain the electrophysiological and behavioral phenotypes described in this work. It is a very interesting finding that needs additional experiments to be verified. We suggest the authors should not over emphasize this statement in the document and we would also suggest that the title should change.

Reviewer #2

(Remarks to the Author)
This manuscript from Linde and colleagues focuses on the role of the DNA methyltransferase 1 (DNMT1) in the function of cortical parvalbumin-expressing (PV) interneurons in behavioral outcomes relevant to depression and anxiety using a PV-specific Dnmt1 deletion mouse model. The authors integrate neuropixels recording cross cortical areas, behavioral readouts, single-nucleus transcriptomics, and histological analyses to show that Dnmt1 deletion in PV-interneurons increases PV spiking activity but reduces inhibitory efficacy and leads to behavioral abnormalities.

The study is interesting and adds significant information on the role of an epigenetic regulator in interneurons function and circuits. However, I have concerns about the robustness of the transcriptomic analyses (and thus of the conclusions drawn) and some other questions about the presentation and statistical interpretation of the data.

Figure 1. While the logic of this experiment is introduced based on the previous findings, the rationale for the experimental design and the design itself are minimally explained. Related to this paragraph, the introduction and discussion of data Extended Figure 2 is limited to a couple of sentences, but there are several questions about these data. The sample size in the visual evidence accumulation task seems underpowered and it is unclear what statistics were performed, because it would seem (based on panels f and g) that the KO mice might be underperforming. Also, the legend for Figure 1 indicates that the data are based on n = 8 recordings from 2 mice per genotype. How have the authors dealt with the nested statistics here?

The description/interpretation of Extended Data Tables 1 & 2 is minimal ("Bulk RNA sequencing also revealed transcriptional changes in Pvalb-Cre/Dnmt1 KO mice resembling those in models of depression and anxiety"). Key information seems missing. Is this bulk RNAseq data newly generated here or part of a prior study? In Extended Data Tables 1 & 2, there should be a reference for each of the mouse models listed. Also, how were enrichment analyses performed? Number of DEG in each of the study and % overlap with the DEG in the Pvalb-Cre/Dnmt1 KO mice should be indicated.

The methods indicate that "Transgenic Pvalb-Cre/tdTomato/Dnmt1 loxP-mice were used as knockout animals and Pvalb-Cre/tdTomato-mice as controls". Please clarify if these are obtained as littermates or through separate breedings. The PV-specific conditional Dnmt1 knockout mouse line is used based on prior work, but it would be nevertheless useful to reiterate in this manuscript the temporal and spatial pattern of Cre expression in the Pvalb-Cre.

Figure 2. I have several concerns about the statistical treatment of the data. The legend for panels a-b indicates that n = 6 mice per genotype were used for the analyses of spontaneous activity within the home-cage, and yet in panel b more data points per genotype are shown. If these are 30-min intervals, they should averaged for each subject or could be represented longitudinally, but cannot be treated as independent data points because the resulting statistics is incorrect. Same concern for panels c & d. The legend reads that the data refers to n(Control) = 13 and n(Knockout) = 11, but more data points are shown in the plot. Are the measures in the 3 consecutive trials? If so, they should be shown as such and a repeated measure test accounting for genotype and trial (and their interaction) should be used. The authors interpret the collective data in Figure 2 as "Pvalb-Cre/tdTomato/Dnmt1 loxP2 (KO) mice display apathetic behavior". For the "coat state" assessment, the age range is 2-7 months. Could this be skewing results (more older animals in the KO group)?

Extended Data Figure 3, panel d. The legend reads "Blood levels of various appetite-regulating hormones were unaltered by the Dnmt1-knockout (n(Control) = 19, n(Knockout) = 16)" but the data points per hormone seem lower than indicated. Please clarify.

Figure 3a. In the lower panel, please add the SEM to the mean running time averaged per genotype in the lower panel so that the reader can assess intra-genotype variance. Was there statistical testing performed here?

For the single-nuclues RNAseq data, the Methods read: "For each genotype, tissue from the three mice was pooled prior to nuclei extraction to obtain sufficient material and minimize inter-individual variability". This is problematic if after pooling the biological samples are n=1/genotype, because it is hard to assess that the differences seen are driven by genotype rather than variability between two samples in the workflow. This might be driving the rather profound differences in the representations of certain populations, as shown in Extended Data Figure 7, especially considering that this is a conditional mouse model.
Do the authors see reduced expression of Dnmt1 as expected in PV interneurons? Also, were the authors able to previously reported Dnmt1 targets?
The methods also indicate that "the top 50 upregulated and top 50 downregulated marker genes per cluster were cross-referenced with established cell type-specific markers from published literature and publicly available reference databases" without providing references for the publications that were used for annotations of clusters.
All in all, the conclusion that "single-nucleus transcriptomics reveals non–cell-autonomous glial remodeling" is not entirely supported by the current data.

The volcano plot in Extended Data Figure 8 is puzzling (distribution of the non-significant genes). Please explain.

Lines 196-199. Please briefly introduce neuronal populations and subcortical regions regulating feeding investigated in Extended Data Figure 4-5 for readers who are not familiar with these circuits.

Figure 6. Please show individual data points for plots in panels e-f. The legend indicates that these are "N = 3 biological replicates with n = 3 slices each per group" and "N = 3 biological replicates with n = 8 slices per genotype". Have values across slices averaged or have the authors conducted a nested analysis?

Reviewer #3

(Remarks to the Author)
There is growing evidence that perturbation of epigenetic processes can be associated with a variety of neurodevelopmental disorders. The authors previously demonstrated that loss of the DNA methyltransferase 1 (DNMT1) in PV+ interneurons increases their mIPSC frequency, and bulk sequencing of PV+ cells revealed that this was in part due to alterations in endocytosis and vesicle replacement (PMID: 32147726). Here the authors expand on these observations to with additional electrophysiology, behavior and single cell sequencing experiments to reveal an array of deficits when DNMT1 is removed

from PV+ interneurons. Overall this manuscript provides interesting insights regarding DNMT1 function in PV+ cells and cortical circuits. But I am somewhat underwhelmed by the conclusions from their single cell sequencing experiments, which is due in part to insufficient methodological details about cell numbers & definitions of cell subtypes, and lack of confirmation of predicted gene expression changes. My specific comments are below.

Major points

1. The authors need to provide additional details about their single cell sequencing methodology & results. It is standard to report number of cells sequenced for each bio rep (WT and mutant), average reads/cell, genes/cell, etc. either in the methods or as a supplementary table. Similarly, for all bioinformatic analysis between 2 tissues/cell types (such as the volcano plots in Fig 4C), the authors should provide a Supplementary Table or source file with the log fold change & adjusted p-value for every gene.

2. I'm concerned about the reliability of some of the author's conclusions about proportional changes from the single cell sequencing data because of lack of power (n = 1 biological rep) & unknown cell numbers in some clusters (as noted above). I am not a bioinformatician, but I believe that adjusted p-values (rather than p-values) should be reported for the 2-sample proportion test, and it's not clear from me in the legends or methods which value was reported. And it seems like a potential over-interpretation of the data for clusters with very small cell numbers (endothelial cells & corticospinal neurons, for example). The authors should consider applying tools like DA-seq (PMID: 34001664) that account for the nuances of snRNA-seq data. Or alternatively, the authors should perform immunostaining with cell counts to confirm that some of these reported proportional differences of cell types between WT and KO mice are confirmed in biological samples. If the authors truly believe that there is a 3-4 fold change in some cell types with very low cell numbers, as opposed to the expected biological 'noise' in these types of experiments, then that should be easy to demonstrate via immunos (or FISH).

3. A significant weakness is that the authors do not perform any validation of predicted DEGs from their single cell sequencing dataset. This is especially problematic considering my concerns above about the power of these analyses due to low cell numbers of some clusters. The authors identified several ligand-receptor pairs between PV+ cells and oligos that were significantly up- or down-regulated in KO mice (Figure 5D-E). At a minimum, the authors should perform in situ hybridizations or immunohistochemistry to confirm differential expression of some of these ligand-receptor cells in PV+ cells and oligos. Showing loss of PNNs with WFA staining is nice & consistent with the e-phys deficits, but it does not directly address any of the putative DEGs from their scRNAseq dataset.

4. The authors inconsistent classification of oligodendrocytes is confusing and it's not clear to me what their basis is for defining these subpopulations. In Fig 4, there are 3 classes defined as OPCs, newly mature oligos and myelinating oligos. I do not know what the term 'newly mature' means, is that newborn immature/non-myelinating oligos? I'd recommend renaming. Based on the Fig 4A UMAP, it appears that these 'new oligos' and 'myelinating oligos' are salt-&-pepper in the same cluster. What is the basis for differentiating these 2 types of oligos? The gene expression and heatmaps in Fig 4B & 4E, respectively, are basically indistinguishable, so I don't understand this classification. I strongly recommend just combining these 2 subgroups as 'oligodendrocytes' unless they can justify this distinction. Then later in the results the authors add a fourth group 'perineuronal oligos' and include this in Fig 5 analyses. Again I have no idea what genes distinguishes this subset from other subset, which cluster/subtype they arise from, why it was only analyzed in Fig 5 and not Fig 4, etc. And to reiterate my earlier concerns, the more you subcluster the data, the fewer cells & less power the analyses have, which is why you get a volcano plot like Ext Data 8 where there are so few DEGs and all genes have an adj p-value of 0. In my opinion, the authors need to redo this oligo analyses by clearly defining these 3-4 subtypes of oligos in the beginning (based on gene expression and/or mapping them onto reference databases) and use these subtypes in their analysis in Fig 4 & 5. Or alternatively, the authors should just use 'OPCs' and 'mature oligos' for their downstream analyses. And the authors should generate Oligo-only UMAPs with these subtypes clearly labeled (and ideally with a companion WT/KO labeled UMAP). As it stands, I really don't know what to make of this entire oligo analyses for all these reasons stated above.

5. The authors show that there is no difference in PV cell numbers in the hypothalamus (Ex Data 4), which agrees with their previous report of no differences in PV+ cortical interneurons in DNMT1 mutant mice (PMID: 32793592). They then claim that PV cell identity is "unaltered in [knockout] mice" (title of Ext Data Fig 5) simply because beta-endorphin, a marker of POMC cells, does not overlap with PV immunos. Based on this observation, the authors claim that it is "…unlikely that Dnmt1 deletion in PV cells directly interferes with hypothalamic feeding circuits" (lines 199-200). I do not agree with this conclusion for several reasons. First, just because a single marker does not overlap with PV cells in WT and mutant mice does not justify the conclusion that PV+ cells in the hypothalamus are unaltered. Second, the authors clearly demonstrate (and spend a significant amount of time discussing) the non-cell autonomous changes in genes expression of cortical cells when Dnmt1 is removed only in cortical PV+ interneurons. Why do the authors not think the same type of non-cell autonomous effects occur within the hypothalamic feeding circuit? My assumption is that performing single cell sequencing on hypothalamic cells would likely reveal non-cell autonomous DEGs in these cells from loss of Dnmt1 in PV+ cells, just like in the cortex. Third, while only leptin levels reached statistical significance, there certainly is a strong trend for increased ghrelin and GLP-1 levels in the mutant mice (Ext Data 3). Thus, the authors justification that the hypothalamic feeding circuit is not altered in Dmnt1 KO mice is not supported by their data; they have not performed a similar detailed analysis as they did in the cortex to justify this statement. Relatedly, I do not believe the authors have adequately shown that the changes in weight, hormone levels, etc., are not due to hypothalamic dysfunction and instead are solely due to an apathetic/anhodenic state as the authors claim. Instead, it seems reasonable (and this reviewer would argue likely) that some alteration in hypothalamic/feeding circuitry in part underlies the effects.

6. I was somewhat confused about why the authors focused so heavily on the communication between PV cells and oligos. The authors state that PNNs require continuous support from oligos (lines 296-297), but there are no citations here and I am not familiar with this concept in the literature. Please provide additional references & context supporting the idea that oligodendrocyte-lineage cells play an important role in the formation and/or maintenance of PNNs, and thus PV cell function.

Minor points

7. I don't understand the point of Extended Data Tables #1 & 2, nor do I understand what they are displaying. The meaning of the single sentence in the text referencing this tables (lines 180-182) is unclear to me, and the Table legends don't provide any additional insight. I don't know what any of the columns in the table are referring to: where do these FDR, gene numbers & fold enrichment come from? There are no citations for any of the manuscripts/mouse lines that are referred to in the table. I recommend that the authors remove these tables as their purpose here is unclear & confusing.

8. In several graphs the y-axis does not start at 0 (Fig 2e, 6e, Ext Data Figs 1a, 2b, 2j, 3a-c, 6a) which can lead to confusion when comparing graphs. Please ensure that all relevant graphs start at the y = 0 intercept.

9. Figure 1i: Please label each heatmap as Cont/KO in the figure.

10. Figure 4a: The authors need to show a UMAP color coded by WT and KO samples, which would be helpful for visualizing similarities (and differences) between the conditions. Are the WT & KO cells intermingled with each other (i.e., salt & pepper), or is there some separation between WT and KO cells within the clusters?

11. Extended Data Figure 4a: I don't understand why the authors overlayed the raw WT (pseudocolored green) and KO (red) tom+ cells in the right column. It is somewhat distracting because you can clearly see that the WT and Hom sections are not perfectly matched along the A-P axis, most notable in the second & third images. I cannot see the individual Tom+ cells in these small low mag images, so maybe replace these images with fewer sections along the A-P axis, allowing for larger images with greater resolution to see individual cells?

12. I find Figure 5D-E confusing to read. The left & right columns are not labeled in the figure panels; the PV-to-Olig direction should be clearly labeled in the figure. Also, it's not clear to me why the up and down-regulated pairs are separated into two panels. It makes more sense to have a single panel with upregulated-downregulated pairs for each direction on a sliding red-blue shaded scale, just like Fig 4E; that is a more standard way to present this type of heatmap. Are any of these predicted differences significant? The delta values look pretty small (highest is maybe 0.15), so is this a statistically significant difference?

13. Figure 4A: What does the term 'Atypical excitatory neurons' mean? Is this taken from a previous publication, or just a term to describe cells that don't map onto other defined excitatory populations? They also show the most consistent and unidirectional change in the Fig 4E heatmap (whole row is blue), so some clarification in understanding this population would be helpful.

14. Extended Data 2d: I don't understand how the y-axis 'Path efficiency' is defined. The legend says that it's swimming trial distance divided by shortest route, which should always be a positive number > 1.0 since the swimming distance is always larger than shortest route. So I'm not sure why the y-axis ranges from 0-1, maybe there is some normalization factor after calculating this distance?

15. I am not a behavior expert, can the authors please clarify which assays test apathy (lack of motivation) vs. anhedonia (lack of feeling pleasure), if that distinction is well established in the field. At times it wasn't clear to me which behavior is associated with which of these terms.

16. Line 321-323: The authors stated that NRG1-ERBB4 from OPCs to PV cells is significantly diminished, but doesn't Fig 5E show that this is significantly increased? And how does this change the reference/citation about loss of trophic support?

17. The authors analyzed e-phys properties from V1, S1 and ALM to demonstrate that changes were not restricted to a single cortical area. But they only reported changes in PNNs in S1. Can the authors confirm that decreased PNNs/WFA staining around PV cells is also present in other cortical regions such as V1 and ALM?

18. I think the title is somewhat misleading since the authors haven't directly tested or shown that changes in the PV interneuron-glia coupling underlies these deficits, especially without confirming any of the DEGs or receptor-ligand changes.

Version 1:

Reviewer comments:

Reviewer #1

(Remarks to the Author)
The authors have adequately addressed reviewers' comments and provided substantial clarifications regarding both methodological approaches and data interpretation.
However, in the revised manuscript they considerably expand the results concerning the non-cell-autonomous

consequences of PV-specific DNMT1 deletion on excitatory neuronal populations. Specifically, they report an apparent overrepresentation of layer 4 pyramidal neurons and Tox+/Slc17a6+ corticospinal populations in KO mice.
These observations warrant additional discussion. In particular, it should be clarified in the manuscript why the enrichment of Tox+/Slc17a6+ populations most plausibly reflect activity- or circuit-dependent state transitions induced by altered inhibitory function, rather than expansion of a stable excitatory neuronal subtype (Artegiani et al., 2014:calcineurin/Nfat/Tox; Sahni et al., 2021:early specification of corticospinal neurons). Line: 463-464 "Single-nucleus RNA-seq uncovered an altered excitatory neuron composition" this is a confusing statement. Similarly, the downregulation of expression levels of activity dependent genes (Arc, c-Fos etc) should be discussed. There are several papers which propose that chronic network hyperactivity and other persistent activity paradigms result in sustained elevations in activity, which can lead to reduced activity-dependent gene expression (Corbett et al.,2018).
Overall, the study provides meaningful insights and represents a valuable contribution to the field. I therefore support publication.

Reviewer #2

(Remarks to the Author)
The authors have satisfactorily addressed my comments.

Reviewer #3

(Remarks to the Author)
In this revised manuscript, the authors have made significant improvements that address many of the reviewer's comments. They have increased the methodological details & and included extended data files regarding their animal numbers, use of statistics & scRNAseq data that now allow reviewers/readers to more fully understand their analyses. The use of Milo to quantify proportional changes in cell types between WT and KO mice is a welcome addition. They have clarified the oligo nomenclature & how they define different subtypes. And I am satisfied with the authors explanations regarding hypothalamic function in the KO mice & their conclusion that changes in the hypothalamic PV+ cells & specific hypothalamic nuclei likely play a minor role at best in the observed phenotypes.

However, I still feel that there are important issues that were not adequately addressed by the authors' comments & manuscript changes that still have me question some of their conclusions.

Major points

1. A significant weakness of the manuscript continues to be that the authors still have not validated any of the putative proportional cell changes or DEGs in the manuscript. The change in PNN is a nice observation, but it does not directly address or confirm any of these predicted changes. And the addition of Milo analysis supports some of their conclusions with the proportional cell changes, but it is basically an additional (albeit valuable) way to reanalyze the same dataset. Without any validation of these predicted cellular and/or genetic changes, the manuscript in its current version is too speculative (especially extending to CellChat analyses). The authors cited several reasons for not validating any predicted changes, but I disagree with some of their points outlined below:

a. I initially suggested that the authors validate the strong 3-4 fold increase in corticospinal neurons, as that large a change should be readily apparent via FISH or immuno. While Milo confirmed this change & is a welcome addition, it is still essentially a reanalysis of the same dataset. In response, the authors stated that establishing multiplexed FISH panels lies beyond the scope of the present study, and combining multiple markers makes panel design technically challenging. However, the authors clearly state in the revised manuscript that these corticospinal neurons are "…defined by the expression of Tox and Slc17a6 and the absence of Tle4 and Cux2…" (lines 283-284). Thus, there is a clear strategy to identify these cell types via FISH. It is not too difficult to perform FISH using 2-4 probes to confirm such a large predicted change in the proportion of cells between WT and KO mice.

b. There are also good markers that can be used for immunohistochemistry and/or FISH to confirm significant proportional changes in Layer 2-3 IT cells, Layer 4 cells & Layer 5-6 cells (Ext Data 7b). The authors find a significant decrease in astrocytes in KO mice, which can easily be assessed with GFAP immunos. I don't necessarily expect the authors to perform all of these suggestions, but there are well-established markers for many of these cell types that can be used for FISH and/or immunos. None of this seems beyond the scope of this study; in fact, validation of these predictions is critical to ensure confidence in the predicted cellular & DEG changes.

c. I also previously noted that the authors did not perform any validation of predicted DEGs from their scRNAseq dataset, and they still do not address this. In their response, the authors claim that the WFA staining "…directly confirms the biological endpoint of this signaling reorganization…", but that is extremely speculative that PNN changes are a "biological endpoint", and I would argue not a clear justifiable conclusion from their data. The authors make several strong claims in the revised manuscript about DEGs in the scRNAseq dataset that can easily be confirmed. To note a few, they claim broad upregulation of Fkpb5 and Zbtb16 in many cell types (lines 306-311), downregulation of Arc, Fos & Nra1-3 (lines 314-317), and a reduction of Pdgfc+ oligos (lines 367-370). Yet there is no orthogonal validation of any of these claims. There are very good antibodies for Arc and Fos that work for immunostaining, and upregulation of Fkbp5 and Zbtb16 in so many cell types should be very apparent with FISH. Again, I'm not expecting the authors to confirm every DEG, but they should validate some of these predictions via FISH and/or immunos, especially ones that are critical for additional analyses or important

conclusions.

2. I had numerous criticisms of the CellChat analyses in my original response, and Reviewer #1 had similar concerns stating "…CellChat analysis does not provide robust evidence for PV-glia coupling deficits… it is a very interesting finding that needs additional experiments to be verified…the authors should not over emphasize this statement". The authors' response of adapting the description & changing the title does not address either Reviewer #1 or my significant concerns about this CellChat analyses.

a. I will reemphasize that the authors did not provide any validation of these changes of signaling pathways genes, which both myself and Reviewer #1 requested/suggested. The authors' response is that the CellChat analyses "…are not intended to identify specific DEGs… rather to characterize shifts in predicted communication probabilities…". This statement is very odd as the whole CellChat analyses is based on differential expression of these signaling pathway genes between cell types & genotypes determined from their scRNAseq datasets (unless I'm misunderstanding this analysis works). This point only further emphasizes the need to confirm some of the predicted DEGs via FISH. Without some sort of confirmation, this entire CellChat analysis is purely speculative, and I agree with Reviewer #1 that it does not provide robust evidence for PV-glia coupling deficits. The authors claim in their rebuttal to this point that "We therefore consider the WFA data to be an appropriate functional validation of the overall conclusion", which is entirely speculative and not a valid statement. To conclude that WFA changes are a direct result and/or logical conclusion from the DEGs and CellChat analyses does not make sense to this reviewer.

b. I would argue that the revised manuscript now puts additional emphasis on this CellChat analyses and contains mischaracterizations/inaccuracies of the CellChat data that are not justified. In discussing the CellChat analyses (lines 327-382), the authors use terms like "reduction in neuron-to-neuron communication…reductions in outgoing/incoming communication/signaling…suppression of neuronal signaling…marked reduction of SST+ input onto PV cells…loss of excitatory/inhibitory inputs…functional uncoupling of PV-OPC axis". The last 2 paragraphs (lines 383-402) talk about Nrg1-Erbbr and GABA signaling being reduced/increased even though there is no evidence for this. Any conclusion about altered signaling, changes in communication, reductions/increases of inputs, etc., are entirely speculative. Without any orthogonal validation, the authors should not conclude that differential mRNA levels of these receptor-ligand pairs between cell types & genotypes definitively leads to changes in cell communication, signal strengths, changes in inputs, etc. Even if some of these putative changes are consistent with their optogenetic data, it is not valid to assume that these genetic changes definitively give rise to the altered signaling pathways they claim here (especially since we don't even know if these mRNA changes drive different protein levels). Thus, rather than de-emphasizing this CellChat analyses, the authors appear to have significantly increased their interpretation of this analysis. I do not believe this is justified without additional validation, and I do not believe that the authors have revised their manuscript in a way that accurately reflects the significant weaknesses and speculative nature of this analysis; rather, they have gone in the opposite direction.

3. The authors note that Pdgfc is a defining marker for the perineuronal oligodendrocytes, but they don't include this gene in the Fig 4B dot plot. Please add this gene to this panel to demonstrate that it demarcates this population. Additionally, I'd recommend generating oligo UMAP plots highlighting expression of Enpp6 and Pdgfc and including them in Ext Data Fig 8, this should nicely highlight the differential expression of these genes in oligo subtypes. I also think that the UMAP with the 4 defined oligo populations in their Response should be included in the manuscript, probably in Ext Data Fig 8. I'm somewhat skeptical that these perineuronal oligos represent a real oligo subtype since they are scattered throughout the UMAP (orange dots) and don't form any type of clear cluster, but hopefully visualizing expression of these marker genes will alleviate this concern.

Minor points

4. I had previously asked that the authors provide a citation(s) for their claim that "PNNs require continuous support from the oligodendrocyte lineage…" (lines 355-356), as this seems like an important claim to justify their analyses with oligo-PV communication. Despite the authors' response that they added references to address this, this line is still not cited in the revised manuscript. Please provide a citation supporting this claim.

5. I appreciate that the authors added the requested UMAPs defined by WT and KO in Extended Data Fig 7. However, to better assess if the WT and KO cells are intermingled (i.e., salt & pepper) or there is spatial biases between cell types in specific clusters, the authors should label WT and KO cells in the same UMAP (just as they did with the Oligo UMAPs in Ext Data Fig 8) rather than separate the 2 genotypes into 2 separate UMAPs.

6. I was unclear about which cell populations were significantly different in the Milo analyses. Were the same 8 cell populations that were significant in the proportionality test (Ext Data Fig 7A) also significant in Milo? Did the astrocyte change reach significance in Milo? Is every cell type represented by a dot in panel D significant? A clear statement about which cell types reached significance in Milo is needed.

7. While the authors have clarified what Ext Data Table 1 & 2 is showing, I still don't really understand the purpose of these tables. The authors claim that DEGs in their mice "…are significantly enriched in transcriptional signatures from mouse models displaying depression- and anxiety-like behaviors" (lines 189-192). However, there seems to be no difference in the % of genes, FDR or fold enrichment between mouse models that display depression/anxiety (marked with asterisk) vs. mouse lines that don't display depression/anxiety. Doesn't this actually refute the authors point, if many of these genes are up/downregulated in numerous mouse models that don't display these phenotypes? Thus, I don't agree with the authors statement above. Or at least it should be noted that the genes are also significantly enriched in many mutant mouse models

that don’t display these phenotypes, which again undercuts the authors’ argument here. I still recommend that the authors remove these tables because I don’t think they add anything to their story. If for some reason the authors do want to keep these tables, each mouse line needs to be cited with the relevant paper accordingly.

Version 2:

Reviewer comments:

Reviewer #1

(Remarks to the Author)
The authors have adequately addressed all the points raised during the previous round of review, and the manuscript has been significantly improved. Only a few minor issues remain, which should be straightforward to address:
1. In Figure 7a,b, Olig2 (magenta) cannot be clearly distinguished from tdTomato (red). Please use more distinct pseudocolors for these two signals.
2. In Supplementary Figure 7, the most prominent GFAP immunoreactivity appears to originate from fibrous astrocytes, in which GFAP is strongly expressed. By contrast, the weaker GFAP signal from astrocytes throughout the cortical layers is not readily discernible. If images at this magnification were used to quantify GFAP density, the measurements may be dominated by strongly labelled fibrous astrocytes and may not accurately represent GFAP expression within the cortex. Please perform or present the analysis using higher-magnification, region-specific images, or provide higher-magnification representative images that allow the cortical staining to be properly evaluated.
3. In Supplementary Figure 11, the tdTomato signal does not appear representative of PV interneuron labelling. Almost the entire section exhibits strong red fluorescence, which cannot reasonably be attributed solely to the somata and processes of PV neurons. Please replace this image with a more representative, higher-magnification image in which tdTomato-positive PV interneurons can be clearly identified.
More generally, the newly added immunohistochemical findings would be presented more convincingly using higher-magnification images, similar to those shown in Figure 7d,e.
Subject to these minor revisions, I endorse the manuscript for publication.

Reviewer #3

(Remarks to the Author)
The authors have done a good job addressing the reviewers’ comments and improving the manuscript. They provide new immunohistochemistry experiments to confirm some of their initial observations, and their new scATAC data with Regulon/Scenic+ analyses provides additional support for many of their original insights about changes in mRNA levels and predicted alterations of cell-cell communications. They have satisfied my other concerns and adjusted portions of the manuscript to more accurately portray the speculative nature and/or hypothesis-generating aspects of the analyses. I think this work is an important contribution to the field and I have no further comments.

Version 3:

Reviewer comments:

Reviewer #1

(Remarks to the Author)
The figures have been significantly improved. I am happy to endorse this work for publication.

Open Access This Peer Review File is licensed under a Creative Commons Attribution 4.0 International License, which permits use, sharing, adaptation, distribution and reproduction in any medium or format, as long as you give appropriate credit to the original author(s) and the source, provide a link to the Creative Commons license, and indicate if changes were made.
In cases where reviewers are anonymous, credit should be given to 'Anonymous Referee' and the source.
The images or other third party material in this Peer Review File are included in the article's Creative Commons license, unless indicated otherwise in a credit line to the material. If material is not included in the article's Creative Commons license and your intended use is not permitted by statutory regulation or exceeds the permitted use, you will need to obtain permission directly from the copyright holder.
To view a copy of this license, visit https://creativecommons.org/licenses/by/4.0/

Reviewer #1

In Linde et al., the authors employ the PV-Dnmt1 deletion mouse model, a model that they have previously characterized in terms of PV synaptic function and aging, to study the functional consequences of Dnmt1 specific deletion in PV neurons at the network-level and in behavior. They further perform single-nucleus transcriptomic analysis to suggest a potential molecular mechanism underlying the electrophysiology and behavioral deficits they report, at the system-level.
The authors provide convincing and robust evidence for network desynchronization that results from the PV compromised function.
The authors perform a battery of behavioral tests, all well-justified, and they found that the PV-Dnmt1mice show anxiety and depression like behaviors.

1. We believe that this analysis includes mice from both genders, since in the interaction test and female urine test, the gender of the mice used is obviously defined. Anxiety and depression have a very strong and well-documented gender component. We suggest that the authors should analyze their results, taking this parameter into consideration.

   We appreciate the importance of this consideration, particularly given the well-established sex differences in anxiety- and depression-related phenotypes.

   We would like to clarify that all experiments in the present study were conducted exclusively in male mice, as stated in the methods section 'Animals'. Our decision to use only male animals was made to be in compliance with previous findings: in Pensold et al. (2020) our group used mice of both sexes for the in vitro electrophysiology assessments but only male mice for RNA and MeDIP sequencing. As our aim for the present study was to extend our knowledge about the effects of DNMT1 in interneurons on cortical cells and networks and to meaningfully connect findings on these scales with behavioral observations, we chose to focus on a single sex to establish a clear and internally consistent dataset.

   While sex-specific differences in neuropsychiatric disorders are highly important, incorporating both sexes would require substantially larger, appropriately powered cohorts and additional experimental controls (e.g., estrous cycle monitoring) to ensure rigorous interpretation. We fully agree that extending these findings to females is an important direction for future work and will help determine the generalizability of the observed effects. But for the submitted study, this is beyond the scope and what is manageable.

2. Concerning the snRNA-seq experiment, the authors should provide information about the number of samples and nuclei per sample that have been sequenced.

   We have provided the relevant information in the methods section.

3. In the DEG analysis of pyramidal neurons of layers 4-6, log2FC cut-off is 0.26? This is rather low. Can the authors elaborate on this?

   We thank the reviewer for raising this point and appreciate the opportunity to clarify our rationale. A log2FC cut-off of 0.26 (corresponding to an approximately 20% fold change) is close to the established default threshold of 0.25 in the widely adopted Seurat framework for single-cell differential expression analysis (Stuart et al., 2019). This choice is deliberately more permissive than typical bulk RNA-seq thresholds for several reasons:
   First, single-nucleus RNA-seq captures only nuclear transcripts, which systematically attenuates detected fold changes compared to whole-cell approaches.
   Second, cortical cell types such as excitatory neurons contain diverse transcriptional sub-populations, and averaging expression across heterogeneous nuclei within a cluster further compresses effect sizes. Third, our analysis couples this log2FC threshold with a stringent adjusted p-value cut-off (FDR < 0.05), meaning only statistically robust changes are reported regardless of their magnitude. This combination of a permissive effect-size filter with a strict significance filter is a community-standard practice for snRNA-seq DEG analyses (Luecken & Theis, 2019; Ko et al., 2023).

   Importantly, we do not interpret the specific magnitude of fold change as evidence of strong or weak regulation, but rather use the DEG list to assess pathway enrichment (GO term analysis), where the directionality matters more than individual fold-change values.

   Stuart, T., Butler, A., Hoffman, P., et al. (2019). Comprehensive integration of single-cell data. Cell, 177(7), 1888–1902.

   Luecken, M.D. & Theis, F.J. (2019). Current best practices in single-cell RNA-seq analysis: a tutorial. Molecular Systems Biology, 15(6), e8746.

Ko, K.D. et al. (2023). Integrating single-cell transcriptomes, chromatin accessibility and gene regulatory networks. PMC, 10199178.

4. Although the focus of the analysis is on other cortical cell populations, can the authors identify PV sub-types in their transcriptomic analysis, and if so, do they have information whether specific PV-types are affected? Also, in the bulk RNAseq experiment they have performed for PV neurons in their previous publication, have they identified any DEGs related to ECM remodeling and PNN assembly/function?

   We thank the reviewer for their comment. If we increase the resolution, we do indeed see a subclustering of PV interneurons. However, between our control and KO samples, we do not observe any statistically significant changes in the PV subpopulations neither when normalized to *Pvalb*+ nuclei numbers nor to total number of nuclei per genotype.

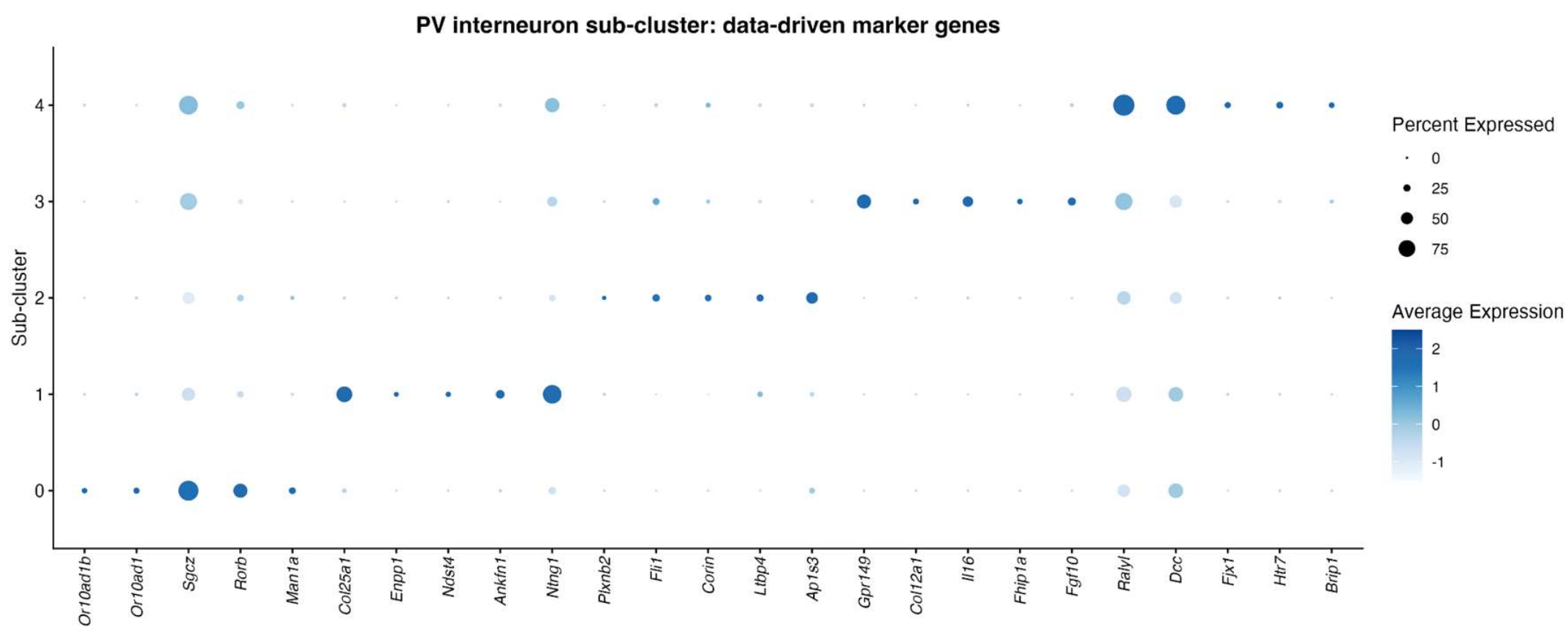

Cluster	RelativeDiff_withinPV	adj_p_value_withinPV	RelativeDiff_ofTotal	adj_p_value_ofTotal
0	10.3433218710769	0.486480816792947	21.2647449812375	0.294128688743892
1	33.4910013844024	0.246970806882384	46.703506525597	0.226284748040758
2	-30.1592858393138	0.197420622179363	-23.2466791067031	0.294128688743892
3	6.3836332871866	0.857562897665463	16.9131393000926	0.552287146034331
4	-23.6040609137056	0.400612074302914	-16.0426393386481	0.552287146034331

5. The figure that corresponds to the PV-OPC interaction (line 302) is 4e and not 4d.

   We thank the reviewer for this remark, we corrected the respective reference.

6. Overall, we believe that CellChat analysis does not provide robust evidence for PV-Glia coupling deficits that explain the electrophysiological and behavioral phenotypes described in this work. It is a very interesting finding that needs additional experiments to be verified. We suggest the authors should not over emphasize this statement in the document and we would also suggest that the title should change.

   We have adapted the way we describe the results and changed the title of the manuscript accordingly.

Reviewer #2

This manuscript from Linde and colleagues focuses on the role of the DNA methyltransferase 1 (DNMT1) in the function of cortical parvalbumin-expressing (PV) interneurons in behavioral outcomes relevant to depression and anxiety using a PV-specific Dnmt1 deletion mouse model. The authors integrate neuropixels recording cross cortical areas, behavioral readouts, single-nucleus transcriptomics, and histological analyses to show that Dnmt1 deletion in PV-interneurons increases PV spiking activity but reduces inhibitory efficacy and leads to behavioral abnormalities.

The study is interesting and adds significant information on the role of an epigenetic regulator in interneurons function and circuits. However, I have concerns about the robustness of the transcriptomic analyses (and thus of the conclusions drawn) and some other questions about the presentation and statistical interpretation of the data.

1. Figure 1. While the logic of this experiment is introduced based on the previous findings, the rationale for the experimental design and the design itself are minimally explained.

   To examine network-level consequences of Dnmt1 deletion in PV interneurons in vivo, we required an approach to simultaneously capture large-scale cortical population activity and selectively identify PV interneurons within the recorded neural population. We therefore used high-density Neuropixels recordings in a head-fixed setup combined with optogenetic tagging of ChR2-expressing PV cells while mice were freely running on a wheel. This design further allowed us to compare spontaneous and sensory-evoked activity within the same animals and recording sessions, enabling a direct assessment of whether PV-specific Dnmt1 deletion affects both baseline network dynamics and sensory processing. We have added further explanation in the text (ll. 98ff) to make this point clearer.

2. Related to this paragraph, the introduction and discussion of data Extended Figure 2 is limited to a couple of sentences, but there are several questions about these data. The sample size in the visual evidence accumulation task seems underpowered and it is unclear what statistics were performed, because it would seem (based on panels f and g) that the KO mice might be underperforming.

   To directly address this point, we now include additional behavioral data, increasing the total number of mice to 5 control and 7 knockout animals. Statistical comparisons were performed by pooling recordings across all animals within each training stage (3 sessions per bin) and using a linear mixed-effects (LME) model to account for the nested data structure (see also our response to the next point below). Importantly, by comparing performance levels at each training stage, this analysis directly tests whether the two groups differ in both absolute performance and learning rate and we find no significant difference at any stage. The increased sample size and more rigorous statistics therefore confirmed our initial observation that there is no clear difference in learning between the two groups. We now also report the corresponding sample sizes, LME test statistics, and p-values in the figure caption and have extended the methods accordingly.

3. Also, the legend for Figure 1 indicates that the data are based on n = 8 recordings from 2 mice per genotype. How have the authors dealt with the nested statistics here?

   We thank the reviewer for this important point. As mentioned above, we have now updated these group comparisons and use LME models to determine significant genotype differences while accounting for the nested data structure. This approach ensures that multiple recordings from the same animal are not treated as independent observations, with the genotype effect estimated against the appropriate between-animal variance (Aarts et al., 2014, Nature Neuroscience, 17, 491–496). Statistical significance was then assessed via likelihood ratio tests comparing the full model to a reduced null model. This new approach yielded similar results as in our previous analysis and we have updated the resulting p-values in the figure legend as well as the corresponding methods section.

   Aarts E, Verhage M, Veenvliet JV, Dolan CV, van der Sluis S. A solution to dependency: using multilevel analysis to accommodate nested data. Nat Neurosci. 2014 Apr;17(4):491-6. doi: 10.1038/nn.3648. Epub 2014 Mar 26. PMID: 24671065.

4. The description/interpretation of Extended Data Tables 1 & 2 is minimal ("Bulk RNA sequencing also revealed transcriptional changes in Pvalb-Cre/Dnmt1 KO mice resembling those in models of depression and anxiety"). Key information seems missing. Is this bulk RNAseq data newly generated here or part of a prior study?

The bulk RNA-seq data was generated within the scope of our previous publication (Pensold et al. 2020, Ref. 8 in the manuscript). We have adjusted the phrasing in the manuscript to make it clearer.

5. In Extended Data Tables 1 & 2, there should be a reference for each of the mouse models listed. Also, how were enrichment analyses performed? Number of DEG in each of the study and % overlap with the DEG in the Pvalb-Cre/Dnmt1 KO mice should be indicated.

   We thank the reviewer for this constructive comment. We have revised the tables accordingly, added the GEO accession numbers for the individual datasets as well as the citations for the observed phenotypes.

6. The methods indicate that "Transgenic Pvalb-Cre/tdTomato/Dnmt1 loxP-mice were used as knockout animals and Pvalb-Cre/tdTomato-mice as controls". Please clarify if these are obtained as littermates or through separate breedings.

   Control and knockout mice were obtained through separate breedings, we added this point in the methods.

7. The PV-specific conditional Dnmt1 knockout mouse line is used based on prior work, but it would be nevertheless useful to reiterate in this manuscript the temporal and spatial pattern of Cre expression in the Pvalb-Cre.

   We thank the reviewer for this important remark, as both the temporal and spatial characteristics of *Pvalb* expression were key considerations in the selection of our mouse model and breeding strategy. In the Pvalb-Cre line, Cre recombinase expression is driven by the endogenous *Pvalb* promoter. The onset of *Pvalb* expression in mice occurs during early postnatal development (del Rio et al., 1994) and is regulated by neuronal activity (Sugiyama et al., 2008), thereby linking the maturation of parvalbumin (PV) interneurons to experience-dependent plasticity. This developmental profile makes the Pvalb-Cre model particularly suitable for investigating postnatal effects of *Dnmt1* expression.

   Spatially, parvalbumin is predominantly expressed in GABAergic interneurons across multiple brain regions, including the neocortex, hippocampus, thalamus, striatum, and cerebellum (Meyer et al., 2002), although low-level expression has also been reported in a subset of long-range projecting pyramidal neurons (Baccini et al., 2025). We specifically employed the Pvalb-ires-Cre mouse line (first described by Hippenmeyer et al., 2005), in which Cre activity is largely confined to cells with high *Pvalb* expression, thereby predominantly targeting fast-spiking interneurons (Madisen et al., 2010). To prevent unintended germline recombination—previously reported in males of this line due to *Pvalb* expression in the testis—we used Cre-positive females for breeding in combination with Cre-negative males (Kobayashi & Hensch, 2013).

Baccini, G., Foggetti, A., Wernet, N., Engelhardt, K. A., Steinecke, C. S., Edelmann, E., ... & Wulff, P. (2025). Parvalbumin expression identifies subicular principal cells with high projection specificity. Cell Reports, 44(8).

del Rio, J., De Lecea, L., Ferrer, I., & Soriano, E. (1994). The development of parvalbumin-immunoreactivity in the neocortex of the mouse. Developmental Brain Research, 81(2), 247-259.

Hippenmeyer, S., Vrieseling, E., Sigrist, M., Portmann, T., Laengle, C., Ladle, D. R., & Arber, S. (2005). A developmental switch in the response of DRG neurons to ETS transcription factor signaling. PLoS biology, 3(5), e159.

Kobayashi Y, Hensch TK. Germline recombination by conditional gene targeting with Parvalbumin-Cre lines. Front Neural Circuits. 2013 Oct 16;7:168. doi: 10.3389/fncir.2013.00168. PMID: 24137112; PMCID: PMC3797395.

Madisen L, Zwingman TA, Sunkin SM, Oh SW, Zariwala HA, Gu H, Ng LL, Palmiter RD, Hawrylycz MJ, Jones AR, Lein ES, Zeng H. A robust and high-throughput Cre reporting and characterization system for the whole mouse brain. Nat Neurosci. 2010 Jan;13(1):133-40. doi: 10.1038/nn.2467. Epub 2009 Dec 20. PMID: 20023653; PMCID: PMC2840225.

Meyer AH, Katona I, Blatow M, Rozov A, Monyer H. In vivo labeling of parvalbumin-positive interneurons and analysis of electrical coupling in identified neurons. J Neurosci. 2002 Aug 15;22(16):7055-64. doi: 10.1523/JNEUROSCI.22-16-07055.2002. PMID: 12177202; PMCID: PMC6757887.

Sugiyama, S., Di Nardo, A. A., Aizawa, S., Matsuo, I., Volovitch, M., Prochiantz, A., & Hensch, T. K. (2008). Experience-dependent transfer of Otx2 homeoprotein into the visual cortex activates postnatal plasticity. Cell, 134(3), 508-520.

8. Figure 2. I have several concerns about the statistical treatment of the data. The legend for panels a-b indicates that n = 6 mice per genotype were used for the analyses of spontaneous activity within the home-cage, and yet in panel b more data points per genotype are shown. If these are 30-min intervals, they should averaged for each subject or could be represented longitudinally, but cannot be treated as independent data points because the resulting statistics is incorrect.

   We thank the reviewer for this important remark and corrected our statistical analysis and visual representation. As suggested, we changed panel 2b to a longitudinal representation of activity patterns to more accurately depict the time-independent overall reduction of *Dnmt1*-knockout mice. We then conducted a linear mixed-effects model to account for inter-individual variability as well as fluctuations within and across sessions. This more accurate comparison likewise indicated a significant genotype-dependent difference in uninstructed movement of mice in a familiar environment. We accordingly updated the representation in Fig. 2, the corresponding figure legend and the respective description in the methods section.

9. Same concern for panels 2c & d. The legend reads that the data refers to n(Control) = 13 and n(Knockout) = 11, but more data points are shown in the plot. Are the measures in the 3 consecutive trials? If so, they should be shown as such and a repeated measure test accounting for genotype and trial (and their interaction) should be used.

   We accordingly revised the statistical analysis and visualization. As the reviewer pointed out, we previously displayed all latencies from individual trials of mice performing the rotarod or wire-hang test. We corrected these representations to display only the obtained mean value per animal and task in the main figure (see Fig. 2c and d) and added a representation of the trial-wise average in the extended data set (see Extended Data Fig. 3a and b). This extended visualization supports our finding that there is no apparent difference between control and knockout mice performances in the rotarod test (Fig. 2c, Extended Data Fig. 3a) whereas decreased latencies to fall in the wire-hang test are due to genotype-dependent performance differences (Fig. 2d) and not based on signs of fatigue related to task repetition (Extended Data Fig. 3b). To account for inter-individual differences when comparing performances in both tasks regarding genotype- and trial-specific deviations, we used linear mixed-effects models and could only detect a genotype-dependent difference in the wire-hang test. The revised statistics are noted in the respective figure legends.

10. The authors interpret the collective data in Figure 2 as "Pvalb-Cre/tdTomato/Dnmt1 loxP2 (KO) mice display apathetic behavior". For the "coat state" assessment, the age range is 2-7 months. Could this be skewing results (more older animals in the KO group)?

    The overall age range of animals used for the present study was 3-6 months of age, as mentioned in the chapter "Animals" of the methods section. This range was chosen as there is a broad scientific consensus that this time window represents adulthood in mice (https://www.jax.org/news-and-insights/jax-blog/2017/november/when-are-mice-considered-old?utm_source=chatgpt.com). As the investigated mice for the coat state assessment slightly deviated from this age window, we specifically mentioned these deviations in the respective methods section. However, various studies found a rather gradual change in behavioral observations of mice of increasing age (Liao et al., 2024; Shoji et al.,2019), with more pronounced distinctions often visible in mice older than eight months (Shoji et al., 2016). Using a two-way ANOVA to account for age and genotype as separate factors, we found no significant impact of age on the coat state of mice tested in this study. Accordingly, the distribution of determined coat state ranks relative to the respective animals' age did not indicate a correlation between these factors:

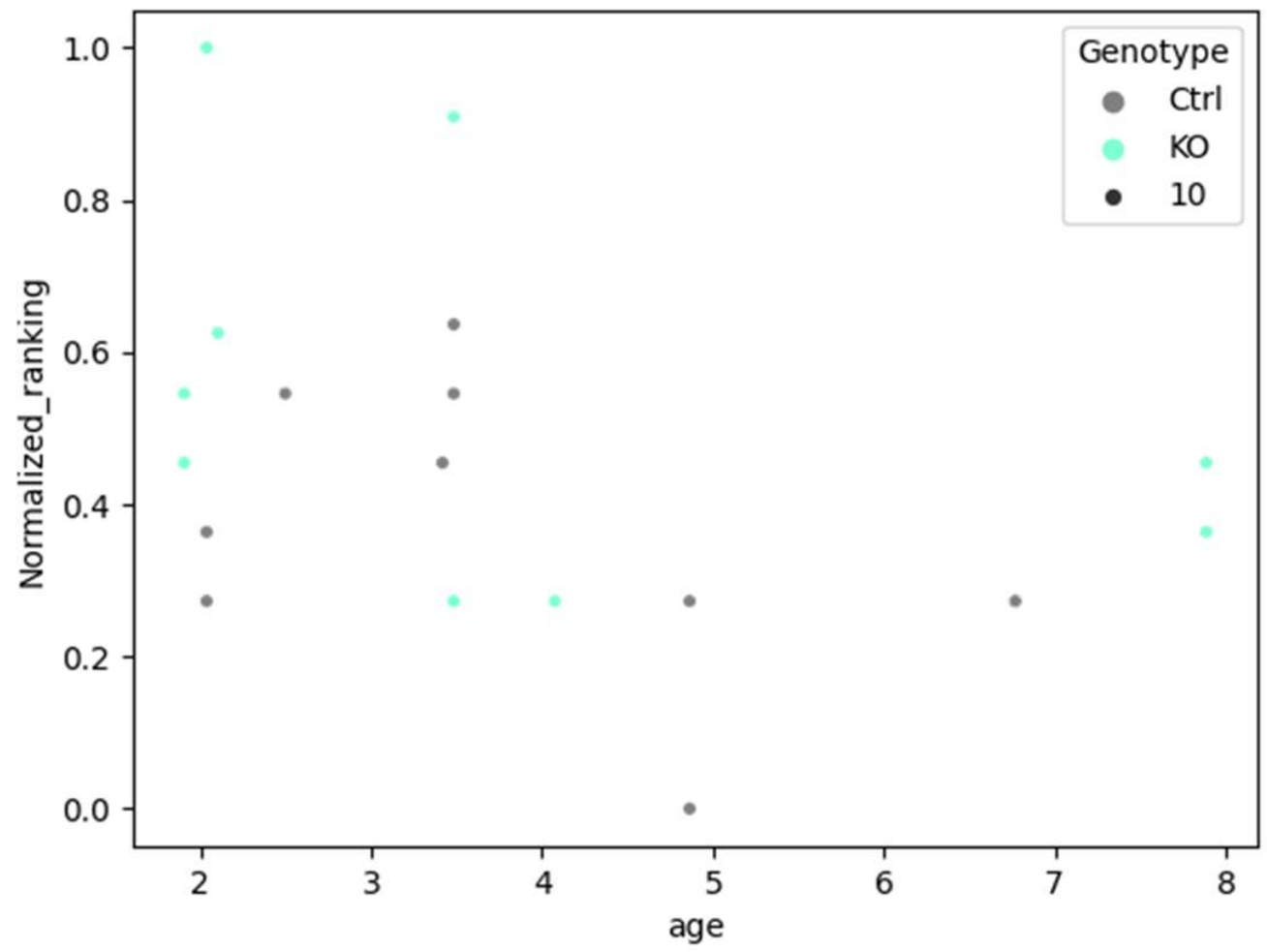

We therefore presumed that the observed behavior of mice used in the coat state assessment in this study is not closely linked to age in the investigated age range and our data thus largely reflect an adult phenotype of the used mouse strains.

Liao GY, Pettan-Brewer C, Ladiges W. Comparison of Age-Related Decline and Behavioral Validity in C57BL/6 and CB6F1 Mice. bioRxiv [Preprint]. 2024 Jun 14:2024.06.14.599036. doi: 10.1101/2024.06.14.599036. PMID: 38915625; PMCID: PMC11195229.

Shoji H, Takao K, Hattori S, Miyakawa T. Age-related changes in behavior in C57BL/6J mice from young adulthood to middle age. Mol Brain. 2016 Jan 28;9:11. doi: 10.1186/s13041-016-0191-9. PMID: 26822304; PMCID: PMC4730600.

Shoji H, Miyakawa T. Age-related behavioral changes from young to old age in male mice of a C57BL/6J strain maintained under a genetic stability program. Neuropsychopharmacol Rep. 2019 Jun;39(2):100-118. doi: 10.1002/npr2.12052. Epub 2019 Feb 27. PMID: 30816023; PMCID: PMC7292274.

11. Extended Data Figure 3, panel d. The legend reads "Blood levels of various appetite-regulating hormones were unaltered by the Dnmt1-knockout (n(Control) = 19, n(Knockout) = 16)" but the data points per hormone seem lower than indicated. Please clarify.

    Upon editors' request, we had handed in a corrected version of the extended data, where numbers of N for each subset of data were provided. In this corrected version we had written that for panel d the number of independent individuals N=6, which fits to the data points.

12. Figure 3a. In the lower panel, please add the SEM to the mean running time averaged per genotype in the lower panel so that the reader can assess intra-genotype variance. Was there statistical testing performed here?

    We thank the reviewer for pointing this out. We added the SEMs in the mentioned figure and conducted statistical testing for further quantification of the qualitative description in daytime-dependent running wheel usage. We used a linear mixed-effects model to quantify differences based on genotype and daytime. As we found an overall significant effect of the genotype as well as a significant interaction between genotype and daytime, we used t-tests for post-hoc testing of the significant interaction. This highlighted the knockout-dependent decrease in activity throughout the dark periods, i.e., the animals' active phase of the day. The figure and figure legends were correspondingly updated.

13. For the single-nuclues RNAseq data, the Methods read: "For each genotype, tissue from the three mice was pooled prior to nuclei extraction to obtain sufficient material and minimize inter-individual variability". This is problematic if after pooling the biological samples are n=1/genotype, because it is hard to assess that the differences seen are driven by genotype rather than variability between two samples in the workflow. This might be driving the rather profound differences in the representations of certain populations, as shown in Extended Data Figure 7, especially considering that this is a conditional mouse model.

We thank the reviewer for this important methodological point. We acknowledge that pooling tissue from three mice per genotype before nuclei extraction yields a single sample per genotype, and that this design inherently limits the ability to formally distinguish genotype-driven effects from inter-sample technical variability. We would like to clarify the rationale and context for this approach.

The split-pool combinatorial barcoding method we employed (Parse Biosciences SPLiT-seq chemistry) requires a minimum of approximately 100,000 nuclei per experiment to achieve adequate depth, as material can get lost during the fixation as well as the washing steps during the library preparation. Pooling tissue from three animals per genotype was therefore technically necessary to meet this requirement while preserving the in-solution combinatorial barcoding workflow, in which nuclei from all conditions are processed simultaneously in the same reaction, thereby minimizing batch effects and technical variability between conditions. This is a well-recognized advantage of split-pool designs (Rosenberg et al., 2018). Importantly, the three mice per genotype were chosen to represent the biological diversity of the cohort, and pooling prior to nuclei extraction means that each genotype pool contributes nuclei from multiple animals, reducing the influence of any single individual on the transcriptional profile.

We further note that this approach is widely used for discovery-level cortical snRNA-seq studies, including foundational atlas-building experiments in which a single sample or a small number of pooled samples per condition are used for transcriptome-wide profiling (Tasic et al., 2016; 2018). The central conclusions of our snRNA-seq data on PNN disruption are independently validated by histological WFA staining across multiple biological replicates (N = 4–6 mice per genotype), providing the confirmatory experimental evidence.

Rosenberg, A.B. et al. (2018). Single-cell profiling of the developing mouse brain and spinal cord with split-pool barcoding. Science, 360(6385), 176–182.

Tasic, B., Menon, V., Nguyen, T. N., Kim, T. K., Jarsky, T., Yao, Z., ... Zeng, H. (2016). Adult mouse cortical cell taxonomy revealed by single cell transcriptomics. Nature Neuroscience, 19(2), 335–346

Tasic, B., Yao, Z., Graybuck, L. T., Smith, K. A., Nguyen, T. N., Bertagnolli, D., ... Zeng, H. (2018). Shared and distinct transcriptomic cell types across neocortical areas. Nature, 563(7729), 72–78.

14. Do the authors see reduced expression of Dnmt1 as expected in PV interneurons? Also, were the authors able to previously reported Dnmt1 targets?

We thank the reviewer for this question. We examined *Dnmt1* expression in PV+ interneurons at both the mean normalized expression level (log2FC = −0.206) and the proportion of *Dnmt1*-expressing nuclei (Ctrl: 63.5%, KO: 59.4%, log2FC = −0.097, Fisher's exact test p = 0.29). While the direction of change is consistent with *Dnmt1* deletion, neither comparison reached statistical significance.

This outcome is expected for several well-established reasons. Cre-lox recombination deletes two exons of *Dnmt1* at the DNA level, and snRNA-seq captures primarily unspliced nuclear pre-mRNA alongside mature transcripts (La Manno et al., 2018). Residual pre-mRNA transcription despite the deleted exons can therefore persist even after successful recombination, producing detectable *Dnmt1* reads in recombined nuclei (Yang et al., 2008). Further, our pooled n=1 snRNA-seq design lacks the statistical power to detect modest expression differences in a small interneuron cluster, a limitation inherent to discovery-level experimental design. The reduced sensitivity of snRNA-seq relative to whole-cell approaches for detecting expression changes in specific subpopulations has been previously noted (Thrupp et al., 2020; Wu et al., 2019).

The functional effectiveness of the knockout is instead confirmed by the significantly elevated spontaneous PV firing rates, reduced inhibitory efficacy upon optogenetic activation, and diminished WFA-labeled PNN intensity, all of which are direct functional and structural readouts of DNMT1 loss in PV interneurons replicated across multiple animals (*N* = 6–13 per genotype). Moreover, in our previous publication characterizing this mouse line (Hahn et al., 2020; Ref 18 in the manuscript), DNMT1 loss in PV interneurons was validated at the protein level.

Yang, S.H. et al. (2008). Caution! Analyze transcripts from conditional knockout alleles. Nucleic Acids Research, 36(22), e93.

Thrupp, N. et al. (2020). Single-nucleus RNA-seq is not suitable for detection of microglial activation genes in humans. Cell Reports, 32(13), 108189.

Wu, H. et al. (2019). Advantages of single-nucleus over single-cell RNA sequencing of adult kidney: rare cell types and novel cell states revealed in fibrosis. Journal of the American Society of Nephrology, 30(1), 23–32.

La Manno, G. et al. (2018). RNA velocity of single cells. Nature, 560, 494–498.

15. The methods also indicate that "the top 50 upregulated and top 50 downregulated marker genes per cluster were cross-referenced with established cell type-specific markers from published literature and publicly available reference databases" without providing references for the publications that were used for annotations of clusters.

We thank the reviewer for pointing this out; we have added the references to our manuscript.

All in all, the conclusion that "single-nucleus transcriptomics reveals non–cell-autonomous glial remodeling" is not entirely supported by the current data.

We further adjusted the title of our manuscript and re-phrased our statements in the results and the discussion.

16. The volcano plot in Extended Data Figure 8 is puzzling (distribution of the non-significant genes). Please explain.

The appearance of the volcano plot in Extended Data Figure 8 reflects a well-documented consequence of differential expression testing in very small cell clusters in single-cell data. The perineuronal oligodendrocyte sub-population identified in our analysis is defined by *Pdgfc* positivity and represents a small subset of the broader oligodendrocyte lineage. When a Wilcoxon rank-sum test (as implemented in Seurat) is applied to a very limited number of cells, two opposing artifacts arise simultaneously: (1) genes with even modest expression differences achieve extreme statistical power due to the small denominators in the rank-test, resulting in very low raw p-values that, after Benjamini-Hochberg correction, are rounded to display as 0 in standard floating-point precision; and (2), the vast majority of genes with no meaningful difference show similarly inflated test statistics, compressing them into a narrow, flat band at the base of the volcano plot. This is not an analysis error but rather an expected statistical artifact of small-N subclustering in snRNA-seq data, and has been noted previously in the single-cell literature (Andrews & Hemberg, 2019).

Andrews, T.S. & Hemberg, M. (2019). False signals induced by single-cell imputation. F1000Research, 7, 1740.

17. Lines 196-199. Please briefly introduce neuronal populations and subcortical regions regulating feeding investigated in Extended Data Figure 4-5 for readers who are not familiar with these circuits.

Body weight regulation is controlled by a complex interplay between circulating metabolic hormones, central neuronal circuits, and peripheral mechanisms that together regulate food intake, energy storage, and expenditure (46,47). Appetite-regulating hormones can exert either orexigenic (appetite-promoting) or anorexigenic (appetite-suppressing) effects and are primarily sensed by neurons in the hypothalamus, particularly within the arcuate nucleus, a key integrative center for energy homeostasis (46,48). The arcuate nucleus is extensively interconnected with other brain regions and coordinates feeding behavior and metabolic processes relevant for body weight control (49,50).

Within the arcuate nucleus, two major and functionally opposing neuronal populations have been extensively characterized: agouti-related peptide (AgRP)-expressing neurons, which promote feeding and mediate orexigenic signaling, and proopiomelanocortin (POMC)-expressing neurons, which suppress appetite and mediate anorexigenic pathways (50). In addition to regulating food intake, subsets of POMC neurons have also been implicated in the control of physical activity and energy expenditure (55,56).

In line with this framework, we assessed circulating levels of key appetite-regulating hormones in Pvalb-Cre/Dnmt1-KO and control mice. With the exception of leptin, no significant differences were observed. Specifically, levels of the orexigenic hormone ghrelin—which is released from the gut and promotes feeding via hypothalamic receptors (51)—were unchanged. Similarly, the glucose-regulating hormones insulin and glucagon were not altered. We also did not detect differences in glucagon-like peptide-1 (GLP-1), which is released postprandially and promotes insulin secretion while inhibiting glucagon release, nor in peptide YY (PYY), which exerts anorexigenic effects (52).

Notably, leptin levels were increased in Pvalb-Cre/Dnmt1-KO mice. However, as leptin is primarily produced by adipose tissue and correlates strongly with body fat mass (53,54), this increase most likely reflects the elevated body weight rather than a primary alteration in endocrine regulation. This interpretation is further supported by the fact that leptin activates anorexigenic pathways (46), making it unlikely to drive increased body weight.

Consistent with the absence of major hormonal alterations affecting feeding, we observed very low numbers of Pvalb-positive cells in the arcuate nucleus in both genotypes (Extended Data Fig. 5). To further assess whether PV-expressing cells overlap with key feeding-related neuronal populations, we performed immunostaining for markers of POMC and AgRP neurons. Using β-endorphin as a marker for POMC-expressing neurons (57,58), we found no co-localization with tdTomato-positive cells, indicating that PV is not expressed in POMC neurons. In contrast, partial overlap with GABAergic neurons—consistent with the known phenotype of AgRP neurons (59)—was observed, although this overlap was limited.

Taken together, these findings indicate that *Dnmt1* deletion in PV-expressing cells is unlikely to directly interfere with the core hypothalamic feeding circuits. The low abundance of PV-positive neurons in the arcuate nucleus, combined with the lack of substantial hormonal changes affecting appetite, suggests that the observed increase in body weight is not driven by altered feeding regulation but more likely reflects changes in energy expenditure or physical activity.

Barsh, G. S. & Schwartz, M. W. Genetic approaches to studying energy balance: Perception and integration. Nature Reviews Genetics vol. 3 Preprint at https://doi.org/10.1038/nrg862 (2002).

Yu, J. H. & Kim, M. S. Molecular mechanisms of appetite regulation. Diabetes and Metabolism Journal vol. 36 Preprint at https://doi.org/10.4093/dmj.2012.36.6.391 (2012).

Fry, M., Hoyda, T. D. & Ferguson, A. V. Making sense of it: Roles of the sensory circumventricular organs in feeding and regulation of energy homeostasis. Experimental Biology and Medicine vol. 232 Preprint at (2007).

Elmquist, J. K., Elias, C. F. & Saper, C. B. From lesions to leptin: Hypothalamic control of food intake and body weight. Neuron vol. 22 Preprint at https://doi.org/10.1016/S0896-6273(00)81084-3 (1999).

Timper, K. & Brüning, J. C. Hypothalamic circuits regulating appetite and energy homeostasis: Pathways to obesity. DMM Disease Models and Mechanisms 10, (2017).

Nakazato, M. et al. A role for ghrelin in the central regulation of feeding. Nature 409, (2001).

Ahima, R. S. & Antwi, D. A. Brain Regulation of Appetite and Satiety. Endocrinology and Metabolism Clinics of North America vol. 37 Preprint at https://doi.org/10.1016/j.ecl.2008.08.005 (2008).

Ostlund, R. E., Yang, J. W., Klein, S. & Gingerich, R. Relation between plasma leptin concentration and body fat, gender, diet, age, and metabolic covariates. J Clin Endocrinol Metab 81, (1996).

Halaas, J. L. et al. Physiological response to long-term peripheral and central leptin infusion in lean and obese mice. Proc Natl Acad Sci U S A 94, (1997).

Zhang, Y. et al. Simultaneous POMC gene transfer to hypothalamus and brainstem increases physical activity, lipolysis and reduces adult-onset obesity. European Journal of Neuroscience 33, (2011).

Burke, L. K. et al. Sex difference in physical activity, energy expenditure and obesity driven by a subpopulation of hypothalamic POMC neurons. Mol Metab 5, (2016).

Millington, G. W. M. The role of proopiomelanocortin (POMC) neurones in feeding behaviour. Nutrition and Metabolism vol. 4 Preprint at https://doi.org/10.1186/1743-7075-4-18 (2007).

Sotonyi, P., Gao, Q., Bechmann, I. & Horvath, T. L. Estrogen promotes parvalbumin expression in arcuate nucleus POMC neurons. Reproductive Sciences 17, (2010).

18. Figure 6. Please show individual data points for plots in panels e-f. The legend indicates that these are "N = 3 biological replicates with n = 3 slices each per group" and "N = 3 biological replicates with n = 8 slices per genotype". Have values across slices averaged or have the authors conducted a nested analysis?
Thank you for raising this point. We have now included individual data points in panels e-f as requested. In our analysis, each biological replicate consists of matched pairs of Ctrl and KO animals at comparable anatomical positions. For each brain, multiple slices (n = 2-3 per brain) were analyzed at corresponding positions. Rather than averaging the values per genotype across slices, which would reduce the effective sample size and obscure the underlying variability within each animal, we chose to retain the paired structure of the data. These values were then used for statistical comparison between Ctrl and KO groups using a paired t-test, taking into account the matched experimental design.

Reviewer #3

There is growing evidence that perturbation of epigenetic processes can be associated with a variety of neurodevelopmental disorders. The authors previously demonstrated that loss of the DNA methyltransferase 1 (DNMT1) in PV+ interneurons increases their mIPSC frequency, and bulk sequencing of PV+ cells revealed that this was in part due to alterations in endocytosis and vesicle replacement (PMID: 32147726). Here the authors expand on these observations to with additional electrophysiology, behavior and single cell sequencing experiments to reveal an array of deficits when DNMT1 is removed from PV+ interneurons. Overall this manuscript provides interesting insights regarding DNMT1 function in PV+ cells and cortical circuits. But I am somewhat underwhelmed by the conclusions from their single cell sequencing experiments, which

is due in part to insufficient methodological details about cell numbers & definitions of cell subtypes, and lack of confirmation of predicted gene expression changes. My specific comments are below.

Major points

1. The authors need to provide additional details about their single cell sequencing methodology & results. It is standard to report number of cells sequenced for each bio rep (WT and mutant), average reads/cell, genes/cell, etc. either in the methods or as a supplementary table.

   We have provided the relevant information in the methods section.

2. Similarly, for all bioinformatic analysis between 2 tissues/cell types (such as the volcano plots in Fig 4C), the authors should provide a Supplementary Table or source file with the log fold change & adjusted p-value for every gene.

   We have compiled the source tables with log2FC and adjusted *p* values and provided it as Extended Data File 2.

3. I'm concerned about the reliability of some of the author's conclusions about proportional changes from the single cell sequencing data because of lack of power (n = 1 biological rep) & unknown cell numbers in some clusters (as noted above). I am not a bioinformatician, but I believe that adjusted p-values (rather than p-values) should be reported for the 2-sample proportion test, and it's not clear from me in the legends or methods which value was reported.

   We thank the reviewer for their comment. These values are indeed adjusted *p* values. The figure legend has been adjusted accordingly and an overview table has been provided as Extended Data File 1.

4. And it seems like a potential over-interpretation of the data for clusters with very small cell numbers (endothelial cells & corticospinal neurons, for example). The authors should consider applying tools like DA-seq (PMID: 34001664) that account for the nuances of snRNA-seq data.

   We thank the reviewer for the suggestion to apply DA-seq to our dataset. However, our experimental design with a single biological replicate per condition does not satisfy the statistical requirements of DA-seq. Specifically, DA-seq relies on a permutation-based null distribution that requires sufficient biological replicates to generate a valid significance threshold; with n = 1 per group, no meaningful permutation p-value can be computed [Zhao et al., 2021]. We therefore applied Milo [Dann et al., 2022], a neighbourhood-based differential abundance method built on a negative binomial generalized linear model, which is well-suited for datasets with limited replication and additionally allows covariates to be modeled in the design formula. We believe this represents a statistically more rigorous approach for our experimental setup than DA-seq.

   J. Zhao, A. Jaffe, H. Li, O. Lindenbaum, E. Sefik, R. Jackson, X. Cheng, R.A. Flavell, & Y. Kluger, Detection of differentially abundant cell subpopulations in scRNA-seq data, Proc. Natl. Acad. Sci. U.S.A. 118 (22) e2100293118, https://doi.org/10.1073/pnas.2100293118 (2021).

   Dann, E., Henderson, N.C., Teichmann, S.A. et al. Differential abundance testing on single-cell data using k-nearest neighbor graphs. Nat Biotechnol 40, 245–253 (2022). https://doi.org/10.1038/s41587-021-01033-z

5. Or alternatively, the authors should perform immunostaining with cell counts to confirm that some of these reported proportional differences of cell types between WT and KO mice are confirmed in biological samples.

   We thank the reviewer for this suggestion. Following the reviewer's recommendation to apply an independent analytical method (point 4), we re-analyzed cell-type proportions using Milo, a neighbourhood-based differential abundance framework built on a negative binomial GLM [Dann et al., 2022]. Milo confirmed the abundance shifts originally identified by two-sample proportion tests, including the enrichment of corticospinal neuron populations and changes in excitatory layer-specific subclasses.

   Regarding immunostaining-based cell counts, we recognize the general value of such validation; however, the cell-type shifts we report are defined at the transcriptional subtype level, resolving populations that lack distinct, commercially available antibodies for the specific markers used to demarcate them. Protein-level discrimination of these transcriptomically defined subpopulations in tissue sections is therefore not currently feasible.

6. If the authors truly believe that there is a 3-4 fold change in some cell types with very low cell numbers, as opposed to the expected biological 'noise' in these types of experiments, then that should be easy to demonstrate via immunos (or FISH).

We thank the reviewer for raising this important point. We agree that, in principle, strong abundance shifts warrant independent validation. As described in our response to point 4, independent differential abundance analysis with Milo corroborates the directional changes reported from proportion tests, lending methodological support to the findings. Regarding in situ hybridization (e.g. RNAscope) or immunostaining for these specific transcriptomically defined subpopulations, we note that establishing multiplexed FISH panels for rare, transcriptomically-defined neuron subtypes lacking specific protein markers lies beyond the feasible scope of the present study. The affected clusters, in particular the corticospinal neuron subsets, are defined by combinations of multiple marker genes rather than a single, targetable transcript, making panel design technically challenging. We have added a clear statement to the manuscript noting that the characterization of these shifts, while consistently detected by both proportion testing and Milo, remains a subject for future, dedicated investigation, which is beyond the scope of this study.

7. A significant weakness is that the authors do not perform any validation of predicted DEGs from their single cell sequencing dataset. This is especially problematic considering my concerns above about the power of these analyses due to low cell numbers of some clusters. The authors identified several ligand-receptor pairs between PV+ cells and oligos that were significantly up- or down-regulated in KO mice (Figure 5D-E). At a minimum, the authors should perform in situ hybridizations or immunohistochemistry to confirm differential expression of some of these ligand-receptor cells in PV+ cells and oligos. Showing loss of PNNs with WFA staining is nice & consistent with the e-phys deficits, but it does not directly address any of the putative DEGs from their scRNAseq dataset.

We appreciate the reviewer's concern regarding DEG validation. We wish to clarify the scope and purpose of the cell–cell communication analysis in our study: the CellChat results presented in Figure 5D–E are not intended to identify specific DEGs for mechanistic follow-up, but rather to characterize shifts in predicted communication probabilities between PV interneurons and oligodendrocyte lineage cells at the pathway level. Our primary claim is that PV–oligodendrocyte signaling is broadly reorganized in the KO cortex, not that any individual ligand–receptor pair is definitively up- or down-regulated as a validated molecular finding.

The histological validation using WFA staining (Fig. 6) directly confirms the biological endpoint of this signaling reorganization, namely, impaired perineuronal net integrity. This provides an orthogonal, protein-level evidence consistent with the transcriptional changes inferred from snRNA-seq. We therefore consider the WFA data to be an appropriate functional validation of the overall conclusion. We have revised the relevant text to make this interpretive framing more explicit, and to avoid any impression that individual ligand–receptor pairs are claimed as validated molecular findings requiring independent confirmation.

8. The authors inconsistent classification of oligodendrocytes is confusing and it's not clear to me what their basis is for defining these subpopulations. In Fig 4, there are 3 classes defined as OPCs, newly mature oligos and myelinating oligos. I do not know what the term 'newly mature' means, is that newborn immature/non-myelinating oligos? I'd recommend renaming.

Please see response to point 10

9. Based on the Fig 4A UMAP, it appears that these 'new oligos' and 'myelinating oligos' are salt-&-pepper in the same cluster. What is the basis for differentiating these 2 types of oligos?

Please see response to point 10

10. The gene expression and heatmaps in Fig 4B & 4E, respectively, are basically indistinguishable, so I don't understand this classification. I strongly recommend just combining these 2 subgroups as 'oligodendrocytes' unless they can justify this distinction.

Respons to points 8–10: We thank the reviewer for this point. We can see why the non-standard terminology "newly mature oligodendrocytes" can be potentially confusing, particularly given that the UMAP shows these cells in overlapping regions with "myelinating oligodendrocytes." Our subclassification was originally based on the foundational single-cell transcriptomic characterization of the oligodendrocyte lineage by Marques et al. (2016), which identified distinct transcriptional states along the maturation trajectory. Our "newly mature oligodendrocytes" were intended to correspond to their "newly formed oligodendrocytes" (NFOLs).!However, we recognize that this mapping was not clearly communicated, and that the overlapping UMAP positions make the distinction difficult to justify visually.

In response to this comment, we have taken the following steps:

1) We have renamed "newly mature oligodendrocytes" to "newly formed oligodendrocytes (NFOLs)" throughout the manuscript, aligning our nomenclature with Marques et al. (2016) and the standard in the field (Valihrach et al., 2022).

2) We have added the key marker gene distinguishing NFOLs from MOLs (*Enpp6*).
3) For the "perineuronal oligodendrocytes" (see response to Comment 11 below), we clarify that this is a sub-population of NFOLs defined additionally by *Pdgfc* expression, as established by Szuchet et al. and Tian et al. (cited in the manuscript as references 42, 43).

Marques, S., Zeisel, A., Codeluppi, S., van Bruggen, D., Mendanha Falcão, A., Xiao, L., Li, H., Häring, M., Hochgerner, H., Romanov, R. A., Grun, D., Altelaar, M. A. F., Muñoz Manchado, A. B., Bakken, T. E., Lein, E., Hjerling-Leffler, J., Harkany, T., Richardson, W. D., Linnarsson, S., & Castelo-Branco, G. (2016). Oligodendrocyte heterogeneity in the mouse juvenile and adult central nervous system. Science, 352(6291), 1326–1329.

Valihrach, L. et al. (2022). Recent advances in deciphering oligodendrocyte heterogeneity with single-cell transcriptomics. Frontiers in Cellular Neuroscience, 16, 1025012.

11. Then later in the results the authors add a fourth group 'perineuronal oligos' and include this in Fig 5 analyses. Again I have no idea what genes distinguishes this subset from other subset, which cluster/subtype they arise from, why it was only analyzed in Fig 5 and not Fig 4, etc.

We thank the reviewer and apologize for this confusing presentation. "Perineuronal oligodendrocytes" are not an additional fourth cluster independently identified from the UMAP; they are a transcriptionally defined sub-population within the NFOLs, identified by their enrichment for *Pdgfc* expression. *Pdgfc*-positive oligodendrocytes have been specifically described in the literature as a population that localizes around neuronal cell bodies (hence "perineuronal"), synthesizes ECM components including brevican and tenascin-R, and contributes to PNN maintenance (Szuchet et al.; Tian et al.; Refs. 42, 43 in the manuscript). They were not analyzed separately in Figure 4 because their defining characteristic is not a unique cluster identity but rather a gene expression gradient within the broader cluster. We analyzed them selectively in the context of Figure 5, where our focus was specifically on the PV–oligodendrocyte axis and PNN-relevant signaling, because their *Pdgfc*+ identity marks them as a directly relevant subset for ECM support of PV cells.

12. And to reiterate my earlier concerns, the more you subcluster the data, the fewer cells & less power the analyses have, which is why you get a volcano plot like Ext Data 8 where there are so few DEGs and all genes have an adj p-value of 0.

As well pointed out by the reviewer, the appearance of the volcano plot in Extended Data Figure 8 reflects a well-documented consequence of differential expression testing in very small cell clusters in single-cell data. The perineuronal oligodendrocyte sub-population identified in our analysis is defined by *Pdgfc* positivity and represents a small subset of the broader oligodendrocyte lineage. When a Wilcoxon rank-sum test (as implemented in Seurat) is applied to a very limited number of cells, two opposing artifacts arise simultaneously: (1) genes with even modest expression differences achieve extreme statistical power due to the small denominators in the rank-test, resulting in very low raw p-values that, after Benjamini-Hochberg correction, are rounded to display as 0 in standard floating-point precision; and (2), the vast majority of genes with no meaningful difference show similarly inflated test statistics, compressing them into a narrow, flat band at the base of the volcano plot. This is not an analysis error but rather an expected statistical artifact of small-N subclustering in snRNA-seq data, and has been noted previously in the single-cell literature (Andrews & Hemberg, 2019).

Andrews, T.S. & Hemberg, M. (2019). False signals induced by single-cell imputation. F1000Research, 7, 1740.

13. In my opinion, the authors need to redo this oligo analyses by clearly defining these 3-4 subtypes of oligos in the beginning (based on gene expression and/or mapping them onto reference databases) and use these subtypes in their analysis in Fig 4 & 5. Or alternatively, the authors should just use 'OPCs' and 'mature oligos' for their downstream analyses. And the authors should generate Oligo-only UMAPs with these subtypes clearly labeled (and ideally with a companion WT/KO labeled UMAP). As it stands, I really don't know what to make of this entire oligo analyses for all these reasons stated above.

The cluster designated myelinating oligodendrocytes was identified by the robust and coordinated upregulation of established structural myelin genes, consistent with a terminally differentiated, actively myelinating state. Specifically, the top upregulated markers included the core compact myelin proteins *Mbp* (avg_log2FC = 3.76), *Plp1* (3.97), *Mog* (3.94), *Mag* (3.80), *Mobp* (3.98), alongside myelin-associated lipid synthesis enzymes *Fa2h* (3.78) and *Ugt8a* (3.71), the myelin stability factor *Aspa* (3.76), and the myelination regulator *Myrf* (3.92). This expression profile is in line with the MOL signatures described by Marques et al. (2016) and the broader mouse nervous system cell atlas of Zeisel et al. (2018).

The newly mature oligodendrocyte cluster was instead most consistent with the newly formed oligodendrocyte (NFOL) stage, defined by the initiation of the myelination program together with the expression of early

differentiation markers. Canonical myelin genes were upregulated relative to all other clusters, including *Mag* (avg_log2FC = 5.56), *Mog* (5.47), *Plp1* (5.36), *Mobp* (5.31), and *Opalin* (5.27). Importantly, this cluster also showed strong upregulation of *Enpp6*, one of its strongest markers in the one-vs-all analysis (5.91) and a well-established marker of newly formed or early differentiating oligodendrocytes that declines with progression into more mature myelinating states, thereby further supporting annotation of this population as newly mature/NFOL rather than fully myelinating oligodendrocytes. This interpretation is further supported by the fact that canonical OPC markers such as *Pdgfra*, *Cspg4*, and *Gpr17*, are not retained, indicating that the newly mature oligodendrocyte population has progressed beyond the precursor state.

Teng, X.-Y., Hu, P., Zhang, C.-M., Zhang, Q.-X., Yang, G., Zang, Y.-Y., Liu, Z.-X., Chen, G., & Shi, Y. S. (2024). OPALIN is an LGI1 receptor promoting oligodendrocyte differentiation. Proceedings of the National Academy of Sciences of the United States of America, 121(32), e2403652121.

Jiang, C., Qiu, W., Yang, Y., Huang, H., Dai, Z.-M., Yang, A., Tang, T., Zhao, X., & Qiu, M. (2023). ADAMTS4 Enhances Oligodendrocyte Differentiation and Remyelination by Cleaving NG2 Proteoglycan and Attenuating PDGFRα Signaling. The Journal of Neuroscience : The Official Journal of the Society for Neuroscience, 43(24), 4405–4417.

Marques, S., Zeisel, A., Codeluppi, S., van Bruggen, D., Mendanha Falcão, A., Xiao, L., Li, H., Häring, M., Hochgerner, H., Romanov, R. A., Grun, D., Altelaar, M. A. F., Muñoz Manchado, A. B., Bakken, T. E., Lein, E., Hjerling-Leffler, J., Harkany, T., Richardson, W. D., Linnarsson, S., & Castelo-Branco, G. (2016). Oligodendrocyte heterogeneity in the mouse juvenile and adult central nervous system. Science, 352(6291), 1326–1329.

Satija, R., Farrell, J. A., Gennert, D., Schier, A. F., & Regev, A. (2015). Spatial reconstruction of single-cell gene expression data. Nature Biotechnology, 33(5), 495–502.

Tasic, B., Yao, Z., Graybuck, L. T., Smith, K. A., Nguyen, T. N., Bertagnolli, D., Goldy, J., Garren, E., Economo, M. N., Viswanathan, S., Penn, O., Bakken, T., Menon, V., Miller, J., Fong, O., Hirokawa, K. E., Lathia, K., Rimorin, C., Tieu, M., … Zeng, H. (2018). Shared and distinct transcriptomic cell types across neocortical areas. Nature, 563(7729), 72–78.

Zeisel, A., Hochgerner, H., Lönnerberg, P., Johnsson, A., Memic, F., van der Zwan, J., Häring, M., Braun, E., Borm, L. E., La Manno, G., Codeluppi, S., Furlan, A., Lee, K., Skene, N., Harris, K. D., Hjerling-Leffler, J., Arenas, E., Ernfors, P., Marklund, U., & Linnarsson, S. (2018). Molecular architecture of the mouse nervous system. Cell, 174(4), 999–1014.e22.

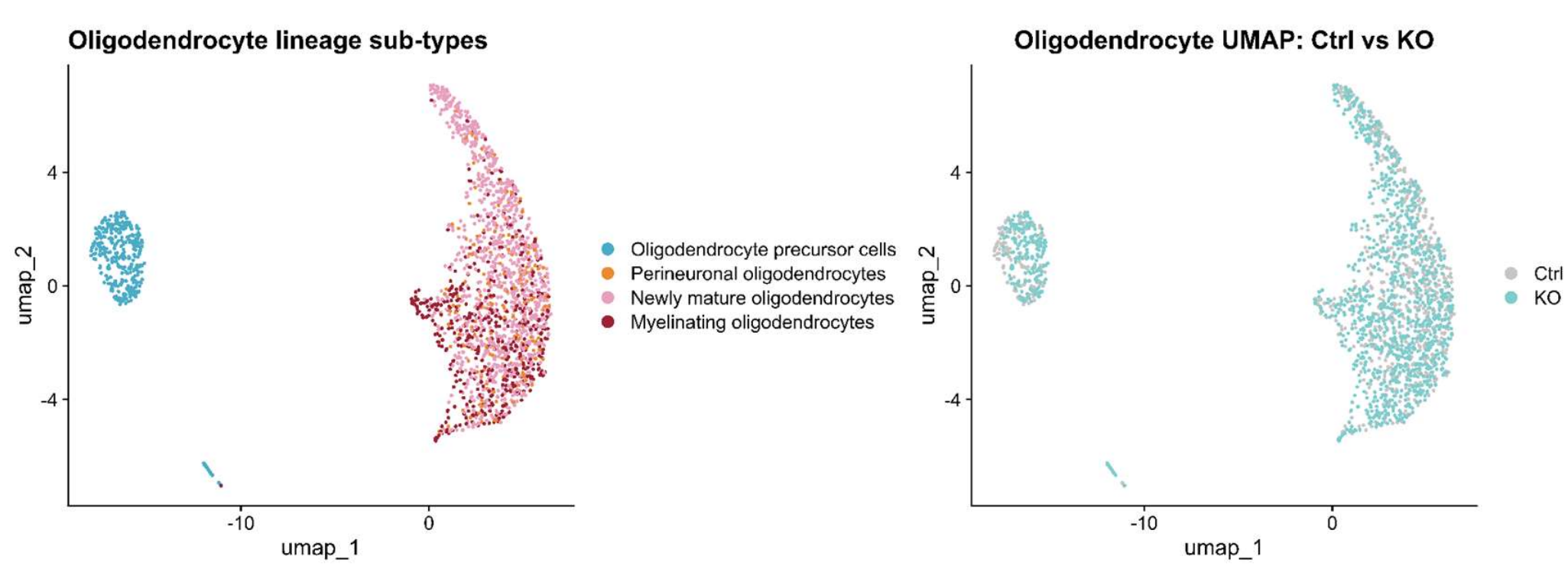

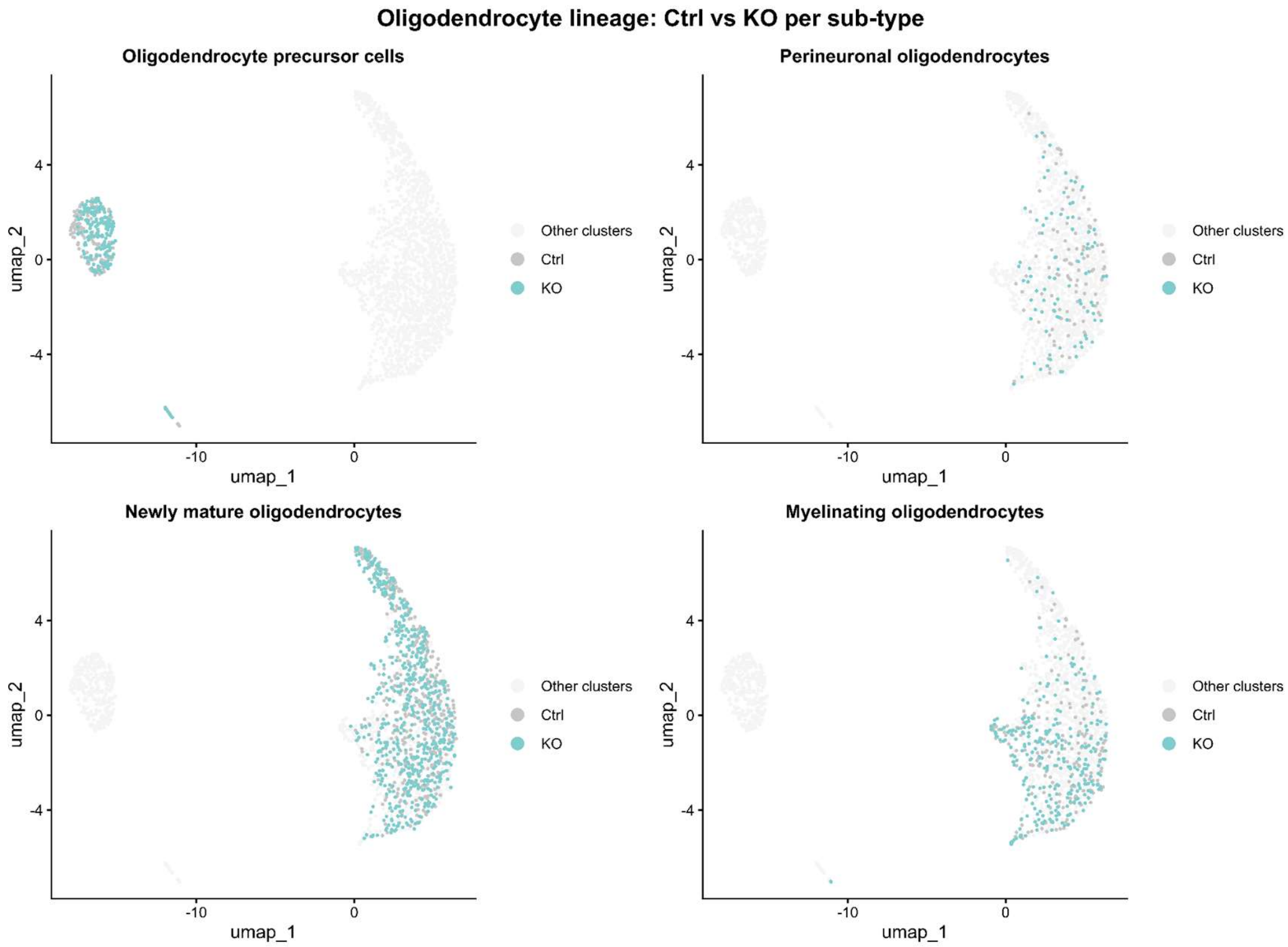

14. The authors show that there is no difference in PV cell numbers in the hypothalamus (Ex Data 4), which agrees with their previous report of no differences in PV+ cortical interneurons in DNMT1 mutant mice (PMID: 32793592). They then claim that PV cell identity is "unaltered in [knockout] mice" (title of Ext Data Fig 5) simply because beta-endorphin, a marker of POMC cells, does not overlap with PV immunos. Based on this observation, the authors claim that it is "…unlikely that Dnmt1 deletion in PV cells directly interferes with hypothalamic feeding circuits" (lines 199-200). I do not agree with this conclusion for several reasons. First, just because a single marker does not overlap with PV cells in WT and mutant mice does not justify the conclusion that PV+ cells in the hypothalamus are unaltered. Second, the authors clearly demonstrate (and spend a significant amount of time discussing) the non-cell autonomous changes in genes expression of cortical cells when Dnmt1 is removed only in cortical PV+ interneurons. Why do the authors not think the same type of non-cell autonomous effects occur within the hypothalamic feeding circuit? My assumption is that performing single cell sequencing on hypothalamic cells would likely reveal non-cell autonomous DEGs in these cells from loss of Dnmt1 in PV+ cells, just like in the cortex.

We thank the reviewer for this important critique and fully agree that the non-overlap of PV cells with POMC-expressing neurons alone does not demonstrate that hypothalamic PV cells are functionally unaltered. We have revised the title of Extended Data Fig. 5 and the corresponding interpretive statement to reflect this more precisely: our data show that the *Dnmt1*-targeted PV population does not overlap with the principal POMC/AgRP neuronal populations that drive feeding, but we do not claim broader functional integrity of hypothalamic PV cells. We have to state clearly, that performing single cell sequencing on hypothalamic cells is way beyond the focus of this study and our current resources. It simply cannot be done.

We also fully acknowledge the reviewer's conceptually valid point that non-cell-autonomous transcriptional could in principle occur in hypothalamic circuits. However, we would argue that our existing functional and metabolic data argue against a primary hypothalamic feeding circuit disturbance as the driver of the observed phenotype. Specifically, food intake was unchanged, and a comprehensive hormonal panel, including ghrelin, insulin, GLP-1, PYY, and glucagon, showed no significant alterations in KO mice (Extended Data Fig. 3f). The only exception was elevated leptin, which is most likely a secondary consequence of increased fat mass rather than primary endocrine dysregulation. This hormonal profile is inconsistent with a primary disturbance of hypothalamic appetite or satiety circuits. Instead, the observed increase in body weight together with reduced voluntary running and home-cage activity points to diminished energy expenditure which is a cortical/motivational phenotype rather than altered hypothalamic feeding regulation.

We do not exclude the possibility that secondary, non-cell-autonomous transcriptional changes may exist in hypothalamic cells of KO mice, as the reviewer appropriately suggests. However, characterizing the full extent of such changes across all brain regions expressing PV interneurons lies beyond the scope of the present study, which is focused on cortical circuit consequences of PV-specific *Dnmt1* deletion.

15. Third, while only leptin levels reached statistical significance, there certainly is a strong trend for increased ghrelin and GLP-1 levels in the mutant mice (Ext Data 3). Thus, the authors justification that the hypothalamic feeding circuit is not altered in Dmnt1 KO mice is not supported by their data; they have not performed a similar detailed analysis as they did in the cortex to justify this statement. Relatedly, I do not believe the authors have adequately shown that the changes in weight, hormone levels, etc., are not due to hypothalamic dysfunction and instead are solely due to an apathetic/anhodenic state as the authors claim. Instead, it seems reasonable (and this reviewer would argue likely) that some alteration in hypothalamic/feeding circuitry in part underlies the effects.

    We agree that with the current dataset we cannot exclude alterations in hypothalamic circuits. SnRNA seq of this brain regions would be indeed very interesting. However, such an expensive and time consuming analysis is beyond the scope of this study. Further, while we can't exclude a change in feeding circuitry, we saw no prominent changes in food consumption, for which further experiments are not justified for us. Also, in referral to the more detailed statement we gave to reviewer 2 (question 17) we want to point out that while other hormones also showed a trend towards increased levels in KO mice, there was no consistent functional trend: On the one hand, increased ghrelin and an overlap of PV and AgRP-expressing cells would support an increase in appetite regulation; on the other hand, leptin and GLP-1 suppress appetite, so increased concentrations of these hormones should entail a downregulation of food intake.

    We therefore revised the text to give more context on the investigated aspects of the hypothalamic feeding circuitry and clearly state that we cannot exclude hypothalamic defects.

16. I was somewhat confused about why the authors focused so heavily on the communication between PV cells and oligos. The authors state that PNNs require continuous support from oligos (lines 296-297), but there are no citations here and I am not familiar with this concept in the literature. Please provide additional references & context supporting the idea that oligodendrocyte-lineage cells play an important role in the formation and/or maintenance of PNNs, and thus PV cell function.

    We apologize for the lack of references, which we have included in the new version of the manuscript.

Minor points

17. I don't understand the point of Extended Data Tables #1 & 2, nor do I understand what they are displaying. The meaning of the single sentence in the text referencing this tables (lines 180-182) is unclear to me, and the Table legends don't provide any additional insight. I don't know what any of the columns in the table are referring to: where do these FDR, gene numbers & fold enrichment come from? There are no citations for any of the manuscripts/mouse lines that are referred to in the table. I recommend that the authors remove these tables as their purpose here is unclear & confusing.

    We thank the reviewer for their comment. We have revised the tables accordingly and added the citations.

18. In several graphs the y-axis does not start at 0 (Fig 2e, 6e, Ext Data Figs 1a, 2b, 2j, 3a-c, 6a) which can lead to confusion when comparing graphs. Please ensure that all relevant graphs start at the y = 0 intercept.

    All figures were accordingly revised.

19. Figure 1i: Please label each heatmap as Cont/KO in the figure.

    We added the missing labels in panel 1i.

20. Figure 4a: The authors need to show a UMAP color coded by WT and KO samples, which would be helpful for visualizing similarities (and differences) between the conditions. Are the WT & KO cells intermingled with each other (i.e., salt & pepper), or is there some separation between WT and KO cells within the clusters?

    We have included this in the respective extended data figure.

21. Extended Data Figure 4a: I don't understand why the authors overlayed the raw WT (pseudocolored green) and KO (red) tom+ cells in the right column. It is somewhat distracting because you can clearly see that the WT and Hom sections are not perfectly matched along the A-P axis, most notable in the second & third images. I cannot see the individual Tom+ cells in these small low mag images, so maybe replace these images with fewer sections along the A-P axis, allowing for larger images with greater resolution to see individual cells?

We removed the small pseudocolor overlays of the immunostainings, and showed representative A-P levels at higher magnification for both genotypes separately.

22. I find Figure 5D-E confusing to read. The left & right columns are not labeled in the figure panels; the PV-to-Olig direction should be clearly labeled in the figure. Also, it's not clear to me why the up and down-regulated pairs are separated into two panels. It makes more sense to have a single panel with upregulated-downregulated pairs for each direction on a sliding red-blue shaded scale, just like Fig 4E; that is a more standard way to present this type of heatmap. Are any of these predicted differences significant? The delta values look pretty small (highest is maybe 0.15), so is this a statistically significant difference?

We thank the reviewer for their feedback. Regarding panel labeling: We have added explicit panel titles and axis labels indicating the communication direction (PV interneurons → oligodendroglial cells and oligodendroglial cells → PV interneurons) directly within each figure panel.

Regarding the separation of up- and downregulated pairs: The two-panel layout was a deliberate design choice made on accessibility grounds. The default diverging red–blue–green scale used in CellChat's native visualizations is not colorblind-safe. We instead adopted two perceptually uniform, colorblind-accessible gradients (yellow–orange–red for increases and yellow–green–blue for decreases) which cannot be meaningfully combined into a single diverging scale without compromising their perceptual separability. We believe this representation is more accessible to a broader readership, including readers with color vision deficiency.

Regarding statistical significance of the Δ values: All L-R pairs displayed in Fig. 5d, e passed the per-condition permutation test significance threshold ($p < 0.05$) in at least one condition prior to computing Δ. This ensures that differences are computed only for interactions genuinely active in at least one biological state, rather than between two noise-level probabilities. The top 10 pairs shown per cell type were selected by ranking on |Δ| (the absolute difference in communication probability between KO and control), ensuring that only the most severely altered interactions are displayed. The magnitude of Δ is inherently constrained by the scale of CellChat communication probabilities, which are bounded and typically operate in the low decimal range even for well-established interactions; a Δ of ~0.15 therefore represents a substantial relative shift within this framework.

23. Figure 4A: What does the term 'Atypical excitatory neurons' mean? Is this taken from a previous publication, or just a term to describe cells that don't map onto other defined excitatory populations? They also show the most consistent and unidirectional change in the Fig 4E heatmap (whole row is blue), so some clarification in understanding this population would be helpful.

We thank the reviewer for this insightful comment. The term "atypical excitatory neurons" was a provisional, descriptive label used during cluster annotation to flag a population whose transcriptional signature did not conform to the canonical Layer 2–6a excitatory neuron identities present in the dataset; it was not derived from any prior publication, nor did it reflect a previously defined cell type. Specifically, the cluster did not sufficiently express canonical layer-specific markers (e.g., *Rorb* for L4, *Bcl11b*/*Fezf2* for L5/6 ET, *Cux1*/*2* for L2/3) to permit confident assignment to a known subtype using our reference databases (Tasic et al., 2018; Zeisel et al., 2018; Yao et al., 2023). We agree that this designation is uninformative and potentially misleading, and we have taken this opportunity to perform a thorough re-examination of this cluster's marker gene profile.

Re-analysis of the top differentially expressed genes reveals a highly coherent molecular identity fully consistent with neocortical Layer 6b (L6b) neurons. The cluster is marked by the expression of *Cplx3*, one of the most established and specific L6b markers in the mouse neocortex (Hoerder-Suabedissen & Molnár, 2013), alongside *Nr4a2* (Nurr1), a transcription factor recently shown to be functionally required for the differentiation of L6b neurons (Zhao et al., 2025). Additional markers including *Drd1* further corroborate this identity within the consensus transcriptomic definition for deep Layer 6 neurons (Kim et al., 2025). The significant downregulation of *Cux2* and *Rorb* confirms this is not a misassigned superficial population. The cluster has accordingly been re-labelled **"**Layer 6b neurons**"** throughout the revised manuscript.

The CellChat differential interaction data reveals that the uniformly negative values in the Layer 6b neuron row reflect a consistent reduction in outgoing signalling strength to all cortical and glial cell types in the KO condition. This may reflect indirect circuit-level effects of PNN loss. While PNNs in Layer 6 are predominantly associated with PV+ interneurons (Auer et al Ref 17 in the manuscript), the local inhibitory-excitatory balance modulated by PNN removal could alter the excitability and output of adjacent excitatory populations including L6b neurons.

Anna Hoerder-Suabedissen, Zoltán Molnár, Molecular Diversity of Early-Born Subplate Neurons, *Cerebral Cortex*, Volume 23, Issue 6, June 2013, Pages 1473–1483, https://doi.org/10.1093/cercor/bhs137

Kim, S.-J., Babola, T. A., & Lee, K. (2025). A consensus definition for deep layer 6 excitatory neurons in mouse somatosensory, visual, and motor cortex. *Cell Reports, 44*(9), 116167. https://doi.org/10.1016/j.celrep.2025.116167

Tasic, B. et al. (2018). Shared and distinct transcriptomic cell types across neocortical areas. Nature, 563, 72–78.

Yao, Z. et al. (2023). A high-resolution transcriptomic and spatial atlas of cell types in the whole mouse brain. Nature, 624, 317–332.

Zeisel, A., Hochgerner, H., Lönnerberg, P., Johnsson, A., Memic, F., van der Zwan, J., Häring, M., Braun, E., Borm, L. E., La Manno, G., Codeluppi, S., Furlan, A., Lee, K., Skene, N., Harris, K. D., Hjerling-Leffler, J., Arenas, E., Ernfors, P., Marklund, U., & Linnarsson, S. (2018). Molecular architecture of the mouse nervous system. Cell, 174(4), 999–1014.e22.

Zhao L, Tao YC, Hu L, Liu XY, Zhang Q, Zhang L, Ding YQ, Song NN. Satb2 and Nr4a2 are required for the differentiation of cortical layer 6b. Cell Death Discov. 2025 Mar 31;11(1):126. doi: 10.1038/s41420-025-02402-2. PMID: 40164579; PMCID: PMC11958660.

24. Extended Data 2d: I don't understand how the y-axis 'Path efficiency' is defined. The legend says that it's swimming trial distance divided by shortest route, which should always be a positive number > 1.0 since the swimming distance is always larger than shortest route. So I'm not sure why the y-axis ranges from 0-1, maybe there is some normalization factor after calculating this distance?

We want to thank the reviewer for raising this concern, our description was indeed the wrong way around. The path efficiency is defined as the shortest possible route divided by the actual swimming route, that is why it is in the range between 0-1. We corrected the respective figure legend.

25. I am not a behavior expert, can the authors please clarify which assays test apathy (lack of motivation) vs. anhedonia (lack of feeling pleasure), if that distinction is well established in the field. At times it wasn't clear to me which behavior is associated with which of these terms.

The discrimination between apathy and anhedonia in rodents can be challenging as behavioral tests often work with reward systems to motivate the animal to participate in experiments. As apathy constitutes a lack of motivation or goal-directed behavior (Jackson et al., 2022) and anhedonia describes the loss of the ability to experience pleasure or reward (Scheggi et al., 2018), both phenotypes can lead to similar outcomes of the same experiments. In the present study, we employed a battery of behavioral assays, including home-cage activity monitoring, nest building, coat state assessment, voluntary wheel running, female urine sniffing and social interaction. Our intention was to capture a broad spectrum of motivational and affective-like behaviors rather than to assign each test exclusively to apathy or anhedonia.

In the broadest sense, we interpret nest building, coat state, and daily activity as reflecting self-care, initiation, and general goal-directed behavior, which are commonly considered apathy-like domains. Female urine sniffing, social interaction, and voluntary wheel running were interpreted as reflecting reward sensitivity, incentive motivation, and engagement with naturally rewarding stimuli, often used as proxies for anhedonia-like behavior. However, we want to acknowledge that this distinction is not absolute. Many of these paradigms involve overlapping components of motivation ("wanting") and reward valuation ("liking"), and their interpretation can be influenced by additional factors such as locomotor activity, arousal, and social drive. For example, reduced investigation in the female urine sniffing or social interaction tests may reflect diminished reward processing but could also arise from reduced behavioral initiation. Similarly, voluntary wheel running depends on both motivational drive and the intrinsic rewarding properties of the activity. We would therefore refrain from making clear assignments of individual tasks which would also better reflect the ongoing debate in the field (Husain & Roiser, 2018).

Megan G. Jackson, Emma S. J. Robinson; The importance of a multidimensional approach to the preclinical study of major depressive disorder and apathy. Emerg Top Life Sci 9 December 2022; 6 (5): 479–489. doi: https://doi.org/10.1042/ETLS20220004

Simona Scheggi, Maria Graziella De Montis, Carla Gambarana, Making Sense of Rodent Models of Anhedonia, International Journal of Neuropsychopharmacology, Volume 21, Issue 11, November 2018, Pages 1049–1065, https://doi.org/10.1093/ijnp/pyy083

Husain, M., Roiser, J.P. Neuroscience of apathy and anhedonia: a transdiagnostic approach. Nat Rev Neurosci 19, 470–484 (2018). https://doi.org/10.1038/s41583-018-0029-9

26. Line 321-323: The authors stated that NRG1-ERBB4 from OPCs to PV cells is significantly diminished, but doesn't Fig 5E show that this is significantly increased? And how does this change the reference/citation about loss of trophic support?

We thank the reviewer for this careful observation. We corrected the original statement that contained an error in the directionality assignment. NRG1–ERBB4 signaling from OPCs to PV interneurons is indeed increased in KO animals, not decreased. The decrease in NRG1–ERBB4 is specific to the PV→Oligo direction, observed across all four oligodendrocyte subtypes examined.

Importantly, NRG1 and ERBB4 are co-expressed in both PV interneurons and oligodendrocyte lineage cells, enabling bidirectional signaling between these populations (Fazzari et al., 2010; Roy et al., 2007; Mei & Nave, 2014). The simultaneous decrease in PV→Oligo NRG1–ERBB4 alongside an increase in OPC→PV NRG1–ERBB4 is consistent with a compensatory upregulation, whereby reduced trophic output from PV cells toward oligodendrocytes triggers a reactive increase in glia-derived neuregulin signaling back onto PV cells. Such compensatory increases in NRG1/ERBB4 signaling in response to deficits in this pathway have been previously reported (Roy et al., 2007). We have revised the manuscript text accordingly to reflect this corrected and more nuanced interpretation.

Fazzari, P., Paternain, A. V., Valiente, M., Pla, R., Luján, R., Lloyd, K., Lerma, J., Marín, O., & Rico, B. (2010). Control of cortical GABA circuitry development by Nrg1 and ErbB4 signalling. Nature, 464(7293), 1376–1380.

Roy, K., Murtie, J. C., El-Khodor, B. F., Edgar, N., Sardi, S. P., Hooks, B. M., Benoit-Marand, M., Chen, C., Moore, H., O'Donnell, P., Brunner, D., & Corfas, G. (2007). Loss of erbB signaling in oligodendrocytes alters myelin and dopaminergic function, a potential mechanism for neuropsychiatric disorders. Proceedings of the National Academy of Sciences, 104(19), 8131–8136. https://doi.org/10.1073/pnas.0702157104

Mei, L., & Nave, K.-A. (2014). Neuregulin-ERBB signaling in the nervous system and neuropsychiatric diseases. Neuron, 83(1), 27–49. https://doi.org/10.1016/j.neuron.2014.06.007

27. The authors analyzed e-phys properties from V1, S1 and ALM to demonstrate that changes were not restricted to a single cortical area. But they only reported changes in PNNs in S1. Can the authors confirm that decreased PNNs/WFA staining around PV cells is also present in other cortical regions such as V1 and ALM?

We thank the reviewer for their comment. We have re-phrased the relevant sentence in the results to make it clear that the decrease in WFA signal intensity was observed across the whole cortex, and that the analysis of the number of PV cells enwrapped by PNNs were done in select areas. We have performed WFA stainings in somatosensory, somatomotor, and visual areas within parasagittal sections and, similar to the previous results, could not detect a change in PV-cell numbers enwrapped by PNNs.

28. I think the title is somewhat misleading since the authors haven't directly tested or shown that changes in the PV interneuron-glia coupling underlies these deficits, especially without confirming any of the DEGs or receptor-ligand changes.

We thank the reviewer for their input and have re-named the manuscript.

Dear Reviewers,

First of all, we want to that the reviewers for investing time in our manuscript and for the positive as well as constructive remarks and feedback.

Our responses in this document and well as wll newly inserted text in indicated by green font in the main manuscript.

Reviewers' comments:

Reviewer #1 (Remarks to the Author):

The authors have adequately addressed reviewers' comments and provided substantial clarifications regarding both methodological approaches and data interpretation.

However, in the revised manuscript they considerably expand the results concerning the non-cell-autonomous consequences of PV-specific DNMT1 deletion on excitatory neuronal populations. Specifically, they report an apparent overrepresentation of layer 4 pyramidal neurons and Tox+/Slc17a6+ corticospinal populations in KO mice.

These observations warrant additional discussion. In particular, it should be clarified in the manuscript why the enrichment of Tox+/Slc17a6+ populations most plausibly reflect activity- or circuit-dependent state transitions induced by altered inhibitory function, rather than expansion of a stable excitatory neuronal subtype (Artegiani et al., 2014:calcineurin/Nfat/Tox; Sahni et al., 2021:early specification of corticospinal neurons). Line: 463-464 "Single-nucleus RNA-seq uncovered an altered excitatory neuron composition" this is a confusing statement. Similarly, the downregulation of expression levels of activity dependent genes (Arc, c-Fos etc) should be discussed. There are several papers which propose that chronic network hyperactivity and other persistent activity paradigms result in sustained elevations in activity, which can lead to reduced activity-dependent gene expression (Corbett et al.,2018).

Overall, the study provides meaningful insights and represents a valuable contribution to the field. I therefore support publication.

We thank the reviewer for the overall assessment and for highlighting the need to clarify our interpretation of the Tox+/Slc17a6+ corticospinal-like populations. We fully agree that our snRNA-seq data do not demonstrate the expansion of a new, stable excitatory neuronal subtype, but rather point to activity- and circuit-dependent state transitions within established corticospinal-like neurons. The enrichment we observe is identified at the level of transcriptional neighborhoods by both proportion tests and Milo, and is accompanied by coordinated changes in stress-responsive regulons in deep-layer neurons.

In the revised manuscript, we therefore explicitly frame these Tox+/Slc17a6+ populations as candidate state shifts induced by altered inhibitory function, rather than as expansion of a distinct subtype. We now reference previous work on Tox/NFAT/calcineurin signaling and corticospinal specification (Artegiani et al., 2014; Sahni et al., 2021) to emphasize that Tox expression can mark specific developmental or activity-related states within corticospinal circuitry, and we avoid wording that could be read as implying the emergence of a novel excitatory class.

In line with this, we have revised the sentence "Single-nucleus RNA-seq uncovered an altered excitatory neuron composition" to read "Single-nucleus RNA-seq uncovered genotype-associated differences in the relative abundance of specific excitatory neuron populations", which more accurately reflects the state-level nature of the changes.

We appreciate the reviewer's suggestion to more explicitly discuss the downregulation of activity-dependent immediate early genes such as Arc and c-Fos. In our dataset, this suppression is restricted to excitatory neurons and occurs in the context of elevated tonic firing and reduced stimulus-locked, synchronous responses, as revealed by our in vivo recordings.

We now highlight in the Discussion that this pattern fits well with experimental evidence showing that chronic network hyperactivity or persistent activity paradigms can lead to sustained elevations in baseline activity but reduced activity-dependent gene induction. This interpretation is further consistent with very recent work showing that repeated neuronal hyperexcitation can drive persistent transcriptional and chromatin remodeling rather than simple sustained activation signatures (Murano, T., Hagihara, H., Tajinda, K. *et al.* Repetitive neuronal activation regulates cellular maturation state via nuclear reprogramming. *Nat Commun* **17**, 5881 (2026). https://doi.org/10.1038/s41467-026-74202-w), supporting the view that altered Arc/c-Fos/Nr4a expression reflects an adaptive activity-dependent state shift. In this framework, we interpret the downregulation of Arc/c-Fos/Nr4a factors as a desensitization of activity–transcription coupling in excitatory neurons exposed to chronically altered inhibitory control, rather than as a marker of neuronal loss.

Reviewer #2 (Remarks to the Author):

The authors have satisfactorily addressed my comments.
We thank the reviewer for this statement and for having reviewed our revised version.

Reviewer #3 (Remarks to the Author):

In this revised manuscript, the authors have made significant improvements that address many of the reviewer's comments. They have increased the methodological details & and included extended data files regarding their animal numbers, use of statistics & scRNAseq data that now allow reviewers/readers to more fully understand their analyses. The use of Milo to quantify proportional changes in cell types between WT and KO mice is a welcome addition. They have clarified the oligo nomenclature & how they define different subtypes. And I am satisfied with the authors explanations regarding hypothalamic function in the KO mice & their conclusion that changes in the hypothalamic PV+ cells & specific hypothalamic nuclei likely play a minor role at best in the observed phenotypes.

However, I still feel that there are important issues that were not adequately addressed by the authors' comments & manuscript changes that still have me question some of their conclusions.

Major points

1. A significant weakness of the manuscript continues to be that the authors still have not validated any of the putative proportional cell changes or DEGs in the manuscript. The change in PNN is a nice observation, but it does not directly address or confirm any of these predicted changes. And the addition of Milo analysis supports some of their conclusions with the proportional cell changes, but it is basically an additional (albeit valuable) way to reanalyze the same dataset. Without any validation of these predicted cellular and/or genetic changes, the manuscript in its current version is too speculative (especially extending to CellChat analyses). The authors cited several reasons for not validating any predicted changes, but I disagree with some of their points outlined below:

We agree that orthogonal validation of selected proportional changes and DEGs would be valuable. However, establishing and optimizing multiplex FISH panels in adult cortex is beyond the scope and timeframe of the present study, and immunostainings in complex cortical tissue come with substantial technical and interpretational limitations. Instead, we provide an independent, chromatin-level modality (single-nucleus ATAC-seq with eRegulon-based gene regulatory network inference) which supports the main snRNA-seq signatures: regulon activity changes are strongest in excitatory neurons across layers 4–6 and oligodendrocytes, and several stress-responsive regulons (Etv1, Egr family members, Nr4a factors) show coordinated changes in chromatin accessibility and target-gene expression that mirror the layer-specific DEG patterns.

a. I initially suggested that the authors validate the strong 3-4 fold increase in corticospinal neurons, as that large a change should be readily apparent via FISH or immuno. While Milo confirmed this change & is a welcome addition, it is still essentially a reanalysis of the same dataset. In response, the authors stated that establishing multiplexed FISH panels lies beyond the scope of the present study, and combining multiple markers makes panel design technically challenging. However, the authors clearly state in the revised manuscript that these corticospinal neurons are "…defined by the expression of Tox and Slc17a6 and the absence of Tle4 and Cux2…" (lines 283-284). Thus, there is a clear strategy to identify these cell types via FISH. It is not too difficult to perform FISH using 2-4 probes to confirm such a large predicted change in the proportion of cells between WT and KO mice.

We thank the reviewer for highlighting the robust proportional increase in corticospinal neuron-like nuclei in our snRNA-seq dataset and agree that an independent FISH confirmation of this shift would be highly informative. However, establishing and optimizing multiplex FISH panels for Tox, Slc17a6, Tle4 and Cux2 across multiple cortical regions in adult brain tissue is not feasible within the scope and timeframe of the present study, given the already extensive combination of in vivo electrophysiology, behavior, snRNA-seq, snATAC-seq, CellChat analysis and histology that we provide.

We therefore now explicitly describe the corticospinal neuron enrichment as a hypothesis-generating, non-cell-autonomous candidate effect rather than a central conclusion of the paper. In the revised Results and Discussion, we (i) emphasize that this proportional shift is identified by two independent statistical approaches (two-sample proportion tests and Milo) on the same snRNA-seq dataset, (ii) clearly state that no orthogonal validation has been performed, and (iii) highlight that future work will be required to confirm this enrichment with FISH.

b. There are also good markers that can be used for immunohistochemistry and/or FISH to confirm significant proportional changes in Layer 2-3 IT cells, Layer 4 cells & Layer 5-6 cells (Ext Data 7b). The authors find a significant decrease in astrocytes in KO mice, which can easily be assessed with GFAP immunos. I don't necessarily expect the authors to perform all of these suggestions, but there are well-established markers for many

of these cell types that can be used for FISH and/or immunos. None of this seems beyond the scope of this study; in fact, validation of these predictions is critical to ensure confidence in the predicted cellular & DEG changes.

We agree that there are well-established markers for several excitatory subclasses and astrocytes that could, in principle, be used for FISH or immunohistochemistry. Our initial rationale for not performing such experiments was that the proportional shifts in layer 2/3 IT, layer 4 and layer 5/6 neurons, as well as the modest change in astrocytes, are secondary observations and not the core focus of the present study.

We have nevertheless performed GFAP immunostaining in the somatosensory cortex and quantified area-normalized GFAP intensity. *Dnmt1* KO mice showed a significant reduction in GFAP signal intensity relative to controls (Extended Data Fig. 7e, f), consistent with the modest reduction in astrocyte representation identified by snRNA-seq. We have revised the manuscript accordingly and now include representative images and animal-level quantification. We interpret this result conservatively: GFAP is a state- and morphology-sensitive astrocytic intermediate-filament marker, and reduced GFAP immunoreactivity does not by itself demonstrate a proportional decrease in total astrocyte number. We therefore retain the cell-compositional finding as supportive, rather than treating GFAP staining as a definitive measure of astrocyte abundance. GFAP regulation is sensitive to astrocyte activation state, which further motivates this conservative interpretation.

We have also performed stainings of OLIG2-positive oligodendroglial cells and MBP, confirming that the overall abundance of oligodendroglial lineage cells and gross myelination are preserved in *Dnmt1* KO mice, in line with the snRNA-seq proportion tests and Milo results.

c. I also previously noted that the authors did not perform any validation of predicted DEGs from their scRNAseq dataset, and they still do not address this. In their response, the authors claim that the WFA staining "…directly confirms the biological endpoint of this signaling reorganization…", but that is extremely speculative that PNN changes are a "biological endpoint", and I would argue not a clear justifiable conclusion from their data. The authors make several strong claims in the revised manuscript about DEGs in the scRNAseq dataset that can easily be confirmed. To note a few, they claim broad upregulation of Fkpb5 and Zbtb16 in many cell types (lines 306-311), downregulation of Arc, Fos & Nra1-3 (lines 314-317), and a reduction of Pdgfc+ oligos (lines 367-370). Yet there is no orthogonal validation of any of these claims. There are very good antibodies for Arc and Fos that work for immunostaining, and upregulation of Fkbp5 and Zbtb16 in so many cell types should be very apparent with FISH. Again, I'm not expecting the authors to confirm every DEG, but they should validate some of these predictions via FISH and/or immunos, especially ones that are critical for additional analyses or important conclusions.

We appreciate the reviewer's concern about the lack of orthogonal validation for selected DEGs and agree that additional FISH or immunohistochemistry for markers such as Arc, Fos, Fkbp5, Zbtb16 and Pdgfc would further strengthen the study. In the present work, we have chosen to treat these DEGs as components of broader, hypothesis-generating transcriptional programs rather than as individual biomarkers. For example, the cortex-wide suppression of Fkbp5 and Zbtb16 across neurons and glia fits their established roles in stress-adaptive transcription and is supported by independent chromatin-level evidence: genotype-associated DEGs are significantly enriched among eRegulon target genes in most cell types, and several stress-responsive regulons (Etv1, Egr family members, Nr4a factors) show coordinated changes in chromatin accessibility and target-gene expression that mirror the layer-specific suppression of stress-related genes. Similarly, the downregulation of Arc, Fos and Nr4a1–3 in excitatory neurons is mechanistically consistent with our in vivo finding of reduced stimulus-locked, synchronized activity despite elevated tonic firing, and with the suppression of immediate early gene regulons in deep-layer and corticothalamic neurons.

We have revised the Discussion to explicitly frame these DEG patterns as hypothesis-generating and to emphasize that targeted FISH/IF validation of selected candidates (e.g. Arc/Fos, Fkbp5/Zbtb16, Pdgfc) will be an important goal for future work rather than a claim we can firmly establish here.

In addition, we agree with the reviewer that the characterization of WFA staining to "directly confirm the biological endpoint" was stronger than warranted, and we have revised this characterization accordingly. In the revised manuscript, we now state explicitly that the WFA findings provide tissue-level evidence consistent with, though not independent confirmation of, the PV–oligodendroglial reorganization predicted by CellChat. The histological findings (unchanged OLIG2+ cell numbers and MBP myelination, but reduced WFA-labeled PNN intensity) therefore serve as convergent evidence at the tissue level rather than as a direct endpoint of specific ligand–receptor changes.

2. I had numerous criticisms of the CellChat analyses in my original response, and Reviewer #1 had similar concerns stating "…CellChat analysis does not provide robust evidence for PV-glia coupling deficits… it is a very interesting finding that needs additional experiments to be verified…the authors should not over emphasize this statement". The authors' response of adapting the description & changing the title does not address either Reviewer #1 or my significant concerns about this CellChat analyses.

a. I will reemphasize that the authors did not provide any validation of these changes of signaling pathways genes, which both myself and Reviewer #1 requested/suggested. The authors' response is that the CellChat analyses "…are not intended to identify specific DEGs… rather to characterize shifts in predicted communication probabilities…". This statement is very odd as the whole CellChat analyses is based on differential expression of these signaling pathway genes between cell types & genotypes determined from their scRNAseq datasets (unless I'm misunderstanding this analysis works). This point only further emphasizes the need to confirm some of the predicted DEGs via FISH. Without some sort of confirmation, this entire CellChat analysis is purely speculative, and I agree with Reviewer #1 that it does not provide robust evidence for PV-glia coupling deficits. The authors claim in their rebuttal to this point that "We therefore consider the WFA data to be an appropriate functional validation of the overall conclusion", which is entirely speculative and not a valid statement. To conclude that WFA changes are a direct result and/or logical conclusion from the DEGs and CellChat analyses does not make sense to this reviewer.

We thank the reviewer for reiterating their concerns about the CellChat analysis. We agree that CellChat operates on ligand–receptor mRNA co-expression and therefore infers predicted communication probabilities rather than directly measuring synaptic connectivity or protein-level signaling. In the revised manuscript, we have accordingly: (i) clarified at the outset of this section that CellChat-derived communication probabilities are inferred from ligand–receptor mRNA co-expression, (ii) revised the wording throughout the section to consistently describe findings as "predicted" changes in communication probability rather than as direct changes in communication, input, or signaling strength, and (iii) added explicit qualifying language (e.g., "consistent with, but not proof of," "may reflect," "potential") around the specific conclusions the reviewer highlighted, including the NRG1-ERBB4 findings and the proposed PV-OPC decoupling. We have not removed the descriptive interpretation of these patterns, as we believe some framing of biological plausibility remains useful to the reader, but we have consistently flagged these interpretations as candidate, model-derived hypotheses rather than established signaling changes.

We continue to present CellChat as one convergent, hypothesis-generating line of evidence alongside snRNA-seq, snATAC-seq/eRegulon analysis, and histological PNN findings, rather than as stand-alone evidence for PV–glia coupling deficits. We have not performed additional orthogonal validation (e.g., FISH or immunohistochemistry) of individual ligand–receptor DEGs within the scope of this revision, and we explicitly note in the text that direct testing of these predicted signaling changes at the protein and synaptic level remains an important direction for future work.

b. I would argue that the revised manuscript now puts additional emphasis on this CellChat analyses and contains mischaracterizations/inaccuracies of the CellChat data that are not justified. In discussing the CellChat analyses (lines 327-382), the authors use terms like "reduction in neuron-to-neuron communication…reductions in outgoing/incoming communication/signaling…suppression of neuronal signaling…marked reduction of SST+ input onto PV cells…loss of excitatory/inhibitory inputs…functional uncoupling of PV-OPC axis". The last 2 paragraphs (lines 383-402) talk about Nrg1-Erbbr and GABA signaling being reduced/increased even though there is no evidence for this. Any conclusion about altered signaling, changes in communication, reductions/increases of inputs, etc., are entirely speculative. Without any orthogonal validation, the authors should not conclude that differential mRNA levels of these receptor-ligand pairs between cell types & genotypes definitively leads to changes in cell communication, signal strengths, changes in inputs, etc. Even if some of these putative changes are consistent with their optogenetic data, it is not valid to assume that these genetic changes definitively give rise to the altered signaling pathways they claim here (especially since we don't even know if these mRNA changes drive different protein levels). Thus, rather than de-emphasizing this CellChat analyses, the authors appear to have significantly increased their interpretation of this analysis. I do not believe this is justified without additional validation, and I do not believe that the authors have revised their manuscript in a way that accurately reflects the significant weaknesses and speculative nature of this analysis; rather, they have gone in the opposite direction.

We thank the reviewer for their detailed feedback and agree that several phrases in the original CellChat section implied more direct or definitive changes in signaling than the underlying transcriptomic data can support. We have revised the section throughout to consistently frame these findings as predicted probability scores rather than as direct changes in communication, input, or signaling strength. Specifically, the introductory sentence now states explicitly that CellChat-derived communication probabilities are inferred from ligand–receptor mRNA co-expression, and specific conclusions the reviewer identified as overstated are now qualified: the proposed PV-OPC relationship is described as "consistent with a potential uncoupling... rather than a general suppression of glial signaling," the NRG1-ERBB4 findings are framed as "a pattern consistent with, but not proof of, diminished neuregulin-mediated trophic output," and the GABAergic findings are now described in terms of signaling probability (i.e., "GABAergic signaling probability... was higher/lower") rather than as direct changes in signaling. We have also added a closing sentence noting that whether these predicted signaling changes are reflected at the protein or synaptic level remains to be explored in future work.

We wish to be transparent that these revisions are targeted rather than a full restructuring of the section: we have retained the underlying descriptive narrative connecting the CellChat results to our electrophysiological and histological findings, as we believe this framing is useful to the reader, while ensuring that each specific conclusion flagged by the reviewer is now presented as a candidate, model-derived hypothesis rather than a definitive claim. We continue to present CellChat as one convergent, hypothesis-generating line of evidence alongside snRNA-seq, snATAC-seq/eRegulon analysis, and histological PNN findings, rather than as stand-alone evidence for PV–glia coupling deficits.

3. The authors note that Pdgfc is a defining marker for the perineuronal oligodendrocytes, but they don't include this gene in the Fig 4B dot plot. Please add this gene to this panel to demonstrate that it demarcates this population. Additionally, I'd recommend generating oligo UMAP plots highlighting expression of Enpp6 and Pdgfc and including them in Ext Data Fig 8, this should nicely highlight the differential expression of these genes in oligo subtypes. I also think that the UMAP with the 4 defined oligo populations in their Response should be included in the manuscript, probably in Ext Data Fig 8. I'm somewhat skeptical that these perineuronal oligos represent a real oligo subtype since they are scattered throughout the UMAP (orange dots) and don't form any type of clear cluster, but hopefully visualizing expression of these marker genes will alleviate this concern.

We thank the reviewer for this suggestion. In the revised Extended Data Fig. 8, we now include (a–d) genotype-resolved UMAPs for each subtype; (e) a UMAP embedding of all four oligodendrocyte lineage subtypes, including perineuronal oligodendrocytes; (f–g) UMAP feature plots showing expression of *Enpp6* and *Pdgfc* across the lineage; (h) a dot plot of curated and data-derived marker genes across all four subpopulations; and (i) a volcano plot of differentially expressed genes in perineuronal oligodendrocytes of KO versus control mice.

Regarding the reviewer's concern that perineuronal oligodendrocytes do not form a discrete UMAP cluster: this is expected and consistent with prior molecular characterization of this population. Szuchet et al. (2011) demonstrated that perineuronal oligodendrocytes, despite being non-myelinating, "carry transcripts for all the major myelin proteins" (Table 1, Szuchet et al., 2011), and are therefore transcriptionally very similar to myelin-forming oligodendrocytes. The same study identified PDGF-CC (Pdgfc) as the defining molecular signature that sets perineuronal oligodendrocytes apart from myelinating oligodendrocytes, noting that this receptor-ligand axis precludes their response to the growth factors acting on myelinating cells. Our data recapitulate this pattern: *Pdgfc* expression is highly depleted in the myelinating oligodendrocytes (Extended Data Fig. 8h), while the broader transcriptional overlap with myelinating oligodendrocytes explains why perineuronal oligodendrocytes do not segregate into a spatially isolated region of the UMAP. We have added this effect in the manuscript, citing Szuchet et al. (2011) to clarify that perineuronal oligodendrocyte identity is defined by this specific transcriptional signature and by spatial proximity to neuronal somata, rather than by broad transcriptional divergence from other mature oligodendrocyte states.

Minor points

4. I had previously asked that the authors provide a citation(s) for their claim that "PNNs require continuous support from the oligodendrocyte lineage…" (lines 355-356), as this seems like an important claim to justify their analyses with oligo-PV communication. Despite the authors' response that they added references to address this, this line is still not cited in the revised manuscript. Please provide a citation supporting this claim.

We thank the reviewer for pointing this out. In the revised manuscript, we have added the appropriate references to support the statement that PNNs depend on continuous support from the oligodendrocyte lineage.

5. I appreciate that the authors added the requested UMAPs defined by WT and KO in Extended Data Fig 7. However, to better assess if the WT and KO cells are intermingled (i.e., salt & pepper) or there is spatial biases between cell types in specific clusters, the authors should label WT and KO cells in the same UMAP (just as they did with the Oligo UMAPs in Ext Data Fig 8) rather than separate the 2 genotypes into 2 separate UMAPs.

In response to the reviewer's suggestion, we have added combined UMAPs showing WT and KO cells within the same embedding for the major cortical cell types (new panels in Extended Data Figure 7). This allows visual assessment of whether WT and KO cells are intermingled ("salt & pepper") or show spatial biases within specific clusters, in addition to the separate WT/KO views.

6. I was unclear about which cell populations were significantly different in the Milo analyses. Were the same 8 cell populations that were significant in the proportionality test (Ext Data Fig 7A) also significant in Milo? Did the astrocyte change reach significance in Milo? Is every cell type represented by a dot in panel D significant? A clear statement about which cell types reached significance in Milo is needed.

We have now added a clear statement in the Results specifying which populations reached significance in Milo. In brief, while eight populations were significant in the two-sample proportion tests (Extended Data Fig. 7c), only five

of these showed significant neighborhood-level changes in Milo: layer 4 sensory neurons (enriched), layer 5–6 IT neurons (depleted), astrocytes (depleted) and the two corticospinal-like populations (enriched). Not every dot in panel D represents a significant change as only those that do have an additional black ring are significantly altered in both tests. We have now described this in the legend more explicitly.

7. While the authors have clarified what Ext Data Table 1 & 2 is showing, I still don't really understand the purpose of these tables. The authors claim that DEGs in their mice "…are significantly enriched in transcriptional signatures from mouse models displaying depression- and anxiety-like behaviors" (lines 189-192). However, there seems to be no difference in the % of genes, FDR or fold enrichment between mouse models that display depression/anxiety (marked with asterisk) vs. mouse lines that don't display depression/anxiety. Doesn't this actually refute the authors point, if many of these genes are up/downregulated in numerous mouse models that don't display these phenotypes? Thus, I don't agree with the authors statement above. Or at least it should be noted that the genes are also significantly enriched in many mutant mouse models that don't display these phenotypes, which again undercuts the authors' argument here. I still recommend that the authors remove these tables because I don't think they add anything to their story. If for some reason the authors do want to keep these tables, each mouse line needs to be cited with the relevant paper accordingly.

We thank the reviewer for this suggestion. We would like to clarify that the mouse lines in Extended Data Tables 1 and 2 with a documented depression- or anxiety-like phenotype are already cited in the main text (refs. 27–34), where we state that DEGs in Dnmt1 KO mice are enriched in transcriptional signatures with these phenotypes. Each mouse line in the tables is additionally annotated with its corresponding GEO accession number, which was already included in the revised submission and provides direct access to the deposited dataset and its associated publication record for every line, including those without a described depression/anxiety phenotype.

Given that both tables collectively comprise more than 20 mouse lines each, we consider it impractical to add an individual citation for every line beyond what is already provided. We believe the existing combination of in-text citations for the phenotype-relevant lines and GEO accessions for the full dataset already allows readers to trace each line to its source study and original characterization. We have, however, clarified this annotation scheme directly in the table legend to make it more transparent to readers.

Reviewers' comments:

Reviewer #1 (Remarks to the Author):

The authors have adequately addressed all the points raised during the previous round of review, and the manuscript has been significantly improved. Only a few minor issues remain, which should be straightforward to address:

1. In Figure 7a,b, Olig2 (magenta) cannot be clearly distinguished from tdTomato (red). Please use more distinct pseudocolors for these two signals.

We thank the reviewer for this comment. We have adjusted the pseudocolors in the corresponding subfigures to more easily distinguish the OLIG2 signal from the tdTomato signal.

2. In Supplementary Figure 7, the most prominent GFAP immunoreactivity appears to originate from fibrous astrocytes, in which GFAP is strongly expressed. By contrast, the weaker GFAP signal from astrocytes throughout the cortical layers is not readily discernible. If images at this magnification were used to quantify GFAP density, the measurements may be dominated by strongly labelled fibrous astrocytes and may not accurately represent GFAP expression within the cortex. Please perform or present the analysis using higher-magnification, region-specific images, or provide higher-magnification representative images that allow the cortical staining to be properly evaluated.

We thank the reviewer for this important observation regarding the prominent GFAP immunoreactivity from fibrous astrocytes. We agree that if the entire field of view were quantified indiscriminately, the measurements would be skewed. To ensure our measurements accurately represented GFAP expression within the cortex, our quantification was strictly confined to defined regions of interest (ROIs). Specifically, we localized the somatosensory cortex by cross-referencing with the mouse brain atlas and restricted our analysis exclusively to the grey matter. White matter structures, such as the corpus callosum and marginal zones where fibrous astrocytes are most prominent, were explicitly excluded from the analysis presented in this study. Furthermore, this region-specific quantification was performed on a robust dataset of six mice (three KO and three WT) with eight sections per genotype, yielding a total of 16 analyzed sections. We have added this information to the methods.

Further, to fully address your concern and clarify exactly where the cortical staining was evaluated, we have updated the representative microphotographs in Supplementary Figure 7 to explicitly display the ROIs used for our analysis. This visual addition demonstrates that the regions measured were confined exclusively to the cortical grey matter, preventing the strongly labelled fibrous astrocytes from dominating the measurements and allowing the cortical staining to be properly evaluated.

3. In Supplementary Figure 11, the tdTomato signal does not appear representative of PV interneuron labelling. Almost the entire section exhibits strong red fluorescence, which cannot reasonably be attributed solely to the somata and processes of PV neurons. Please replace this image with a more representative, higher-magnification image in which tdTomato-positive PV interneurons can be clearly identified.

More generally, the newly added immunohistochemical findings would be presented more convincingly using higher-magnification images, similar to those shown in Figure 7d,e.

Subject to these minor revisions, I endorse the manuscript for publication.

We thank the reviewer for this comment. We have adjusted the image in Supplementary Figure 11 to improve the visibility of the cortical tdTomato signal, allowing the tdTomato-positive PV interneurons to be more clearly identified.

Reviewer #3 (Remarks to the Author):

The authors have done a good job addressing the reviewers' comments and improving the manuscript. They provide new immunohistochemistry experiments to confirm some of their initial observations, and their new scATAC data with Regulon/Scenic+ analyses provides additional support for many of their original insights about changes in mRNA levels and predicted alterations of cell-cell communications. They have satisfied my other concerns and adjusted portions of the manuscript to more accurately portray the speculative nature and/or hypothesis-generating aspects of the analyses. I think this work is an important contribution to the field and I have no further comments.

We thank the reviewer for their time and positive feedback. We are glad that our revisions and new experiments successfully addressed your concerns.
