# Supplementary material for: DNMT1 function in cortical PV interneurons modulates cortical network activity, mood disorder‑related behavior, and perineuronal nets
Source: Commun Biol. 2026 Oct 8;9:1316. doi: 10.1038/s42003-026-11079-x (PMC13650159; doi:10.1038/s42003-026-11079-x)
Supplement: Supplementary file 2 [file 42003_2026_11079_MOESM2_ESM.pdf]

DNMT1 function in cortical PV interneurons modulates cortical network activity, mood disorder-related behavior, and perineuronal nets

Jenice Linde[1,2§], Can Bora Yildiz[1,2§], Katharina Vöhringer[1,2], Gerion Nabbefeld[2,3], Severin Graff[2,4], Daniel Pensold[1], Julia Reichard[1,2], Marie Hermanns[1], Laura Koch[1], Magdalena Müller[1,2], Ayushi Tanwar[1], Christoph Weber-Hamacher[5], Ivan G. Costa Filho[6], Thomas Stiehl[7], Marc Spehr[2,5], Björn Kampa[2,3], Anja Urbach[8], Simon Musall[2,4‡], Geraldine Zimmer-Bensch[1,2 ‡*]

[1] Department for Neuroepigenetics, Institute for Biology II, RWTH Aachen University

[2] Research Training Group RTG2416 "MultiSenses – MultiScales", RWTH Aachen University

[3] Department for Molecular and Systemic Neurophysiology, Institute for Biology II, RWTH Aachen University

[4] Institute of Biological Information Processing (IBI-3) Bioelectronics, Forschungszentrum Jülich

[5] Department of Chemosensation, Institute for Biology II, RWTH Aachen University

[6] Institute for Computational Genomics, RWTH Aachen University Hospital

[7] Institute for Computational Biomedicine and Disease Modeling, RWTH Aachen University Hospital

[8] Department of Neurology, Jena University Hospital

[§, ‡] Equal contribution

* Corresponding author

Supplementary Legends

Supplementary Figure 1: In vivo electrophysiological recordings in V1 and S1 suggest similar alterations in network processing across the cortex.

Recordings were conducted in the primary visual (V1, panels a–c) or somatosensory (S1, panels d–f) cortex of awake mice (also see scheme in Fig. 1a), using either low-frequency noise patterns for visual stimulation or air puffs for tactile stimulation (n = 8 recordings from 2 mice per genotype). **(a–c)** Quantification of the traces shown in Figure 1e. **(a)** Time of peak firing rate in response to visual stimulation increased in *Pvalb-Cre/tdTomato/Dnmt1 loxP*^{2} (KO) mice compared to *Pvalb-Cre/tdTomato* (control) mice (Wilcoxon *rank-sum* test: $p = 2 \times 10^{-26}$). **(b)** Stimulus responses of mice were less precise as reflected in an increased width at half maximum height (Wilcoxon *rank-sum* test: $p = 1.4 \times 10^{-7}$). **(c)** The area under the receiver operator characteristic curve (AUC) implies that in response to stimulation, fewer cells were involved in the cortical processing of KO mice (n_{Ctrl} = 31%, n_{KO} = 22%; Wilcoxon *rank-sum* test: $p = 6.1 \times 10^{-4}$) with a lower specificity (Wilcoxon *rank-sum* test: $p = 1.4 \times 10^{-4}$). **(d–f)** Local field potentials (LFP) and current source densities (CSD) of control and *Dnmt1* KO mice recorded in S1 in response to tactile stimulation at time point 0. **(d)** LFPs and CSDs of control and KO mice across the whole depth of the cortex. White lines indicate tactile stimulus onset. **(e)** LFPs in S1 in response to tactile stimulation at time point 0, averaged across cortical layers. Shading shows SEM across recordings. **(f)** LFPs in S1 in response to tactile stimulation with concurrent optogenetic activation of PV interneurons. Panels a–c display individual data points with median (center line) and interquartile range (upper and lower lines). Ctrl = control, CSD = current source density, KO = knockout, LFP = local field potential, S1 = primary somatosensory cortex, V1 = primary visual cortex; * $p < 0.05$, ** $p < 0.01$, *** $p < 0.001$, **** $p < 0.0001$.

Supplementary Figure 2: Learning and memory formation are intact in *Dnmt1*-deficient mice, also in a sensory processing-dependent task.

(a-d) Learning and memory formation of *Pvalb-Cre/tdTomato* control (Ctrl) and *Pvalb-Cre/tdTomato/Dnmt1 loxP*^{2} (KO) mice were tested in a Morris water maze (MWM), showing similar performances of both genotypes (n = 8 mice per genotype). **(a)** Task schematic: mice were introduced into the MWM from randomly alternating sites and had to find a hidden platform based on visual landmarks. **(b)** Mice of both genotypes similarly improved their success rate across sessions (mean ± SEM of mice; Binomial test: $p = 0.716$). **(c)** Mice of both genotypes similarly improved their swimming distances (Two-Way ANOVA (Repeated Measures): $p_{(Genotype)} = 0.63$, $p_{(Sessions)} = 1.3 \times 10^{-22}$, $p_{(Genotype\ x\ Sessions)} = 0.92$). **(d)** Path efficiencies, i.e., the respective shortest route to the platform divided by the actual swimming distance per trial (visualized in panel a), did not differ in knockout mice (Two-Way ANOVA (Repeated Measures): $p_{(Genotype)} = 0.11$, $p_{(Sessions)} = 7.9 \times 10^{-15}$, $p_{(Genotype\ x\ Sessions)} = 0.36$). **(e–j)** Control and KO mice were trained on a visual evidence accumulation task. **(e)** Task schematic: head-fixed mice were trained on reporting the side where the higher amount of stimuli was presented on a screen by licking a corresponding water nozzle. In expert mice, electrophysiological recordings using Neuropixels probes were conducted in the anterolateral motor cortex (ALM) during multiple sessions. **(f)** Learning curves for a visual evidence accumulation task (LME model: $n_{(Ctrl)} = 5$, $n_{(KO)} = 7$, $p_{(Genotype)} = 0.28$) and **(g)** corresponding psychometric curves of individual animals (LME model: $n_{(Ctrl)} = 5$, $n_{(KO)} = 7$, $p_{(Genotype)} = 0.95$) showed no genotype-dependent effects. **(h–j)** Electrophysiological recordings in ALM of mice trained on a visual evidence accumulation task showed similar changes in cortical activity as in V1. **(h)** Firing rates of ALM neurons in response to visual stimulation in trained mice showed weaker signals

in knockout compared to control mice. **(i)** Baseline-corrected firing rate of all PFC neurons that were inhibited during optogenetic activation of PV interneurons. PV-induced inhibition was significantly weaker in knockout mice compared to control animals. **(j)** Optogenetic activation of PV cells in the ALM likewise impaired decision-making in control (upper plot; LME model: n = 3, $p_{(\text{Genotype})}$ = 1.13x10^{-15}) and knockout mice (lower plot; LME model: n = 2, $p_{(\text{Genotype})}$ = 4.06x10^{-15}). Panels h–i display individual data points with median (center line) and interquartile range (upper and lower lines). Ctrl = control, KO = knockout, LME = linear mixed-effects; * $p < 0.05$, ** $p < 0.01$, *** $p < 0.001$, **** $p < 0.0001$.

Supplementary Figure 3: The excess body weight of *Pvalb-Cre/Dnmt1*-knockout mice is likely not appetite-based.

(a) *Pvalb-Cre/tdTomato* (control) and *Pvalb-Cre/tdTomato/Dnmt1 loxP*^{2} (knockout) mice displayed similar latencies to fall from a rotating rod across trials (mean ± SEM; see Fig. 2c for comparison of individual animals). **(b)** Knockout mice showed a decreased latency to fall throughout all trials of a wirehang test (mean ± SEM; see Fig. 2d for comparison of individual animals). **(c)** Group-housed control and knockout mice had the same food consumption rates (n = 8 mice per genotype). **(d)** In single-housed males ($n_{(\text{Control})}$ = 4, $n_{(\text{Knockout})}$ = 6) both the absolute feed intake as well as the absolute water intake were not altered in *Pvalb-Cre/Dnmt1*-KO mice (upper panels). **(e)** In contrast, the respective intake relative to the animals' body weight showed a significant reduction in food and water intake in knockout mice. **(f)** Blood levels of various appetite-regulating hormones were unaltered by the *Dnmt1*-knockout (n = 6 biological replicates per genotype). Prominently, only leptin levels were increased in knockout mice. Panels a and d display individual data points with median (center line) and interquartile range (upper and lower lines). * $p < 0.05$, ** $p < 0.01$, *** $p < 0.001$, **** $p < 0.0001$.

Supplementary Figure 4: PV cell distribution and quantity were not altered in *Pvalb-Cre/tdTomato/Dnmt1 loxP*^{2} (knockout) mice.

(a) Cell distribution of tdTomato-labeled PV interneurons was unaffected by the *Dnmt1* knockout. Each row shows a subcortical region relevant in appetite regulation. Left: Schemes visualizing the investigated brain areas. Right: Overlay of PV cell distribution found in *Pvalb-Cre/tdTomato* (control; gray dots) and knockout (red dots) mice. Scale bars = 500 µm. **(b)** Representative pseudocolor images of selected coronal brain sections from control and knockout mice. The green color indicates tdTomato-labeling in control mice, red color denotes tdTomato-positive cells in knockout mice. The panels show a subset of the brain regions depicted in (a). Scale bars = 500 µm. **(c)** Red marked regions in the illustrations on the left-hand side show areas of interest for appetite regulation, where PV cells were counted in coronal slices from different animals. Quantitative comparisons of PV interneurons in these regions revealed no differences between control and knockout animals (graphs on the right-hand side) (box plots: median, interquartile range, Tukey; $n_{(\text{Control})}$ = 4 biological replicates, $n_{(\text{Knockout})}$ = 3 biological replicates).

Supplementary Figure 5: PV cell identity in the hypothalamus is unaltered in *Pvalb-Cre/tdTomato/Dnmt1 loxP*^{2} (knockout) mice and does not indicate a connection between PV interneuron activity and appetite regulation.

(a) β-Endorphin immunostaining in the arcuate nucleus of the hypothalamus was conducted to identify Proopiomelanocortin-expressing (POMC) cells. The tdTomato-labeling (white arrows) did not overlap with the β-endorphin staining in both *Pvalb-Cre/tdTomato* (control) and knockout mice (scale bar = 50 µm). **(b)** GABA immunostaining in the anterior hypothalamic nucleus of a control mouse showed a partial overlap of tdTomato- and GABA-labeling (white arrows). Immunostainings indicated that only a fraction of the tdTomato-positive PV interneurons in the hypothalamus express GABA and could thus be attributed to the group of AgRP-expressing cells, while other PV cells were not positive for GABA (empty arrows; scale bar = 50 µm; N = 1 mouse per genotype)

Supplementary Figure 6: Food deprivation in the novelty-suppressed feeding test affected mice of both genotypes similarly.

(a) *Pvalb-Cre/tdTomato/Dnmt1 loxP*^{2} (knockout) and *Pvalb-Cre/tdTomato* (control) mice (n = 10 mice per genotype) lost similar amounts of weight after food deprivation. **(b)** In their home cages, mice of both genotypes consumed similar amounts of the pellet in a 5-min time window. Panels display individual data points with median (center line) and interquartile range (upper and lower lines).

Supplementary Figure 7: Proportions of identified clusters within the neocortex of *Pvalb-Cre/tdTomato* (Ctrl) and *Pvalb-Cre/tdTomato/Dnmt1 loxP*^{2} (KO) mice.

(a) UMAP embedding of all cells within the snRNA-seq dataset, split by genotype (Ctrl, gray; KO, teal). **(b)** Relative number of cells per cluster, normalized to total cells per genotype, were significantly altered for select excitatory neuron subtypes and astrocytes (two-sample proportion test; Benjamini–Hochberg correction; $p_{\text{adj, L2/3 IT}} = 0.0082$, $p_{\text{adj, L4}} = 0.0142$, $p_{\text{adj, L5/6 IT}} = 7.0582\text{E-}12$, $p_{\text{adj, Deep-layer ET}} = 0.0001$, $p_{\text{adj, Corticospinal Type I}} = 1.8147\text{E-}30$, $p_{\text{adj, Corticospinal Type II}} = 7.7081\text{E-}17$, $p_{\text{adj, Astrocytes}} = 0.0417$, $p_{\text{adj, Endothelial}} = 2.0998\text{E-}08$). * $p_{\text{adj}} < 0.05$. **(c)** Milo differential abundance (DA) analysis results displayed as a beeswarm plot of log fold change (KO vs. Ctrl) per neighborhood, grouped by cell type. Each dot represents one k-nearest-neighbor neighborhood. **(d)** Integrated visualization of the two-sample proportion analysis and Milo DA results. The x-axis shows the relative proportion difference (%) between KO and Ctrl mice on a pseudo-log scale while the y-axis shows the Milo median log fold change per cluster. Bubble size reflects the number of significant Milo neighborhoods within a given cell cluster. The presence of a black ring indicates clusters that are significantly altered in KO mice in both the two-sample proportion test as well as the Milo neighborhood analysis which are astrocytes, layer 4 sensory neurons, layer 5/6 IT neurons, corticospinal neurons (type I) and corticospinal neurons (type II). **(e, f)** Representative images of GFAP immunostaining in the somatosensory cortex of control **(e)** and *Dnmt1* KO **(f)** mice. **(g)** Quantification of area-normalized GFAP intensity in the somatosensory cortex, showing reduced GFAP signal intensity in *Dnmt1* KO mice relative to controls. Each point represents one animal; bars indicate mean ± S.E.M. (nested t-test, p = 0.035; N = 3 biological replicates with n = 8 slices per genotype). Scale bars: 1000 µm (left), 500 µm (right). ET: Extratelencephalic. IT: Intratelencephalic. PT: Pyramidal tract. PV: Parvalbumin. SST: Somatostatin. VIP: Vasointestinal peptide.

Supplementary Figure 8: Oligodendrocyte lineage subtype distributions and transcriptomic alterations in perineuronal oligodendrocytes of *Dnmt1* KO mice.

(a–d) UMAP embeddings highlighting control (gray) and KO (teal) nuclei for each oligodendrocyte lineage subtype: **(a)** oligodendrocyte precursor cells, **(b)** newly formed oligodendrocytes, **(c)** myelinating oligodendrocytes, and **(d)** perineuronal oligodendrocytes. **(e)** UMAP embedding of the four oligodendrocyte lineage subtypes identified within the oligodendroglial compartment, colored by cluster identity. No apparent genotype-driven shifts in UMAP distribution were observed across any of the four subtypes. **(f, g)** UMAP feature plots showing expression of *Enpp6* **(f)** and *Pdgfc* **(g)** across the oligodendrocyte lineage. *Enpp6* is a marker for OPC-to-oligodendrocyte transition and thus enriched in newly formed oligodendrocytes, while *Pdgfc* marks perineuronal oligodendrocytes, distinguishing these populations from myelinating oligodendrocytes and oligodendrocyte precursor cells. **(h)** Dot blot of marker genes across the identified oligodendroglial subpopulations. **(i)** Volcano plot depicting differentially expressed genes in perineuronal oligodendrocytes of KO mice compared to control (Wilcoxon rank-sum test; $|\log_2 FC| \geq 0.26$, $p_{adj} < 0.05$). All significant changes were downregulated, with affected genes implicated in stress-resilience (*Sgk1*, *Fkbp5*), ECM remodeling (*Htra1*, *Daam2*, *Rcan2*), and structural programs (*Zbtb16*, *Bcl2l1*, *Tmod2*, *Mobp*). Dashed lines indicate the $|\log_2 FC|$ (0.26) and significance ($p_{adj} = 0.05$) thresholds.

Supplementary Figure 9: Oligodendrocyte lineage dynamics reveal a decreased transition from newly formed oligodendrocytes (NFOLs) to perineuronal oligodendrocytes in *Pvalb-Cre/tdTomato/Dnmt1 loxP*^{2} (KO) mice.

(a) A simple compartmental model of oligodendrocyte lineage progression dynamics, which considers four oligodendroglial populations in sequence: OPCs, *Pdgfc*-negative NFOLs (*Pdgfc*$^-$ NFOL), *Pdgfc*-positive NFOLs (*Pdgfc*$^+$ NFOL, i.e., perineuronal oligodendrocytes), and myelinating oligodendrocytes (MOL). **(b)** *Pdgfc*$^-$ NFOL/OPC ratio was significantly increased in KO mice, the *Pdgfc*$^+$ NFOL/Pdgfc$^-$ NFOL ratio was significantly reduced, while the MOL/*Pdgfc*$^-$ NFOL ratio remained unchanged between genotypes (two-sided Wald tests on log-ratios, Holm correction). neg: *Pdgfc*-negative. OPC: Oligodendrocyte precursor cells. pos: *Pdgfc*-positive.

Supplementary Figure 10: Neocortical myelination is not altered in the *Pvalb-Cre/tdTomato/Dnmt1 loxP*^{2} (KO) mice.

(a) Representative microphotographs of myelin basic protein (MBP) immunostaining in parasagittal brain sections from control and *Dnmt1* KO mice. **(b)** Quantification of the MBP signal in control and *Dnmt1* KO mice reveals no significant differences across the motor, somatosensory, and visual cortices. Dots and shading represent the mean of individual biological replicates ± SEM (linear mixed model; $N = 3$ biological replicates with $n = 3$ slices per genotype). Scale bar = 1000 µm.

Supplementary Figure 11: Loss of DNMT1 in PV interneurons reduces perineuronal net (PNN) levels but does not change the proportion of PV interneurons enwrapped by PNNs in the neocortex of *Pvalb-Cre/tdTomato/Dnmt1 loxP*^{2} (KO) mice.

(a–d) A laminar analysis of Wisteria floribunda agglutinin (WFA)-labeled PNNs in the somatosensory cortex in coronal sections reveals a significant reduction of PNNs in layers 2–5 of the *Dnmt1* KO mice (nested *t*-test, $p_{L2/3/4} = 0.041$; $p_{L5} = 0.042$; $N = 3$ biological replicates with $n = 6$ slices per genotype). **(e)** Representative parasagittal section stained for PNNs using WFA showing the localization of the visual, somatomotor, and somatosensory areas used for

analysis. **(f–h)** Proportion of PV interneurons enwrapped by PNNs in brain slices of KO mice is unchanged in **(f)** the visual areas (paired Student's *t*-test, $p = 0.600$; $N = 3$ biological replicates with $n = 5$ slices per genotype), **(g)** somatomotor areas (paired Student's *t*-test, $p = 0.440$; $N = 3$ biological replicates with $n = 6$ slices per genotype), and **(h)** the somatosensory areas (paired Student's *t*-test, $p = 0.277$; $N = 3$ biological replicates with $n = 4$ slices per genotype). Panels a–d display individual data points with median (mid-line) while panels f-h display individual data points with median (mid-line) and interquartile range (upper and lower lines). Scale bar = 1000 μm.

Supplementary Table 1: *Dnmt1*-knockout in PV interneurons upregulates genes concordantly induced in other mouse models.

Mouse lines marked with an asterisk have been found to express behavioral alterations associated with depression and/or anxiety. GEO accession numbers are provided for all mouse lines to allow direct retrieval of the source dataset and associated publication. KD: Knockdown. KO: Knockout. OE: Overexpression.

Mouse lines	Nr. of genes	Ref. genes	% of ref. genes	Enrichment FDR	Fold Enrichment
Htt* Mutation (HTT exon1 142Q) mouse GSE3248	**21**	**340**	**6.20%**	**2.70E-06**	**5**
Pmp22 KO mouse GSE1947	16	263	6.10%	9.90E-05	4.9
Pmp22 OE mouse GSE1947	15	258	5.80%	3.20E-04	4.7
Ache AChE-S mutation mouse GSE31458	16	302	5.30%	3.30E-04	4.3
Pmp22 KO mouse GSE1947	13	242	5.40%	1.00E-03	4.3
Htt* Mutation (HTT exon1 18Q) mouse GSE3248	**14**	**273**	**5.10%**	**9.00E-04**	**4.1**
Dysf Deficiency mouse GSE2507	13	254	5.10%	1.50E-03	4.1
Nfatc3 OE mouse GSE58960	14	279	5.00%	1.00E-03	4.1
Tlr4 mutation mouse GSE476	21	424	5.00%	6.30E-05	4
Emx2 Deficiency mouse GSE10216	15	305	4.90%	8.90E-04	4
Dmd* Deficiency mouse GSE897	**17**	**346**	**4.90%**	**3.50E-04**	**4**
Rps6ka3* knockout mouse GSE22137	**17**	**350**	**4.90%**	**3.60E-04**	**3.9**
Ache* AChE-R over-expressor mutation mouse GSE31458	**15**	**309**	**4.90%**	**9.00E-04**	**3.9**
Sox9 OE mouse GSE34060	16	341	4.70%	8.90E-04	3.8
Hras mouse GSE3530	15	323	4.60%	1.20E-03	3.8
CHRNA9 KO mouse GSE18567	16	346	4.60%	8.90E-04	3.7
Atxn7 OE mouse GSE3634	19	419	4.50%	3.30E-04	3.7
Prmt5 KO mouse GSE45269	15	335	4.50%	1.60E-03	3.6
Sod1* G93A mutation mouse GSE10953	**19**	**462**	**4.10%**	**8.90E-04**	**3.3**
Dicer1* KO mouse GSE16923	**17**	**426**	**4.00%**	**1.90E-03**	**3.2**

Supplementary Table 2. *Dnmt1*-knockout in PV interneurons downregulates genes concordantly repressed in other mouse models.

Mouse lines marked with an asterisk have been found to express behavioral alterations associated with depression and/or anxiety. GEO accession numbers are provided for all mouse lines to allow direct retrieval of the source dataset and associated publication. KD: Knockdown. KO: Knockout. OE: Overexpression.

Mouse line	Nr. of genes	Ref. genes	% of ref. genes	Enrichment FDR	Fold Enrichment
Prmt1 KD mouse GSE47214	51	502	10.20%	1.50E-23	6.5
Cyfip1 OE mouse GSE40852	14	164	8.50%	4.90E-05	5.5
Htt* Gain of function by knock in of htt cag repeat length-7/16 mouse GSE26001	**14**	**167**	**8.40%**	**4.90E-05**	**5.4**
Prmt8 KD mouse GSE47214	35	433	8.10%	1.30E-12	5.2
Eomes KO mouse GSE43387	22	273	8.10%	1.90E-07	5.2
Nrl KO mouse GSE4051	21	267	7.90%	5.70E-07	5
Top2b KO mouse GSE5458	16	220	7.30%	4.90E-05	4.7
Pten KO mouse GSE64303	20	283	7.10%	4.90E-06	4.5
Ppard* KO mouse GSE16048	**15**	**216**	**6.90%**	**1.40E-04**	**4.4**
FOXO3 KO mouse GSE18326	23	339	6.80%	1.30E-06	4.3
Apod KO mouse GSE28643	21	311	6.80%	4.90E-06	4.3
Htt* OE mouse GSE3583	**18**	**278**	**6.50%**	**4.90E-05**	**4.1**
Setdb1* KO mouse GSE40296	**19**	**303**	**6.30%**	**4.90E-05**	**4**
Nrl Deficiency mouse GSE4051	17	275	6.20%	1.40E-04	4
Atxn1* Knock-in mouse GSE2867	**17**	**279**	**6.10%**	**1.60E-04**	**3.9**
Mef2d KD mouse GSE47150	20	338	5.90%	4.90E-05	3.8
Nrl Deficiency mouse GSE4051	20	346	5.80%	6.40E-05	3.7
Htt* Knock-in mouse GSE19780	**19**	**331**	**5.70%**	**1.20E-04**	**3.7**
Cstb* KO mouse GSE47516	**21**	**384**	**5.50%**	**7.60E-05**	**3.5**
Dicer1* KO mouse GSE44162	**20**	**376**	**5.30%**	**1.60E-04**	**3.4**

Supplementary Video legends

Supplementary Video 1: Three-dimensional reconstruction of a PV interneuron and its perineuronal net.

Rotating 3D rendering of a parvalbumin-positive (PV) interneuron (red) from a *Pvalb-Cre/tdTom* (Ctrl) mouse, surrounded by its perineuronal net (PNN) labeled with Wisteria floribunda agglutinin (WFA, green). The video illustrates the spatial relationship and enwrapping pattern of the PNN around the soma and proximal dendrites.

Supplementary Video 2: Three-dimensional reconstruction of a *Dnmt1*-deficient PV interneuron and its perineuronal net.

Rotating 3D rendering of a PV interneuron (red) from a *Pvalb-Cre/tdTom/Dnmt1 loxP*[2] (KO) mouse and its PNN labeled with WFA (green). The video highlights the perturbations in PNN organization and coverage compared to the control condition shown in Supplementary Video 1.

Supplementary Fig. 1

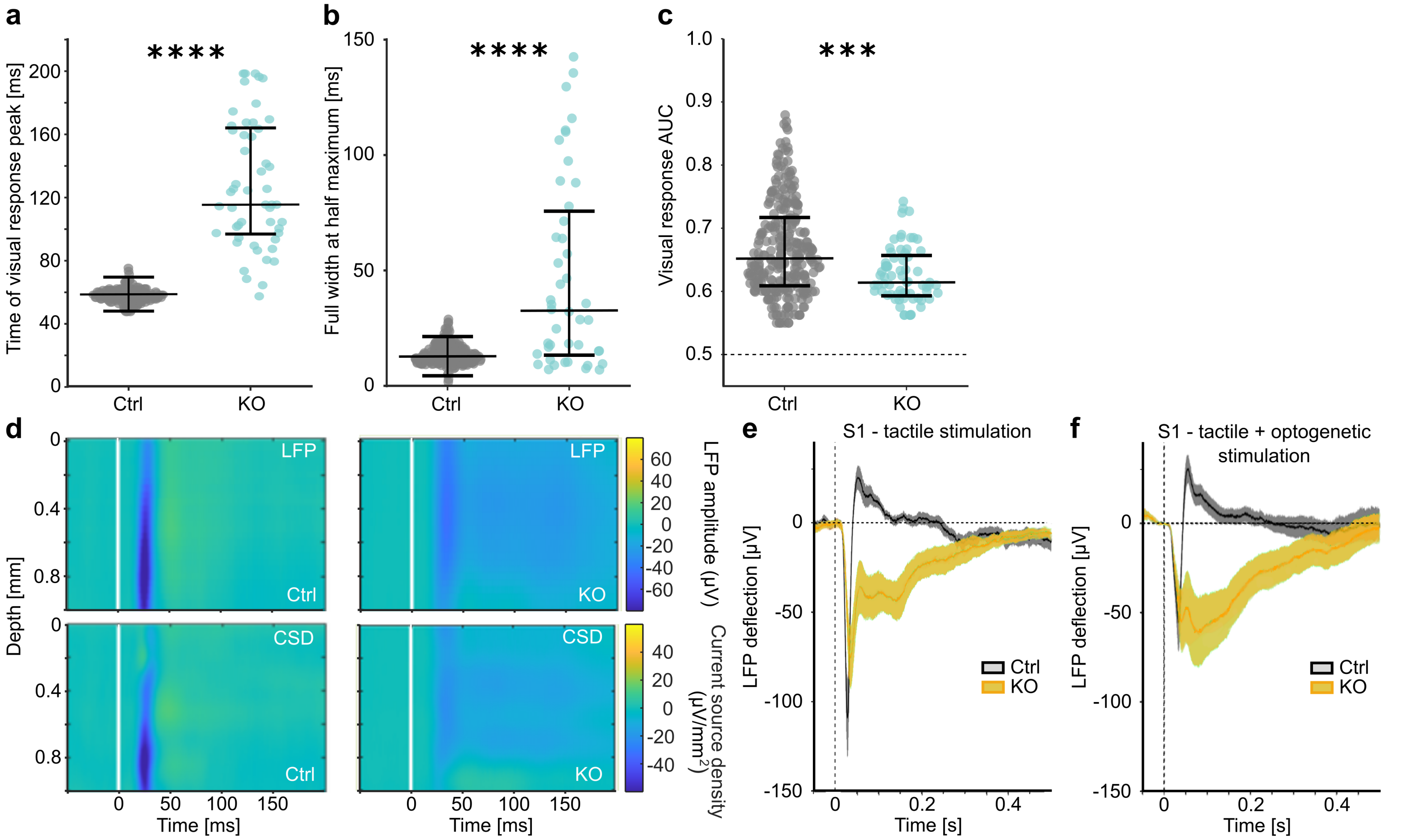
a
b
c
d
e
f
Time of visual response peak [ms]
Full width at half maximum [ms]
Visual response AUC
****
****
***
Ctrl
KO
Depth [mm]
Time [ms]
LFP
CSD
LFP amplitude (µV)
Current source density (µV/mm²)
S1 - tactile stimulation
S1 - tactile + optogenetic stimulation
LFP deflection [µV]
Time [s]

Supplementary Fig. 2

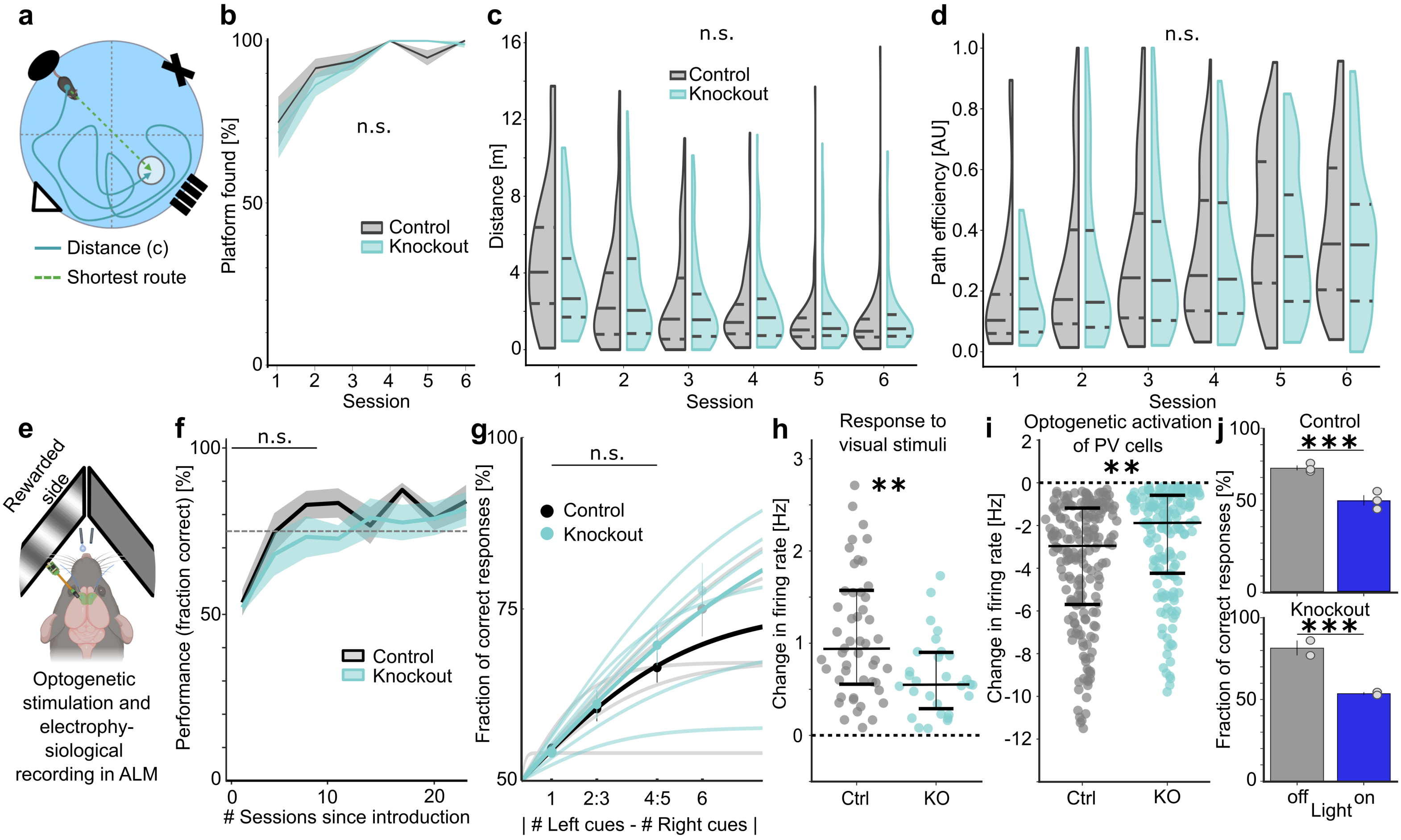

a
Distance (c)
Shortest route
b
Platform found [%]
n.s.
Control
Knockout
Session
c
n.s.
Control
Knockout
Distance [m]
Session
d
n.s.
Path efficiency [AU]
Session
e
Rewarded side
Optogenetic stimulation and electrophy-siological recording in ALM
f
n.s.
Performance (fraction correct) [%]
Control
Knockout
Sessions since introduction
g
n.s.
Control
Knockout
Fraction of correct responses [%]
| # Left cues - # Right cues |
h
Response to visual stimuli
**
Change in firing rate [Hz]
Ctrl
KO
i
Optogenetic activation of PV cells
**
Change in firing rate [Hz]
Ctrl
KO
j
Control
***
Knockout
***
Fraction of correct responses [%]
off
on
Light

Supplementary Fig. 3

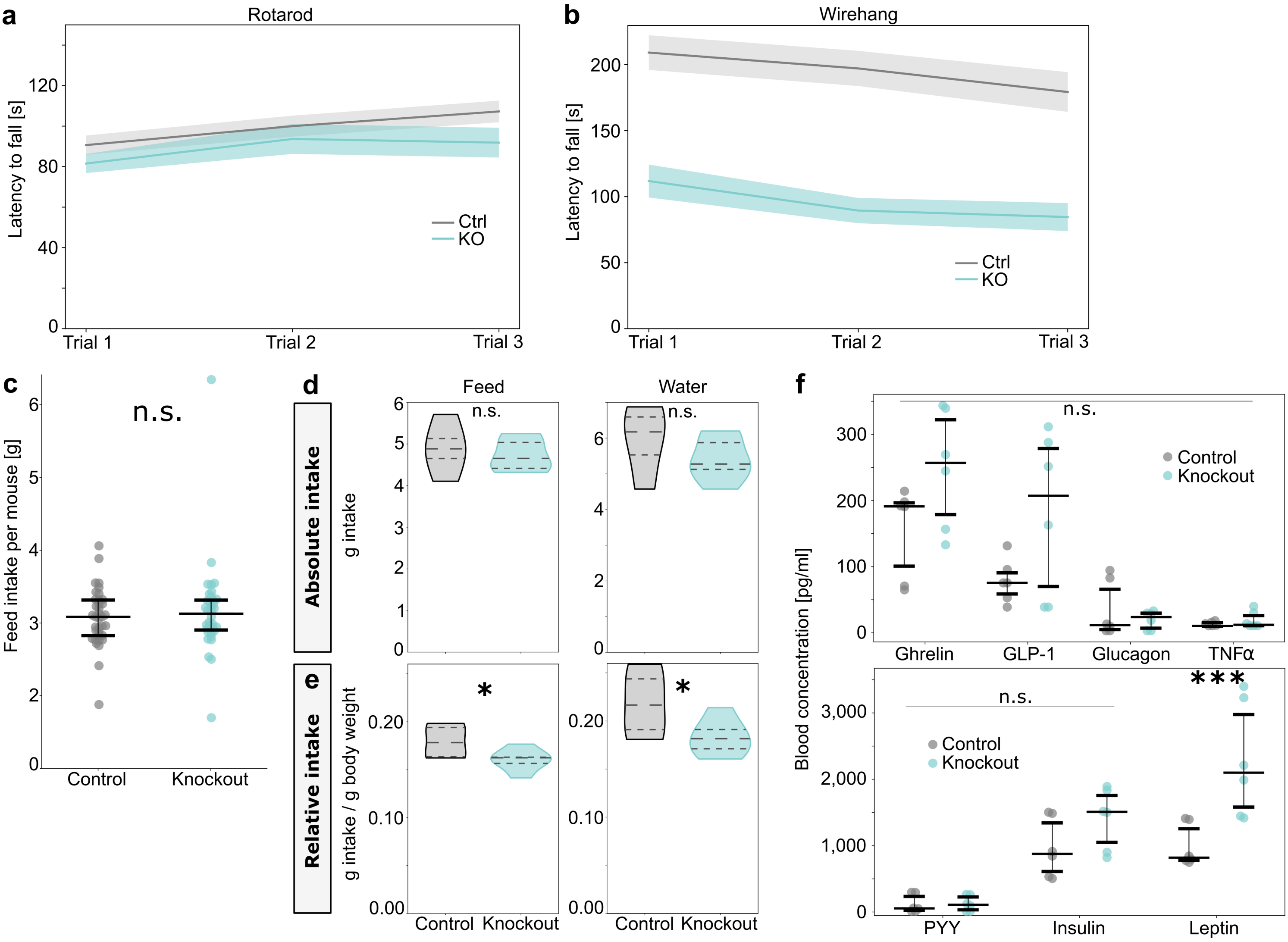

a
Rotarod
Latency to fall [s]
0
40
80
120
Trial 1
Trial 2
Trial 3
Ctrl
KO
b
Wirehang
Latency to fall [s]
0
50
100
150
200
Trial 1
Trial 2
Trial 3
Ctrl
KO
c
n.s.
Feed intake per mouse [g]
0
2
3
4
5
6
Control
Knockout
d
Absolute intake
Feed
Water
g intake
n.s.
n.s.
e
Relative intake
g intake / g body weight
*
*
0.00
0.10
0.20
Control
Knockout
Control
Knockout
f
n.s.
Control
Knockout
Ghrelin
GLP-1
Glucagon
TNFα
Blood concentration [pg/ml]
n.s.
***
Control
Knockout
PYY
Insulin
Leptin
0
1,000
2,000
3,000

Supplementary Fig. 4

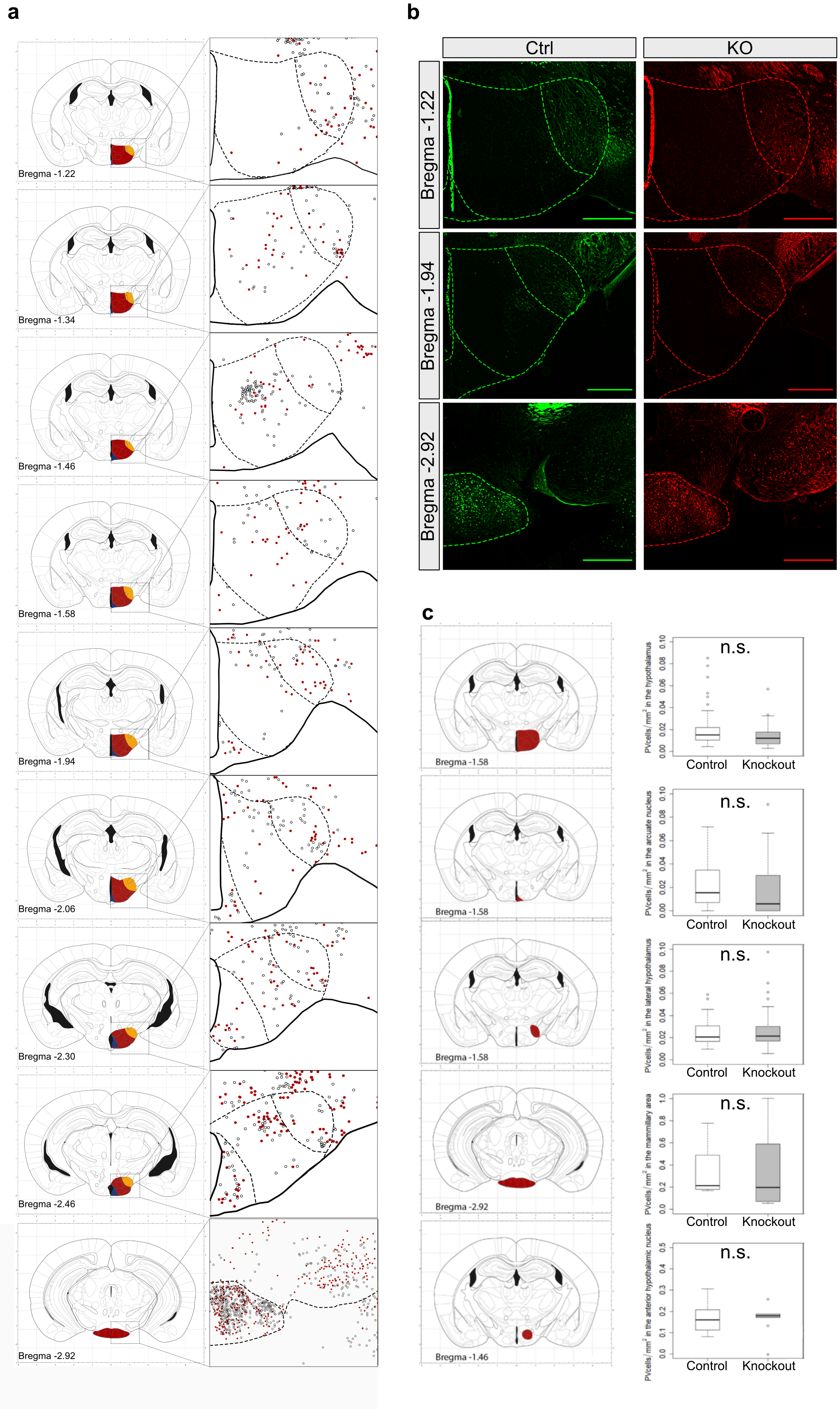
a
Bregma -1.22
Bregma -1.34
Bregma -1.46
Bregma -1.58
Bregma -1.94
Bregma -2.06
Bregma -2.30
Bregma -2.46
Bregma -2.92
b
Ctrl
KO
Bregma -1.22
Bregma -1.94
Bregma -2.92
c
Bregma -1.58
Bregma -1.58
Bregma -1.58
Bregma -2.92
Bregma -1.46
n.s.
PVcells / mm² in the hypothalamus
PVcells / mm² in the arcuate nucleus
PVcells / mm² in the lateral hypothalamus
PVcells / mm² in the mammillary area
PVcells / mm² in the anterior hypothalamic nucleus
Control
Knockout

Supplementary Fig. 5

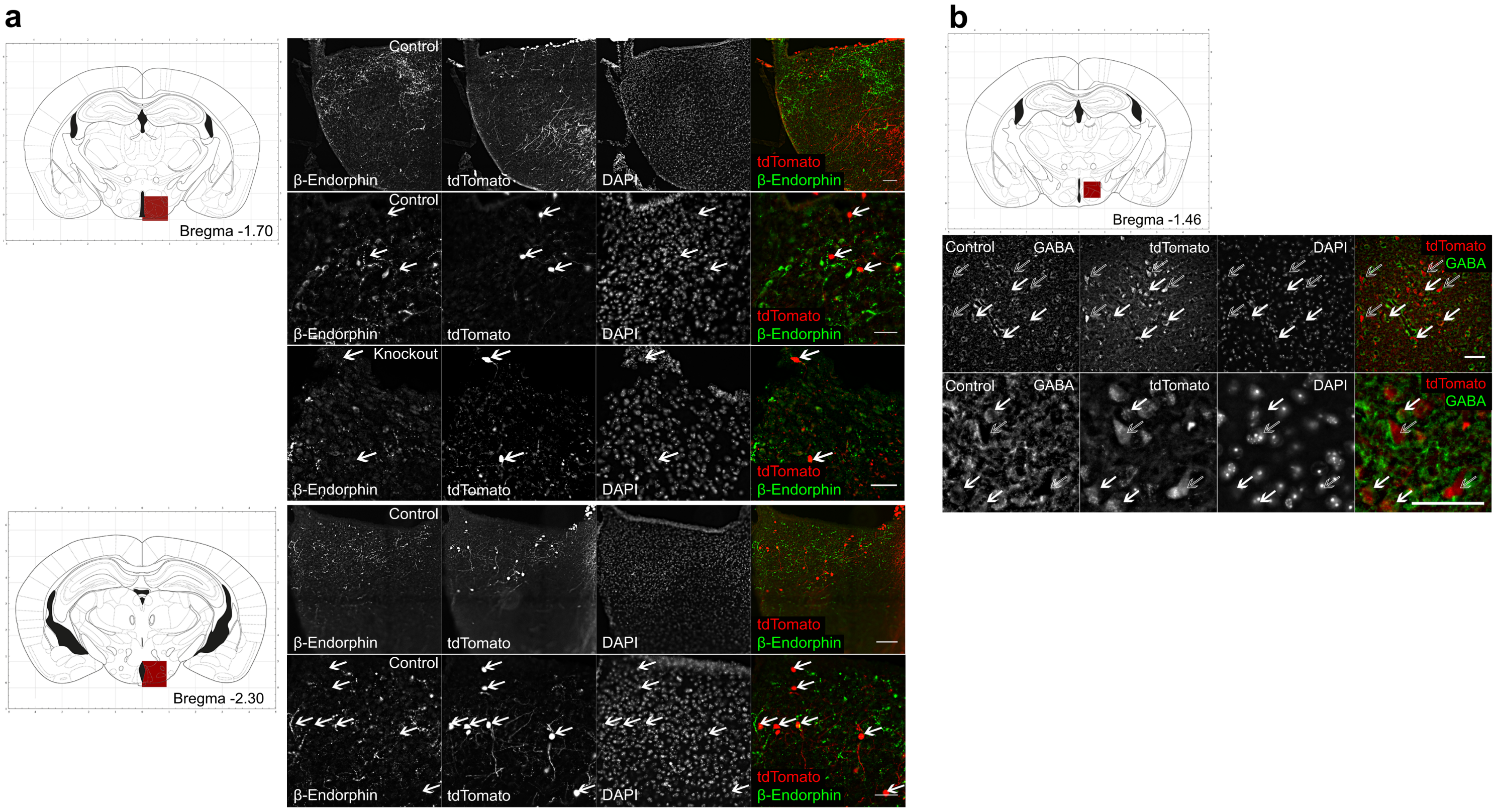

a
Bregma -1.70
Control
Knockout
β-Endorphin
tdTomato
DAPI
Bregma -2.30
b
Bregma -1.46
Control
GABA

Supplementary Fig. 6

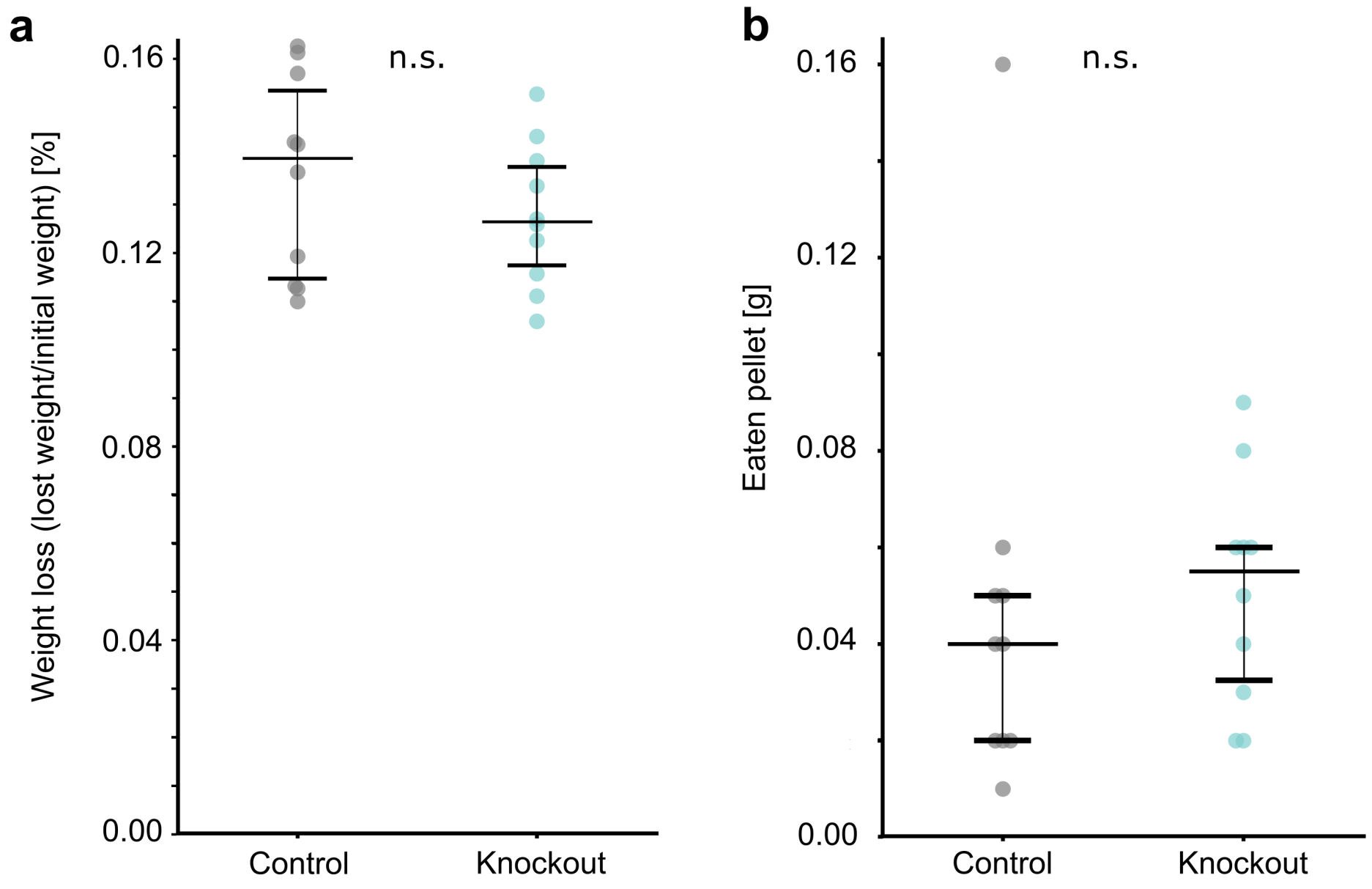
a
b
Weight loss (lost weight/initial weight) [%]
0.16
0.12
0.08
0.04
0.00
n.s.
Control
Knockout
Eaten pellet [g]
0.16
0.12
0.08
0.04
0.00
n.s.
Control
Knockout

Supplementary Fig. 7

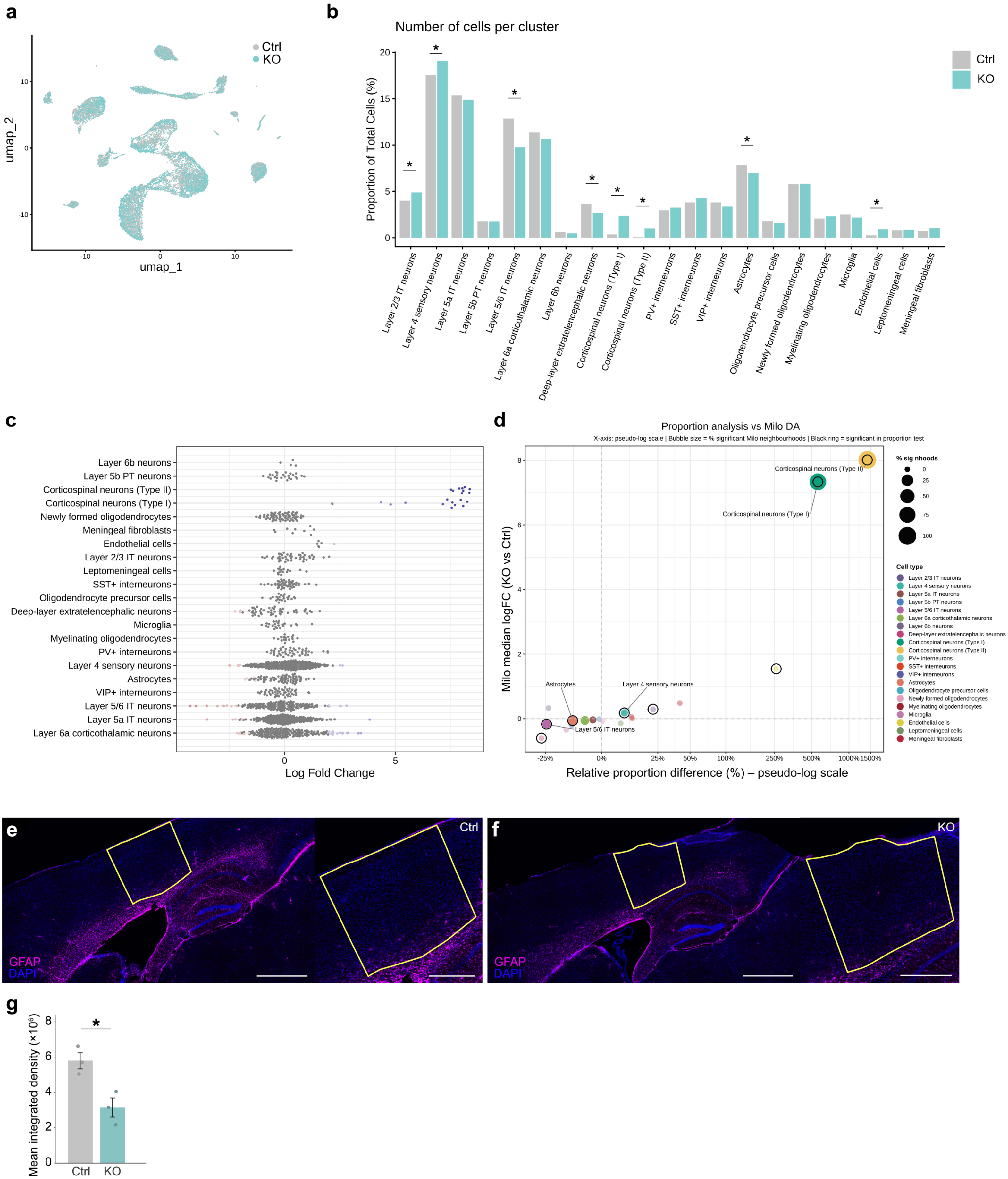
a
Ctrl
KO
umap_2
umap_1
b
Number of cells per cluster
Proportion of Total Cells (%)
Layer 2/3 IT neurons
Layer 4 sensory neurons
Layer 5a IT neurons
Layer 5b PT neurons
Layer 5/6 IT neurons
Layer 6a corticothalamic neurons
Layer 6b neurons
Deep-layer extratelencephalic neurons
Corticospinal neurons (Type I)
Corticospinal neurons (Type II)
PV+ interneurons
SST+ interneurons
VIP+ interneurons
Astrocytes
Oligodendrocyte precursor cells
Newly formed oligodendrocytes
Myelinating oligodendrocytes
Microglia
Endothelial cells
Leptomeningeal cells
Meningeal fibroblasts
c
Log Fold Change
d
Proportion analysis vs Milo DA
X-axis: pseudo-log scale | Bubble size = % significant Milo neighbourhoods | Black ring = significant in proportion test
Milo median logFC (KO vs Ctrl)
Relative proportion difference (%) – pseudo-log scale
% sig nhoods
Cell type
e
Ctrl
GFAP
DAPI
f
KO
GFAP
DAPI
g
Mean integrated density (×10⁶)
Ctrl
KO

Supplementary Fig. 8

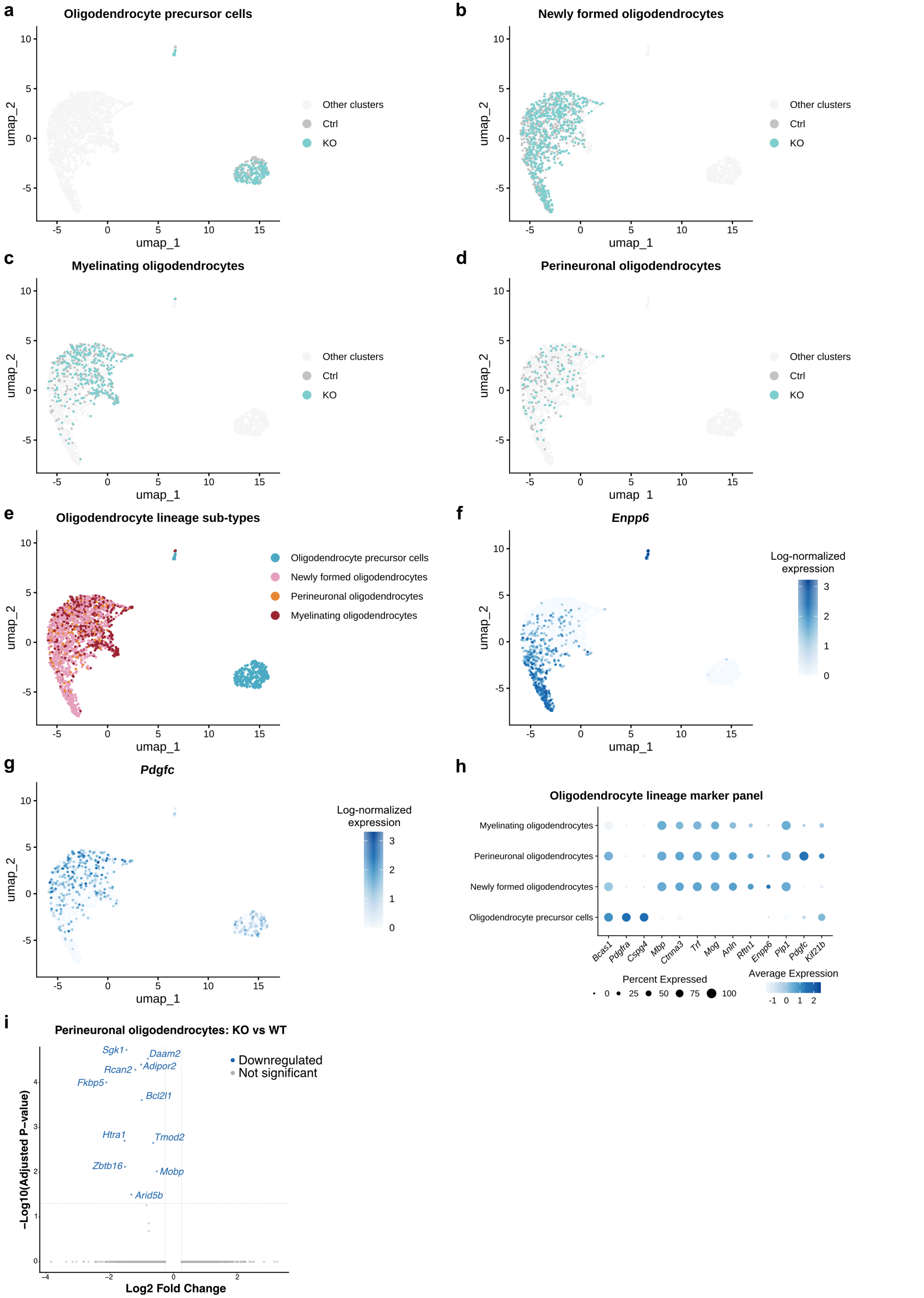
a
Oligodendrocyte precursor cells
Other clusters
Ctrl
KO
umap_1
umap_2
b
Newly formed oligodendrocytes
Other clusters
Ctrl
KO
umap_1
umap_2
c
Myelinating oligodendrocytes
Other clusters
Ctrl
KO
umap_1
umap_2
d
Perineuronal oligodendrocytes
Other clusters
Ctrl
KO
umap_1
umap_2
e
Oligodendrocyte lineage sub-types
Oligodendrocyte precursor cells
Newly formed oligodendrocytes
Perineuronal oligodendrocytes
Myelinating oligodendrocytes
umap_1
umap_2
f
Enpp6
Log-normalized expression
umap_1
umap_2
g
Pdgfc
Log-normalized expression
umap_1
umap_2
h
Oligodendrocyte lineage marker panel
Myelinating oligodendrocytes
Perineuronal oligodendrocytes
Newly formed oligodendrocytes
Oligodendrocyte precursor cells
Bcas1
Pdgfra
Cspg4
Mbp
Ctnna3
Trf
Mog
Anln
Rffn1
Enpp6
Plp1
Pdgfc
Kif21b
Percent Expressed
0
25
50
75
100
Average Expression
-1
0
1
2
i
Perineuronal oligodendrocytes: KO vs WT
Sgk1
Daam2
Rcan2
Adipor2
Fkbp5
Bcl2l1
Htra1
Tmod2
Zbtb16
Mobp
Arid5b
Downregulated
Not significant
-Log10(Adjusted P-value)
Log2 Fold Change

Supplementary Fig. 9

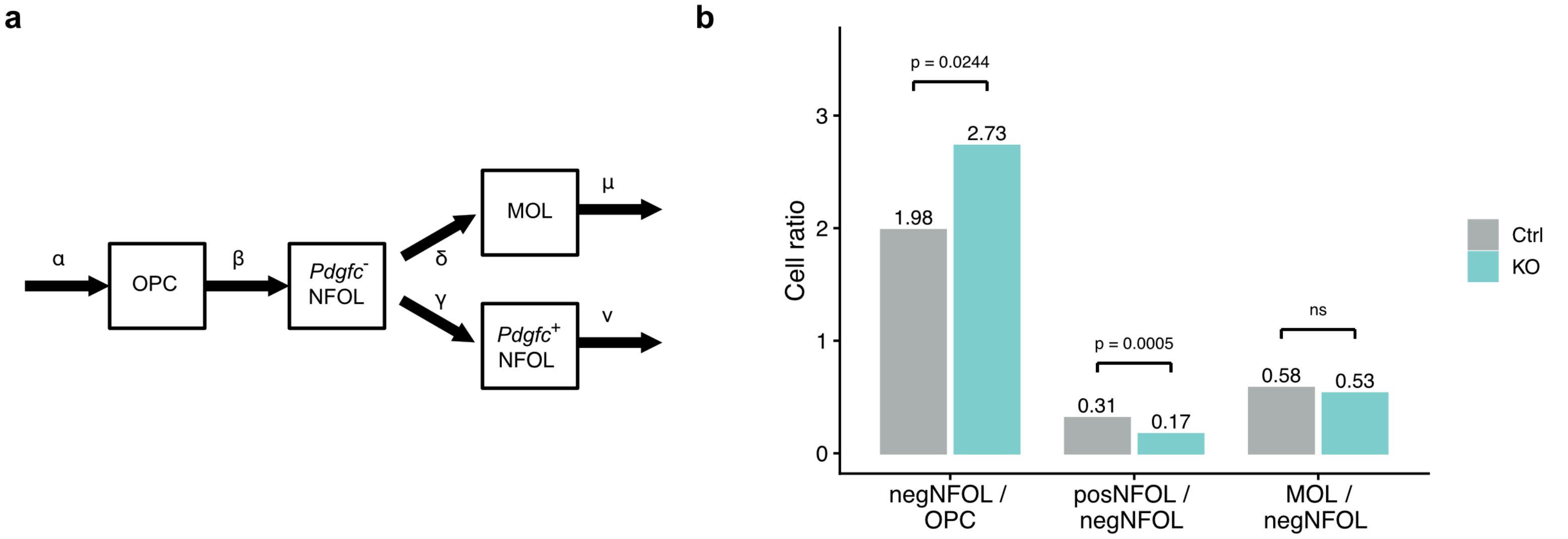
a
α
OPC
β
Pdgfc⁻
NFOL
δ
γ
MOL
μ
Pdgfc⁺
NFOL
ν
b
Cell ratio
p = 0.0244
2.73
1.98
p = 0.0005
0.31
0.17
ns
0.58
0.53
negNFOL / OPC
posNFOL / negNFOL
MOL / negNFOL
Ctrl
KO

Supplementary Fig. 10

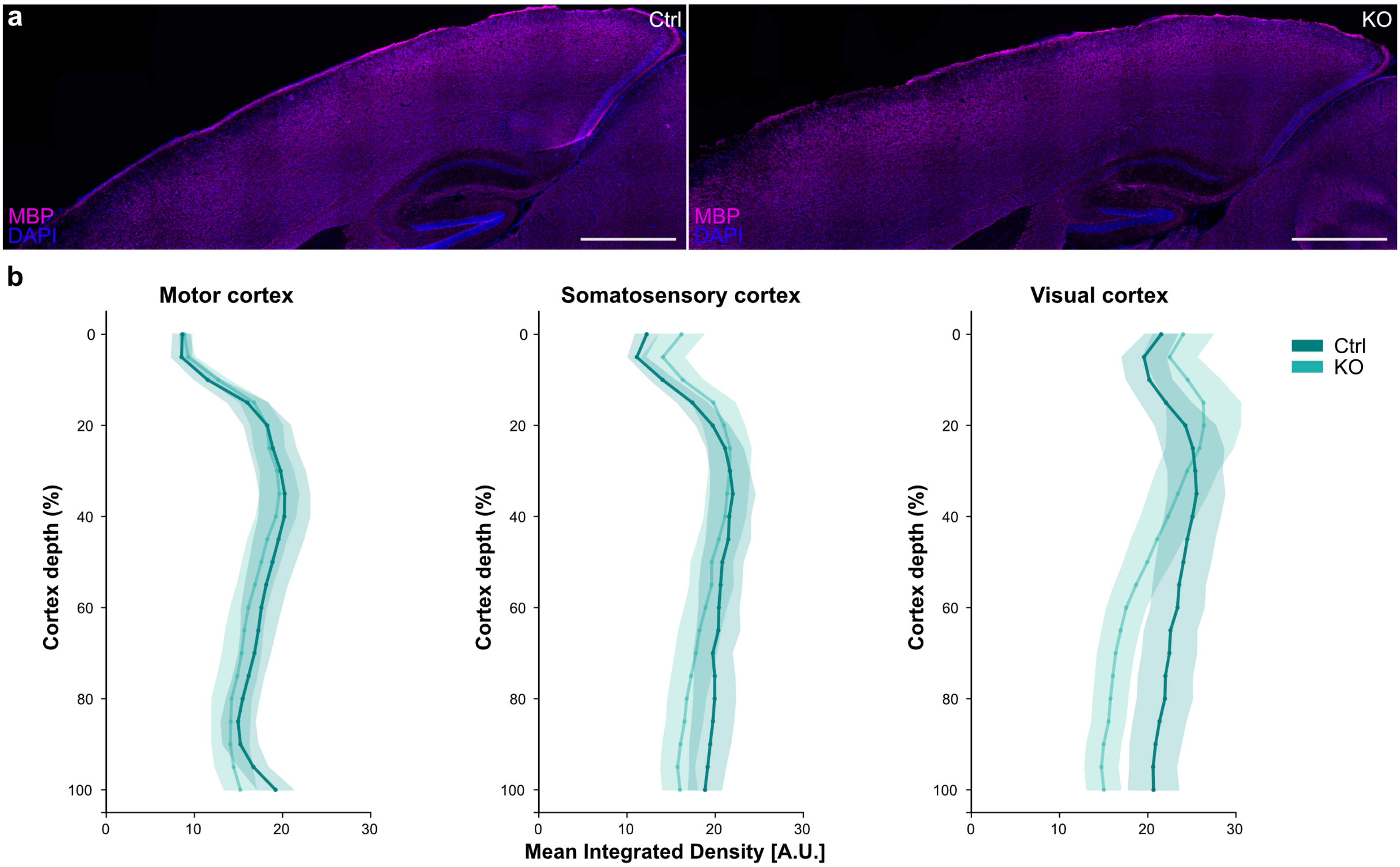
a
Ctrl
MBP
DAPI
KO
MBP
DAPI
b
Motor cortex
Somatosensory cortex
Visual cortex
Cortex depth (%)
0
20
40
60
80
100
0
10
20
30
Mean Integrated Density [A.U.]
Ctrl
KO

Supplementary Fig. 11

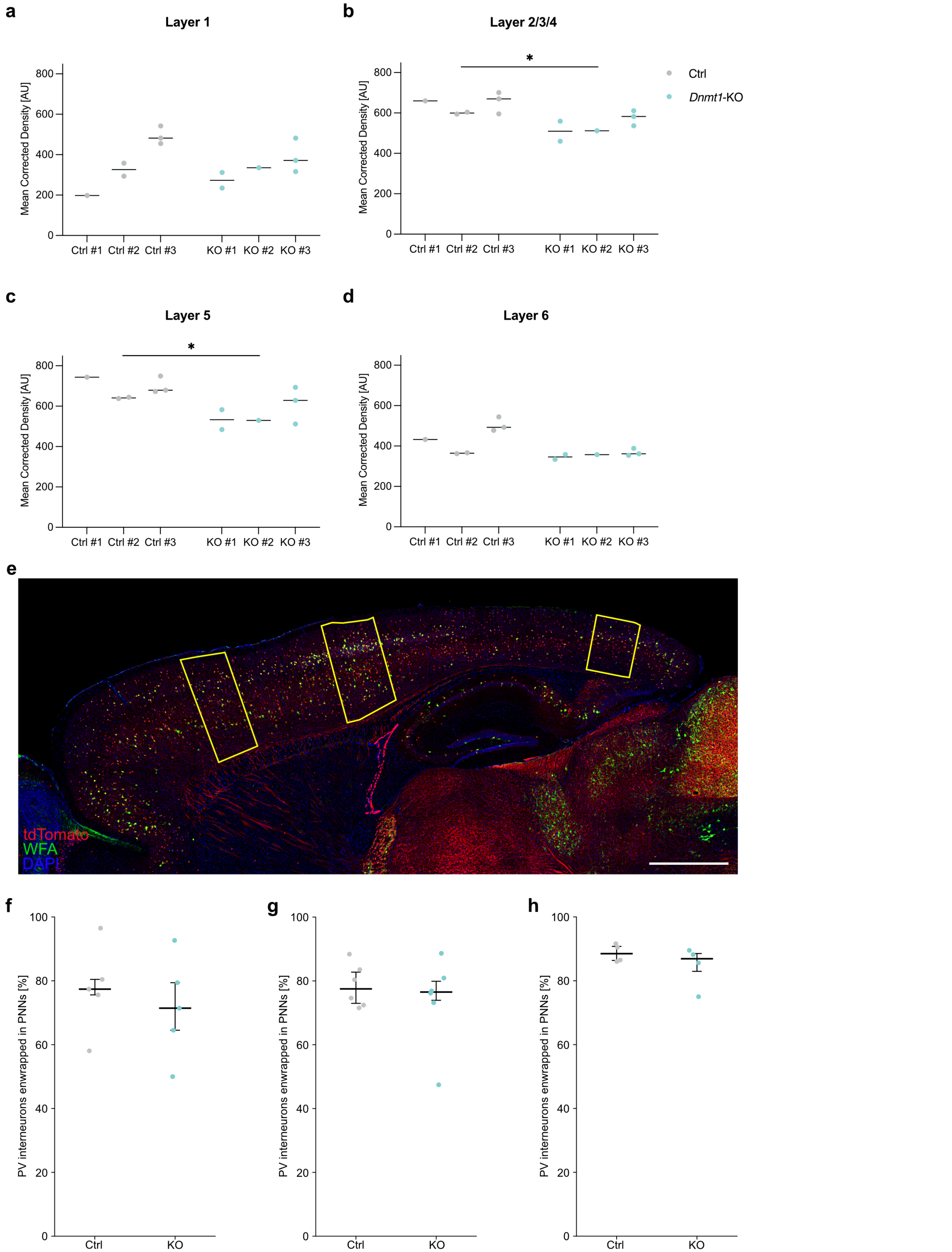
a
Layer 1
Mean Corrected Density [AU]
800
600
400
200
0
Ctrl #1
Ctrl #2
Ctrl #3
KO #1
KO #2
KO #3
b
Layer 2/3/4
*
Ctrl
Dnmt1-KO
c
Layer 5
d
Layer 6
e
tdTomato
WFA
DAPI
f
g
h
PV interneurons enwrapped in PNNs [%]
100
80
60
40
20
0
Ctrl
KO
