# Supplementary material for: DNMT1 function in cortical PV interneurons modulates cortical network activity, mood disorder‑related behavior, and perineuronal nets
Source: Commun Biol. 2026 Oct 8;9:1316. doi: 10.1038/s42003-026-11079-x (PMC13650159; doi:10.1038/s42003-026-11079-x)
Supplement: Supplementary file 3 [file 42003_2026_11079_MOESM3_ESM.pdf]

Description of Additional Supplementary Files:

File: Supplementary Data 1

Description: Any additional supporting data, including behavioral measurements, electrophysiological recordings, and histological analyses,are available under: https://doi.org/10.6084/m9.figshare.33752629 or as supplementary data files.

File: Supplementary Data 2

Description: Any additional supporting data, including behavioral measurements, electrophysiological recordings, and histological analyses,are available under: https://doi.org/10.6084/m9.figshare.33752629 or as supplementary data files.

File: Supplementary Data 3

Description: Any additional supporting data, including behavioral measurements, electrophysiological recordings, and histological analyses,are available under: https://doi.org/10.6084/m9.figshare.33752629 or as supplementary data files.

File: Supplementary Video 1

Description: Three-dimensional reconstruction of a PV interneuron and its perineuronal net.

Rotating 3D rendering of a parvalbumin-positive (PV) interneuron (red) from a Pvalb Cre/tdTom (Ctrl) mouse, surrounded by its perineuronal net (PNN) labeled with Wisteria floribunda agglutinin (WFA, green). The video illustrates the spatial relationship and enwrapping pattern of the PNN around the soma and proximal dendrites.

File: Supplementary Video 2

Description: Three-dimensional reconstruction of a Dnmt1-deficient PV interneuron and its perineuronal net.

Rotating 3D rendering of a PV interneuron (red) from a Pvalb-Cre/tdTom/Dnmt1 loxP2(KO) mouse and its PNN labeled with WFA (green). The video highlights the perturbations in PNN organization and coverage compared to the control condition shown in Supplementary Video 1.
